# Common Genetic Variants Modulate the Electrocardiographic Tpeak-to-Tend Interval

**DOI:** 10.1016/j.ajhg.2020.04.009

**Published:** 2020-05-07

**Authors:** Julia Ramírez, Stefan van Duijvenboden, William J. Young, Michele Orini, Pier D. Lambiase, Patricia B. Munroe, Andrew Tinker

**Affiliations:** 1 Clinical Pharmacology, William Harvey Research Institute, Barts and The London School of Medicine and Dentistry, Queen Mary University of London, London EC1M 6BQ, UK; 2 Institute of Cardiovascular Science, University College London, London WC1E 6BT, UK; 3 Barts Heart Centre, St Bartholomew’s Hospital, London EC1A 7BE, UK; 4 NIHR Barts Cardiovascular Biomedical Research Unit, Barts and The London School of Medicine and Dentistry, Queen Mary University of London, London EC1M 6BQ, UK

**Keywords:** genetics, genome-wide association study, ventricular repolarization, ventricular arrhythmias, T-peak-to-T-end interval, sudden cadiac death, electrocardiogram, cardiac conduction and contraction, genetic risk score, cardiovascular dynamics

## Abstract

Sudden cardiac death is responsible for half of all deaths from cardiovascular disease. The analysis of the electrophysiological substrate for arrhythmias is crucial for optimal risk stratification. A prolonged T-peak-to-Tend (Tpe) interval on the electrocardiogram is an independent predictor of increased arrhythmic risk, and Tpe changes with heart rate are even stronger predictors. However, our understanding of the electrophysiological mechanisms supporting these risk factors is limited. We conducted genome-wide association studies (GWASs) for resting Tpe and Tpe response to exercise and recovery in ∼30,000 individuals, followed by replication in independent samples (∼42,000 for resting Tpe and ∼22,000 for Tpe response to exercise and recovery), all from UK Biobank. Fifteen and one single-nucleotide variants for resting Tpe and Tpe response to exercise, respectively, were formally replicated. In a full dataset GWAS, 13 further loci for resting Tpe, 1 for Tpe response to exercise and 1 for Tpe response to exercise were genome-wide significant (p ≤ 5 × 10^−8^). Sex-specific analyses indicated seven additional loci. In total, we identify 32 loci for resting Tpe, 3 for Tpe response to exercise and 3 for Tpe response to recovery modulating ventricular repolarization, as well as cardiac conduction and contraction. Our findings shed light on the genetic basis of resting Tpe and Tpe response to exercise and recovery, unveiling plausible candidate genes and biological mechanisms underlying ventricular excitability.

## Introduction

Sudden cardiac death is a leading cause of mortality and is responsible for approximately half of all deaths from cardiovascular disease.^1^ Most importantly, the vast majority of sudden cardiac deaths occur in the general population without known traditional risk factors.^2^ Guidelines exist for preventive strategies, such as insertion of implantable cardioverter defibrillators in high-risk patient groups.^3^ However, risk stratification is heavily reliant on the assessment of left ventricular systolic function, which has low specificity, as opposed to the analysis of the electrophysiological substrate for arrhythmias.

The surface electrocardiogram (ECG) is a widely available non-invasive tool, which provides a rapid assessment of underlying cardiac electrophysiology and is therefore a useful method to infer arrhythmic risk. An abnormally prolonged Tpeak-to-Tend (Tpe) interval on the ECG is a risk factor for ventricular arrhythmic mortality and all-cause mortality, independent of age, sex, comorbidities, QRS duration, and corrected QT interval (MIM: 610141), not only in healthy subjects^4^ but also individuals with acquired QT prolongation^5^^,^^6^ and cardiac patients.^7^, ^8^, ^9^, ^10^, ^11^, ^12^ In addition, the response of the Tpe interval to heart rate has also been reported to be significantly associated with sudden cardiac death in patients with heart failure.^13^^,^^14^

Although the general view is that the Tpe interval and the T-wave more commonly reflect spatial dispersion of repolarization in different regions of the heart, the exact nature of this is disputed.^15^, ^16^, ^17^ One pre-eminent suggestion is that it reflects differences in transmural repolarization, but this is largely based on the *ex vivo* ventricular wedge preparation and has not been reproduced in the intact heart.^16^^,^^18^^,^^19^ Thus, novel approaches are needed to improve our understanding of the biology underpinning T-wave morphology and specifically Tpe in the intact human heart.

Prior work in twin studies has demonstrated that resting Tpe interval is heritable (52%–63%)^20^ and, consequently, genetic analyses have been undertaken to uncover its genetic determinants, identifying five loci^21^^,^^22^ (Table S1). However, no genome-wide association study (GWAS) has been performed for resting Tpe interval in relatively large cohorts (>6,000 individuals) and the genetic basis of Tpe response to exercise and to recovery has never been studied.

**Figure 1:**
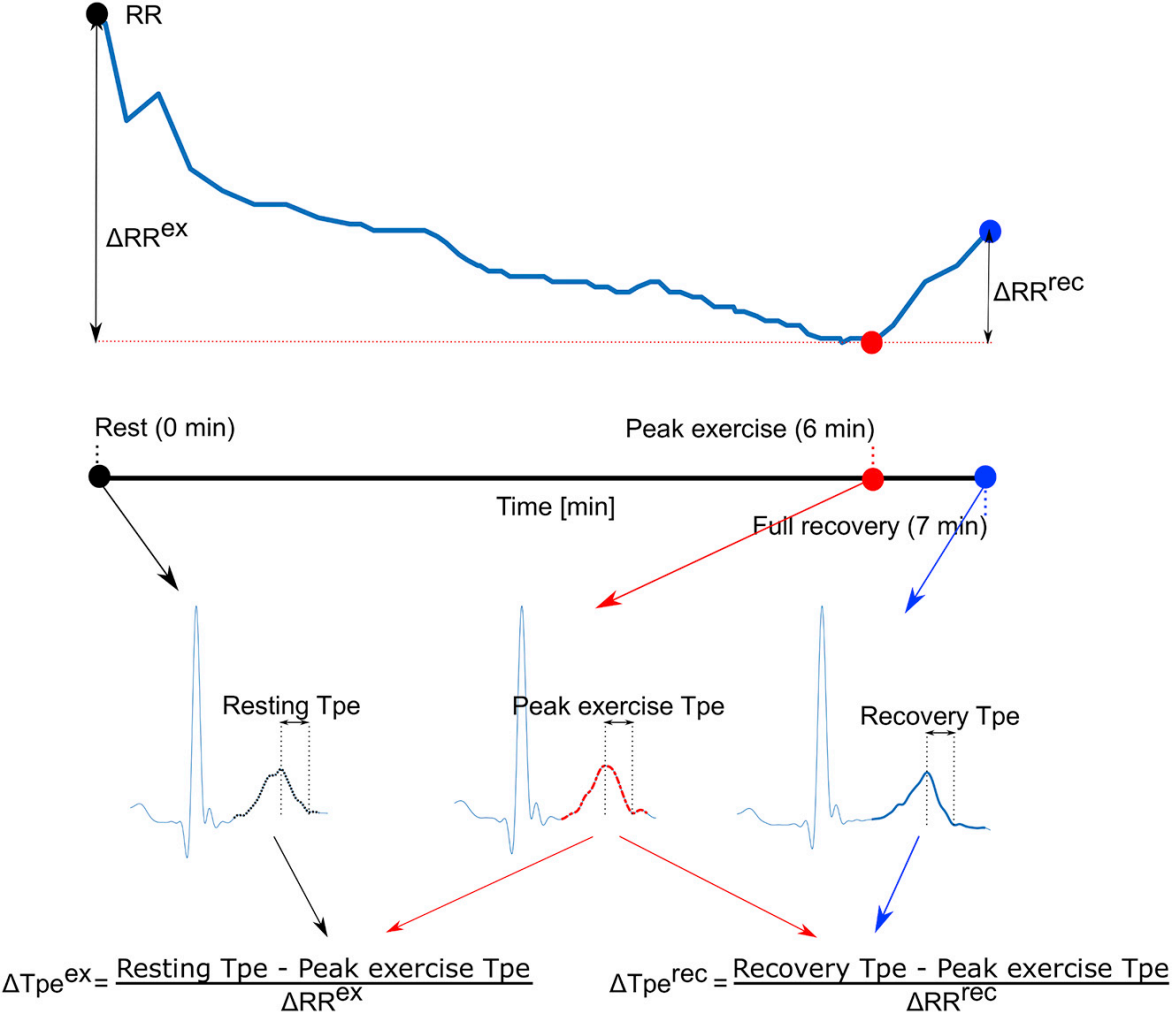
Assessment of Tpe Indices in the EST-UKB Cohort (Top) Illustration of the RR profile during the exercise stress test. (Bottom) Three averaged heartbeats are derived at rest (black filled circle), peak exercise (red filled circle), and full recovery (blue filled circle), respectively. Resting, peak exercise, and recovery Tpe intervals were derived as the temporal differences between the corresponding T-wave offset and T-wave peak timing locations. Tpe dynamics during exercise was derived by quantifying the difference between the Tpe intervals at rest (black T-wave) and at peak exercise (red T-wave), normalized by the RR change during this interval. Similarly, Tpe dynamics during recovery was derived by quantifying the difference between the Tpe intervals at peak exercise (red T-wave) and full recovery (blue T-wave), normalized by the subsequent RR change.

Our objective was to identify genetic variants significantly associated with three traits (Figure 1) in a large middle-aged population from the UK: (1) resting Tpe interval (n = 71,338), (2) Tpe response to exercise (n = 51,897), and (3) Tpe response to recovery (n = 51,503). We applied extensive bioinformatics analyses to investigate the main biological pathways linking the identified loci and the three traits.

## Material and Methods

Anonymized data and materials have been returned to UK Biobank (UKB) and can be accessed per request.

### UK Biobank

UKB is a prospective study of 488,377 volunteers comprising relatively even numbers of men and women aged 40–69 years old at recruitment (2006–2008). The UKB study has approval from the North West Multi-Centre Research Ethics Committee, and all participants provided informed consent.^23^ The work was undertaken as part of UKB application 8256.

Genotyping was performed by UKB using the Applied Biosystems UK BiLEVE Axiom Array or the UKB AxiomTM Array.^24^ Single-nucleotide variants (SNVs) were imputed centrally by UKB using the Haplotype Reference Consortium (HRC) and the 1000 Genomes Project (1000G) reference panels. Information on UKB array design and protocols is available on the UKB website (see Web Resources).

Participants were genotyped using a customized array (including GWAS and exome content) and with genome-wide imputation based on HRC and 1000G sequencing data.^25^ A sub-cohort of 58,839 individuals completed an exercise test using a stationary bicycle in conjunction with an ECG recording (lead I, 2009, EST-UKB cohort). In parallel, a sub-cohort of 35,225 individuals participated in an imaging study (05/2014–03/2019; the collection is ongoing, IMAGE-UKB), which included 10 s 12-lead ECG recordings. All ECGs were acquired following the same protocol (see UKB website in Web Resources) and analyzed with the methods explained below.

### Phenotypic and Genetic QC

Detailed information about the phenotypic and genetic quality control (QC) are indicated in Figure S1 and Supplemental Methods. Of the 56,385 individuals from EST-UKB who passed the phenotypic QC, 52,147 complied with genetic QC and were of European ancestry. Similarly, of the 26,467 individuals from IMAGE-UKB who passed the phenotypic QC, 24,999 complied with genetic QC and were of European ancestry. Then, 5,569 individuals who were in both the EST-UKB and IMAGE-UKB cohorts were excluded from the IMAGE-UKB cohort. After exclusions, there were 52,147 individuals from the EST-UKB cohort and 19,430 individuals from the IMAGE-UKB cohort available for genetic analyses (Figure S1).

### Derivation of Resting Tpe and Tpe Response to Exercise and Recovery from the EST-UKB Cohort

The bicycle ergometer test followed a standardized protocol of 15 s resting period, followed by 6 min of exercise during which the workload was gradually increased, and a 1-min recovery period without pedalling. Pre-processing of the ECG signals from the EST-UKB cohort included low-pass filtering at 50 Hz to remove electric and muscle noise but still allow QRS detection.^26^ Baseline wander was removed by further high-pass filtering the ECG signals at 0.5 Hz. Automatic quantification of resting Tpe and Tpe response to exercise and recovery (shown in Figure 1) was performed on every ECG recording in three steps:

(1) We signal-averaged the heartbeats within a window of 15 s during rest (black), at peak exercise (red), and 50 s after peak exercise (blue) to attenuate noise and artifacts and reveal small variations in the QRS-T-waveform. The onset, peak, and offset timings of the waveforms were located using bespoke software.^16^^,^^27^
(2) We derived resting, peak exercise, and recovery Tpe intervals as the temporal differences between the corresponding T-wave end and T-wave peak timing locations.
(3) Tpe response to exercise was derived by quantifying the difference between the Tpe intervals at rest (black T-wave) and at peak exercise (red T-wave), normalized by the RR change during this interval, $\Delta RR^{ex}$. Similarly, Tpe response to recovery was derived by quantifying the difference between the Tpe intervals at peak exercise (red T-wave) and full recovery (blue T-wave), normalized by the subsequent RR change, $\Delta RR^{rec}$.

### Derivation of Resting Tpe from the IMAGE-UKB Cohort

We chose lead I for analysis in the IMAGE-UKB cohort to match the EST-UKB signal. We removed baseline wander using a publicly available algorithm (see Web Resources). We pre-processed and signal averaged the heartbeats in the 10 s recordings as in the EST-UKB cohort. The onset, peak, and end timings of the waveforms were located using the same bespoke software as in previous studies.^16^^,^^27^ Resting Tpe was derived as the temporal difference between the T-wave end and the T-wave peak timing locations.

**Figure 2:**
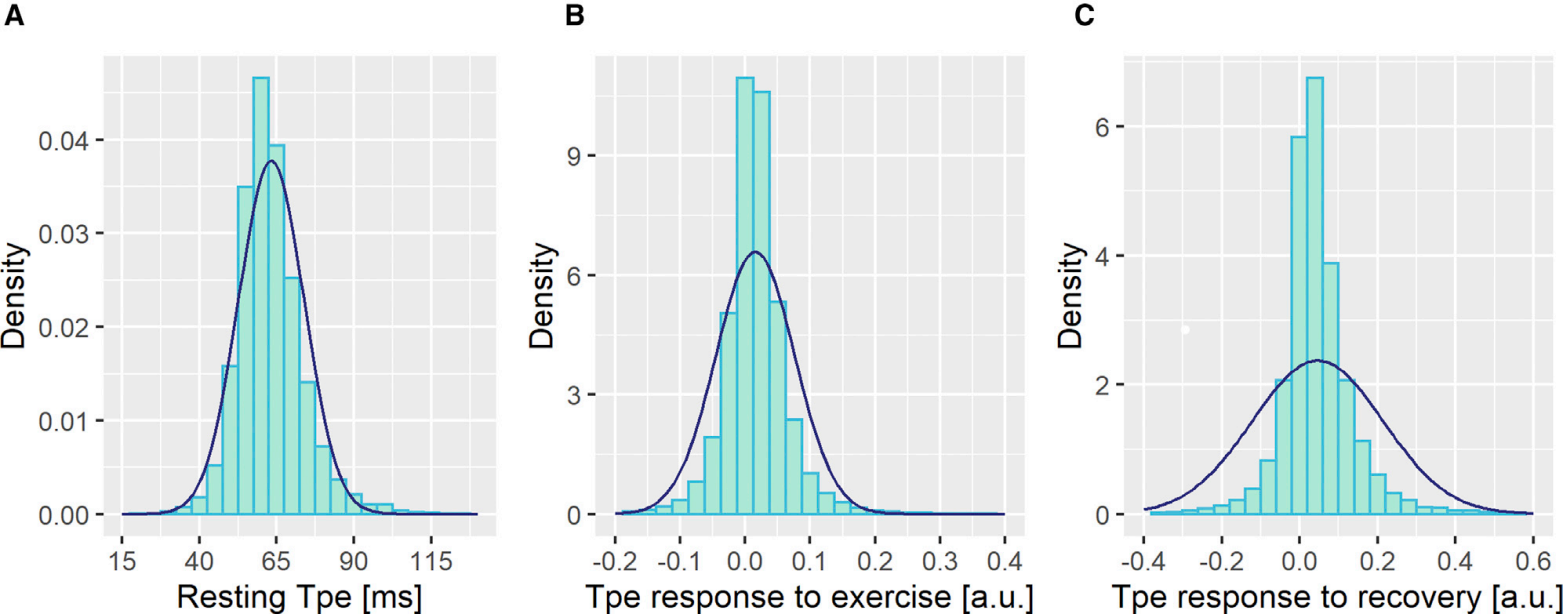
Density Plots of Tpe Phenotypes Resting Tpe (A), Tpe dynamics during exercise (B), and Tpe dynamics during recovery (C). The blue curves indicate a normal distribution using the mean and standard deviation from each distribution.

For resting Tpe, we pooled the measurements from both EST-UKB and IMAGE-UKB cohorts together, leading to 71,338 individuals with resting Tpe. Inverse-normal transformation of resting Tpe and Tpe response to exercise and recovery was performed, as the distributions were skewed (Figure 2).

### Genetic Analyses

**Figure 3:**
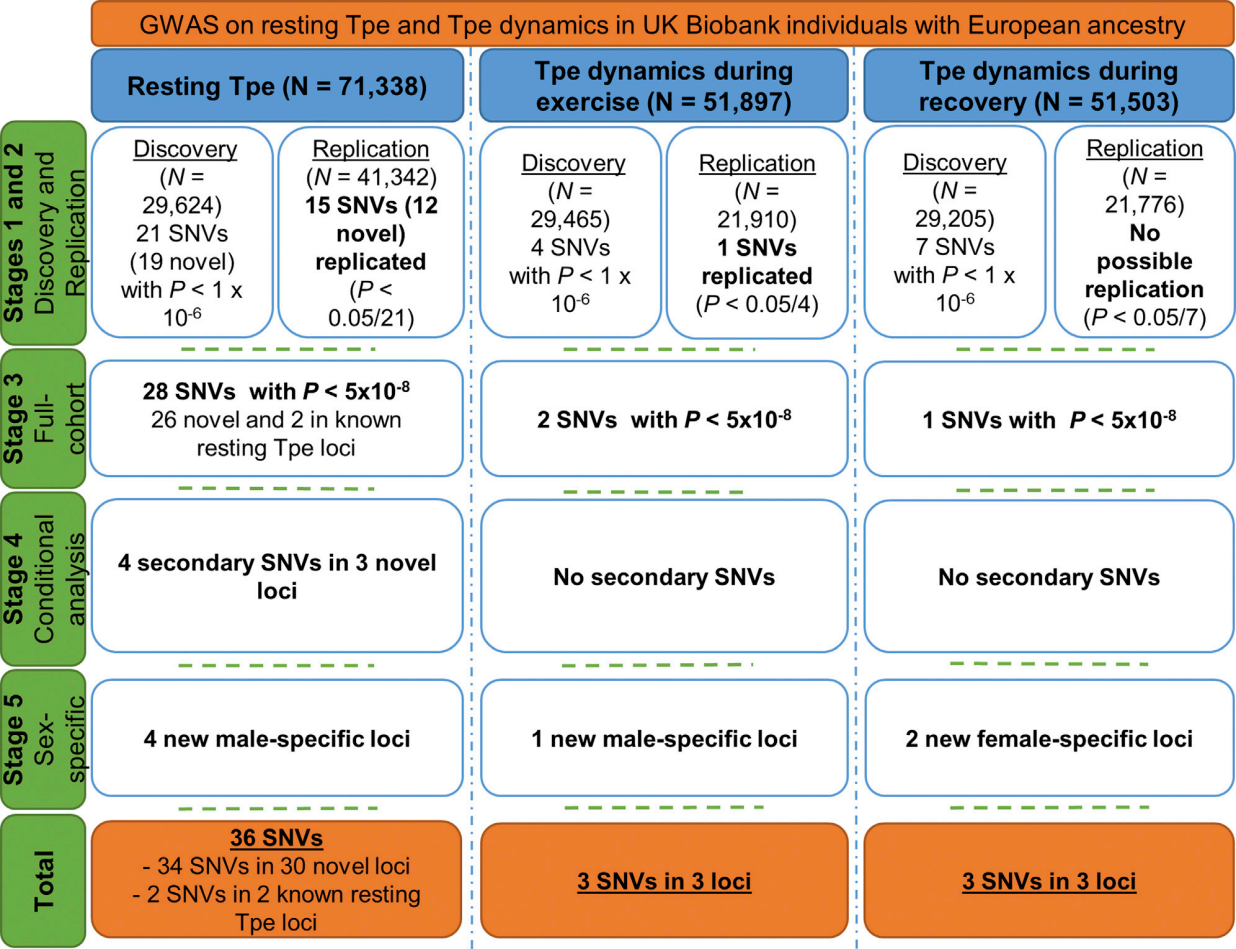
Analytical 5-Stage Approach Flowchart Tpe, T-peak-to-Tend interval; SNV, single-nucleotide variant. Additional information can be found in Material and Methods.

An overview of the study design is provided in Figure 3. We randomly divided our cleaned datasets into discovery (n ≈ 30,000) and replication (n ≈ 42,000 for resting Tpe and n ≈ 22,000 for Tpe response to exercise and to recovery) datasets. To ensure that there was no overlap across datasets, we removed first- and second-degree related individuals (kinship coefficient > 0.88) from the replication cohort as indicated from UKB.^24^ We next selected model SNVs from directly genotyped SNVs using PLINK 1.9.^28^ This selection was based on the following criteria: minor allele frequency (MAF) > 5%, a Hardy-Weinberg equilibrium with a threshold of p value = 1 × 10^−6^, and missingness < 0.0015. Model SNVs are used to learn the parameters from the mixed models for both the heritability estimation and the GWASs (see below).

Then, we estimated the proportion of resting Tpe, and Tpe response to exercise and recovery explained by additive genetic variation (heritability), as well as their genetic correlation with each other, using a variance components method (BOLT-REML),^29^ with the model SNVs and ∼9 million imputed variants with MAF ≥ 1% and INFO > 0.3 using the full cohorts (Figure 3).

Next, we performed a GWAS for each trait using a linear mixed model method (BOLT-LMM)^30^ under the additive genetic model, including the model SNVs and ∼9 million imputed SNVs with MAF ≥ 1% and INFO > 0.3 in the discovery dataset (Figure 3). For resting Tpe, we included the following covariates (details can be found in the Supplemental Methods): sex, age, body mass index (BMI), smoking status, resting RR, and a binary indicator variable for the genotyping array (UKB versus UK BiLEVE). For Tpe response to exercise, we included sex, age, BMI, diabetic status, resting RR, $\Delta RR^{ex}$, and the genetic array. For Tpe response to recovery, we included sex, age, BMI, diabetic status, recovery RR, $\Delta RR^{rec}$, and the genetic array.

### Replication Analyses

All SNVs with p < 1 × 10^−6^ from the discovery GWAS for each trait were compiled and mapped to individual loci based on a genomic distance of >500 Kb to each side of another SNV. If multiple SNVs fitted the selection criteria for a single region, only the SNV with the smallest p value was taken forward into replication. As a QC step, we reviewed each selected SNV to check for unrealistically high effect sizes or large standard errors, and none were observed. Regional plots were produced for all selected SNVs and these were carefully reviewed. Twenty-one variants for resting Tpe, 4 variants for Tpe response to exercise, and 7 variants for Tpe response to recovery were taken forward into replication. Replication was confirmed if p ≤ 0.05/21 = 2.4 × 10^−3^ for resting Tpe, p ≤ 0.05/4 = 1.3 × 10^−2^ for Tpe response to exercise, and p ≤ 0.05/7 = 7.1 × 10^−3^ for Tpe response to recovery and the effect was in the direction observed in discovery analyses for each trait in the replication cohort (Figure 3).

### Full Dataset Analyses

In addition to the two-stage study design of discovery and replication cohorts, we also conducted a full dataset GWAS including all individuals (n = 71,338 for resting Tpe, n = 51,897 for Tpe response to exercise, and n = 51,503 for Tpe response to recovery) using BOLT-LMM.^30^ Additional loci for each trait reaching a genome-wide significance threshold (p ≤ 5 × 10^−8^) from the full dataset GWAS are reported (Figure 3). To test for polygenicity, any underlying residual population stratification or QC factors affecting our GWAS results, we run LD Score Regression.^31^

### Conditional Analyses

To examine whether there were independent SNVs at these loci, we applied genome-wide complex trait analysis.^32^ We declared a secondary signal if (1) the identified SNV original p value was lower than 1 × 10^−6^; (2) there was less than a 1.5-fold difference between the lead SNV and secondary association p values on a –log_10_ scale, i.e., if –log_10_(p_*lead*_)/−log_10_(p_sec_) < 1.5; and (3) there was less than a 1.5-fold difference between the main association and conditional association p values on a –log_10_ scale, i.e., if –log_10_(p_*sec*_)/−log_10_(p_cond_) < 1.5.^33^

### Sex-Stratified Analyses

For each trait, we performed a GWAS for men and women separately in the full cohort including the same covariates in the regression model as specified above, but excluding sex (Figure 3).

### Percent Variance Explained

The percent variance explained of each variant was calculated by estimating the residuals from the regression model against the covariates used in each respective genetic model. We then fitted a second linear model for the trait residuals with all the identified variants plus the top ten principal components. The percent variance explained was the difference between the adjusted R-squared parameters from each model.^33^

### Bioinformatics Analyses

We performed several analyses to annotate the identified SNVs, at the variant and gene level (all SNVs in linkage disequilibrium [LD], r^2^ ≥ 0.8 with the traits associated SNVs were considered). LD was calculated using genetic data from UKB in order to calculate pairwise-LD for all associated SNVs. The r^2^ of pairwise SNVs (minimum r^2^ = 0.8 and maximum distance between a pair of SNVs is 4 Mb) were computed using PLINK.^28^

Using the University of California, Santa Cruz known genes, we annotated each lead SNV with the nearest coding genes and those located within 50 kb. At the variant level, we used Variant Effect Predictor^34^ to obtain comprehensive functional characterization of variants, including their gene location, conservation, and amino acid substitution impact based on a range of prediction tools including SIFT and PolyPhen-2.

We evaluated all SNVs in LD (r^2^ ≥ 0.8) with our validated lead SNVs for evidence of mediation of expression quantitative trait loci (eQTL) using the GTEx database, focusing on loci with the strongest evidence of eQTL associations in brain, heart, and adrenal tissue. We also performed colocalization analyses using COLOC^35^ including all SNVs within all loci with evidence of eQTLs in the relevant tissues and analyzed each eQTL-GWAS dataset pair. This tool is based on a Bayesian statistical methodology that tests pairwise colocalization of SNVs in GWAS with eQTLs and generates posterior probabilities for each locus weighting the evidence for competing hypothesis of either no colocalization or sharing of a distinct SNV at each locus. A posterior probability of ≥75% was considered strong evidence of the tissue-specific eQTL-GWAS pair influencing both the expression and GWAS trait at a particular region. We, then, identified variants with regulatory potential using RegulomeDB^36^ and found genes whose promoter regions form significant chromatin interaction with them from a range of tissues, including brain, heart, and adrenal long-range chromatin interaction (Hi-C) data. We found the most significant promoter interactions for all potential regulatory SNVs (RegulomeDB score ≤ 5) in LD (r^2^ ≥ 0.8) with our sentinel SNVs and chose the interactors with the SNVs of highest regulatory potential to annotate the loci.

We also performed enrichment testing across all loci. We used DEPICT^37^ to identify cells and tissues in which resting Tpe and Tpe response to exercise and to recovery loci were highly expressed. Due to the limited number of identified loci for Tpe response to exercise and to recovery, we used g:profiler^38^ to perform functional profiling of gene lists using various kinds of biological evidence (including GO, HPO annotation). Enrichment results with false discovery rate < 5% were deemed significant.

Furthermore, to systematically characterize the functional, cellular, and regulatory contribution of genetic variation, we used GARFIELD,^39^ analyzing the enrichment of genome-wide association summary statistics in tissue-specific functional elements at given significance thresholds.

The National Center for Biotechnology Information (NCBI) Gene database and GeneCards: The Human Gene Database were used to obtain official full names and, where relevant, common aliases for each candidate gene product. NCBI’s PubMed was used to interrogate primary literature pertaining to gene function. We also reviewed gene-specific animal models using International Mouse Phenotyping Consortium^40^ and the Mouse Genome Informatics database.

Finally, to explore shared mechanisms of disease, we assessed association of our identified SNVs (and their proxies, r^2^ ≥ 0.8) with other traits from published GWAS using PhenoScanner.^41^ Our group has recently performed GWASs on the PR interval (MIM: 108980)^42^ and on two traits related to the Tpe interval, T-wave morphology restitution during exercise, and during recovery,^43^ but results are not yet available in PhenoScanner or GWAS Catalog. In addition, a recent paper also still not in PhenoScanner or GWAS Catalog reported genetic variants significantly associated with the QRS complex.^44^ We, therefore, performed a lookup of the reported lead SNVs in our results to check for pleiotropy.

### Genetic Risk Score Analyses

To evaluate the impact of a genetically prolonged Tpe interval on ventricular arrhythmic risk (definition can be found in Table S2 and in the Supplemental Methods), we split all remaining individuals from UKB into training (n = 274,256, 0.6% arrhythmic events) and validation (n = 68,563, 0.6% arrhythmic events) subsets. These were unrelated UKB individuals of European ancestry not included in the EST-UKB and IMAGE-UKB cohorts, who passed genetic QC, were free of a previous history of CV events, and were unrelated (FULL-UKB, Figure S2). This split was random, but we ensured a similar prevalence of events across both subsets. We obtained the optimal p value cut-off for the GRS using PRSice v.2^45^ in the training subset (Supplemental Methods). We then applied logistic regression to test for an association between the GRS derived with the optimal p value cut-off and ventricular arrhythmic risk in the validation set.

## Results

The median (interquartile range) values of resting Tpe in both the EST-UKB and the IMAGE-UKB cohorts was 62 (12) ms (Figure S3). Histograms showing the distribution of the three traits are provided in Figure 2. The heritability estimate of resting Tpe was 15.6%, and its genetic correlations were 0.30 with Tpe response to exercise and 0.11 with Tpe response to recovery. The heritability estimates of the Tpe responses to exercise and to recovery were relatively low, 2.2% and 2.4%, respectively, and their genetic correlation to each other was 0.55.

### 28 Genetic Loci Are Associated with Resting Tpe

In the discovery GWAS for resting Tpe, 12 loci were genome-wide significant (p ≤ 5 × 10^−8^, Table S3). Using a significance threshold of p < 1 × 10^−6^, 21 variants (considering one lead SNV per 1 Mb region) were identified as significant and taken forward into replication in ∼42,000 independent samples from UKB. Of the 21 selected SNVs for resting Tpe, 15 formally replicated (p ≤ 0.05/21 = 2.4 × 10^−3^) and all had concordant directions of effect (Table 1).

**Table 1:** Discovery, Replication, and Full GWAS Results for the Lead SNVs for Resting Tpe Interval

						**Discovery**					**Replication**					**Combined**				
Locus	SNV	CHR	BP	EA	AA	EAF	β	SE	p	n	EAF	β	SE	p	n	EAF	β	SE	p	n
***RNF207***	**rs10864434**	**1**	**6262231**	**A**	**T**	**0.602**	**−0.066**	**0.008**	**1.20E−15**	**28,511**	**0.600**	**−0.054**	**0.007**	**2.50E−14**	**39,789**	**0.601**	**−0.058**	**0.005**	**5.80E−28**	**68,658**
***SSBP3***	**rs603901**	**1**	**54741767**	**C**	**T**	**0.434**	**−0.059**	**0.008**	**3.80E−13**	**29,111**	**0.436**	**−0.040**	**0.007**	**7.30E−09**	**40,626**	**0.435**	**−0.049**	**0.005**	**5.70E−21**	**70,103**
***SGIP1***	**rs10789207**	**1**	**66991346**	**T**	**C**	**0.787**	**−0.065**	**0.010**	**2.30E−11**	**29,383**	**0.784**	**−0.043**	**0.008**	**1.50E−07**	**41,005**	**0.786**	**−0.054**	**0.006**	**7.50E−18**	**70,757**
*KCND3*	rs116532272	1	112560237	G	A	0.986	0.133	0.036	1.90E**−**04	26,964	0.986	0.140	0.030	3.20E**−**06	37,629	0.986	0.140	0.023	9.10E**−**10	64,931
***MEF2D***	**rs1050316**	**1**	**156434703**	**G**	**T**	**0.345**	**−0.070**	**0.008**	**1.30E−16**	**29,481**	**0.349**	**−0.055**	**0.007**	**1.40E−14**	**41,142**	**0.347**	**−0.061**	**0.005**	**9.40E−30**	**70,993**
*DPT†*	rs607484	1	168687512	T	C	0.733	0.042	0.009	2.90E**−**06	29,624	0.734	0.024	0.008	1.90E**−**03	41,342	0.733	0.032	0.006	3.10E**−**08	71,338
***STRN***	**rs3770774**	**2**	**37192495**	**T**	**C**	**0.518**	**−0.040**	**0.008**	**7.90E−07**	**29,310**	**0.520**	**−0.029**	**0.007**	**2.30E−05**	**40,903**	**0.519**	**−0.032**	**0.005**	**8.20E−10**	**70,581**
*SLC8A1*	rs35450971	2	40754314	T	C	0.936	0.068	0.016	3.20E**−**05	29,231	0.935	0.056	0.014	4.60E**−**05	40,794	0.935	0.062	0.010	3.30E**−**09	70,392
*SERTAD2*	rs12466865	2	64882414	C	T	0.639	**−**0.029	0.008	6.00E**−**04	28,778	0.639	**−**0.042	0.007	4.80E**−**09	40,161	0.639	**−**0.037	0.005	7.70E**−**12	69,301
***SCN5A-SCN10A†***	**rs7373065**	**3**	**38710315**	**T**	**C**	**0.020**	**−0.149**	**0.030**	**5.70E−07**	**27,547**	**0.019**	**−0.143**	**0.026**	**2.60E−08**	**38,443**	**0.019**	**−0.140**	**0.019**	**3.80E−13**	**66,336**
***CAMK2D***	**rs35132791**	**4**	**114456506**	**C**	**G**	**0.744**	**−0.053**	**0.009**	**8.90E−09**	**29,455**	**0.740**	**−0.025**	**0.008**	**9.90E−04**	**41,107**	**0.742**	**−0.037**	**0.006**	**3.10E−10**	**70,932**
*NKX2-5*	rs6882776	5	172664163	G	A	0.720	**−**0.039	0.009	1.10E**−**05	29,157	0.716	**−**0.050	0.008	5.70E**−**11	40,690	0.717	**−**0.044	0.006	1.50E**−**14	70,213
*RUFY1*	rs80090179	5	178936268	T	G	0.989	**−**0.170	0.040	2.10E**−**05	27,892	0.989	**−**0.144	0.034	2.00E**−**05	38,925	0.989	**−**0.156	0.026	9.50E**−**10	67,167
***SLC35F1***	**rs12210810**	**6**	**118653204**	**G**	**C**	**0.944**	**0.106**	**0.018**	**1.30E−09**	**29,624**	**0.945**	**0.123**	**0.015**	**1.20E−16**	**41,342**	**0.945**	**0.118**	**0.011**	**4.60E−26**	**71,338**
***CREB5***	**rs12700888**	**7**	**28409532**	**A**	**C**	**0.264**	**0.047**	**0.009**	**2.00E−07**	**29,355**	**0.261**	**0.040**	**0.008**	**2.30E−07**	**40,967**	**0.262**	**0.042**	**0.006**	**5.80E−13**	**70,691**
*CAV2*	rs17138749	7	116133098	A	C	0.838	**−**0.036	0.011	7.50E**−**04	29,511	0.837	**−**0.042	0.009	6.00E**−**06	41,184	0.838	**−**0.040	0.007	9.30E**−**09	71,065
***KCNH2***	**rs113843864**	**7**	**150618509**	**G**	**A**	**0.752**	**0.077**	**0.009**	**6.70E−17**	**29,582**	**0.752**	**0.047**	**0.008**	**2.60E−09**	**41,283**	**0.752**	**0.059**	**0.006**	**4.10E−23**	**71,236**
***PRAG1***	**rs2976944**	**8**	**8270914**	**T**	**C**	**0.486**	**−0.040**	**0.008**	**8.10E−07**	**29,199**	**0.489**	**−0.025**	**0.007**	**2.60E−04**	**40,749**	**0.488**	**−0.028**	**0.005**	**3.50E−08**	**70,315**
*MSRA*	rs10283145	8	10241411	C	T	0.484	0.027	0.008	8.20E**−**04	29,386	0.482	0.034	0.007	4.80E**−**07	41,009	0.483	0.030	0.005	5.50E**−**09	70,764
*AZIN1*	rs608236	8	103928940	A	G	0.433	0.030	0.008	2.70E**−**04	29,264	0.433	0.044	0.007	1.30E**−**10	40,839	0.433	0.038	0.005	2.80E**−**13	70,471
*ZMIZ1*	rs2486695	10	80871063	G	A	0.612	**−**0.038	0.008	3.50E**−**06	29,466	0.615	**−**0.038	0.007	4.70E**−**08	41,121	0.613	**−**0.037	0.005	1.70E**−**12	70,957
***IGF1R***	**rs2871974**	**15**	**99284074**	**C**	**T**	**0.363**	**−0.054**	**0.008**	**1.00E−10**	**29,500**	**0.358**	**−0.032**	**0.007**	**5.40E−06**	**41,169**	**0.360**	**−0.042**	**0.005**	**2.90E−15**	**71,040**
*LITAF†*	rs2080512	16	11692198	G	T	0.538	**−**0.035	0.008	1.60E**−**05	29,452	0.539	**−**0.031	0.007	3.40E**−**06	41,102	0.538	**−**0.034	0.005	2.00E**−**11	70,924
*GINS3*	rs1424077	16	58462627	G	A	0.273	**−**0.050	0.009	1.70E**−**08	29,486	0.275	**−**0.020	0.008	7.50E**−**03	41,150	0.274	**−**0.033	0.006	5.60E**−**09	71,006
***KCNJ2^∗^***	**rs4399570**	**17**	**68479345**	**G**	**A**	**0.698**	**0.162**	**0.009**	**2.20E−78**	**29,508**	**0.699**	**0.125**	**0.007**	**1.60E−64**	**41,180**	**0.699**	**0.142**	**0.006**	**5.30E−143**	**71,058**
***PYGB***	**rs55769542**	**20**	**25272895**	**C**	**CA**	**0.674**	**−0.047**	**0.009**	**3.70E−07**	**25,533**	**0.674**	**−0.030**	**0.008**	**8.30E−05**	**35,633**	**0.674**	**−0.037**	**0.006**	**2.60E−10**	**61,487**
*DEFB118*	rs36094783	20	29934214	G	A	0.932	**−**0.068	0.016	2.50E**−**05	28,334	0.932	**−**0.050	0.014	2.70E**−**04	39,541	0.932	**−**0.057	0.010	3.20E**−**08	68,231
***KCNJ4***	**rs196064**	**22**	**38851392**	**C**	**T**	**0.632**	**0.049**	**0.008**	**3.80E−09**	**29,421**	**0.634**	**0.049**	**0.007**	**3.00E−12**	**41,058**	**0.633**	**0.049**	**0.005**	**1.30E−20**	**70,848**

Abbreviations: SNV, single-nucleotide variation; CHR, chromosome; BP, position, based on HG build 19; EA, effect allele; AA, alternate allele; EAF, effect allele frequency from discovery data; β, beta; SE, standard error; n, number of participants; p, p value. The locus name indicates the gene that is in the closest proximity to the most associated SNV. Replicated SNVs in the replication cohort are indicated in bold type.

^a^Indicates has a secondary signal. The secondary signal at DPT, rs761499672, was located 379 kb away from the lead SNV, rs607484, while the secondary signals at SCN5A-SCN10A, rs6797133 and rs6801957, were located 54 kb and 57 kb, respectively, away from the lead SNV (rs7373065); and the secondary signal at LITAF, rs570620219, was located 40 kb away from the lead SNV (rs2080512).

^b^Lead SNV is in moderate LD (r^2^ = 0.56) with the lead SNV for Tpe response to exercise.

Thirteen additional SNVs (also considering one lead SNV per 1 Mb region) reached genome-wide significance in the full dataset GWAS, all with concordant directions of effect across discovery, validation, and full cohort datasets (Table 1). Manhattan plots in the full dataset GWAS results are shown in Figure S4A, and QQ plots including the discovery (blue) and full dataset (black) GWASs are shown in Figure S5. The intercept of the LD Score Regression^31^ was 1.005 (standard error of 0.0097), indicating inflation of the lambdas is predominantly due to polygenicity and not to underlying QC factors or population stratification. Regional plots are shown in Figure S6.

Conditional analyses showed evidence for four secondary independent signals at loci *DPT* (MIM: 125597), *SCN5A-SCN10A* (MIM: 600163), and *LITAF* (MIM: 603795, Table 1, Figure S7). The secondary signal at *DPT*, rs761499672, was located 379 kb away from the lead SNV, rs607484; while the secondary signals at *SCN5A-SCN10A*, rs6797133 and rs6801957, were located 54 kb and 57 kb, respectively, away from the lead SNV (rs7373065). Finally, the secondary signal at *LITAF*, rs570620219, was located 40 kb away from the lead SNV (rs2080512).

Taken together, across both the replication stage and full dataset GWAS, we identified 32 SNVs (28 lead SNVs + 4 secondary SNVs) in 28 loci for resting Tpe (Figure 3; Table 1), which explained 3.20% of its variance. This corresponds to ∼21% of its estimated heritability.

### Three Genetic Loci Are Associated with the Tpe Response to Exercise and Recovery

For Tpe response to exercise and to recovery traits, no genome-wide significant loci were found in the discovery cohorts. Four and seven variants for each Tpe response trait, respectively, met our pre-defined threshold of p < 10^−6^ to take forward into replication in ∼22,000 independent samples. One of the selected SNVs for Tpe response to exercise formally replicated (p ≤ 0.05/4 = 0.0125) and had concordant directions of effect in discovery and replication datasets (Table 2). None of the seven SNVs for Tpe response to recovery that were taken forward into replication formally replicated (Table 3).

**Table 2:** Discovery, Replication, and Full GWAS Results for the Lead SNVs for Tpe Dynamics during Exercise

						**Discovery**					**Replication**					**Combined**				
**Locus**	**SNV**	**CHR**	**BP**	**EA**	**AA**	**EAF**	**β**	**SE**	**p**	**n**	**EAF**	**β**	**SE**	**p**	**n**	**EAF**	**β**	**SE**	**p**	**n**
*EIPR1*	rs11127417	2	3357993	G	T	0.012	0.154	0.038	4.30E−05	29,389	0.011	0.165	0.045	2.30E−04	21,854	0.012	0.161	0.029	2.40E−08	51,764
***KCNJ2^∗^***	**rs1468572**	**17**	**68411445**	**T**	**C**	**0.781**	**0.051**	**0.010**	**2.20E**−**07**	**29,010**	**0.781**	**0.048**	**0.012**	**2.70E**−**05**	**21,572**	**0.780**	**0.050**	**0.007**	**2.70E**−**11**	**51,096**

Abbreviations: SNV, single-nucleotide variation; CHR, chromosome; BP, position, based on HG build 19; EA, effect allele; AA, alternate allele; EAF, effect allele frequency from discovery data; β, beta; SE, standard error; n, number of participants; p, p value. The locus name indicates the gene that is in the closest proximity to the most associated SNV. Replicated SNV is indicated in bold type.

^a^Lead SNV is in moderate LD (r^2^ = 0.56) with the lead SNV for resting Tpe.

**Table 3:** Discovery, Replication, and Full GWAS Results for the Lead SNV for Tpe Dynamics during Recovery

						**Discovery**					**Replication**					**Combined**				
**Locus**	**SNV**	**CHR**	**BP**	**EA**	**AA**	**EAF**	**β**	**SE**	**p**	**n**	**EAF**	**β**	**SE**	**p**	**n**	**EAF**	**β**	**SE**	**p**	**n**
*NAF1*	rs150100144	4	163978319	G	AA	0.962	0.109	0.024	3.40E−06	23,300	0.961	0.088	0.031	4.49E−03	17,206	0.962	0.101	0.018	1.10E−08	41,090

Abbreviations: SNV, single-nucleotide variation; CHR, chromosome; BP, position, based on HG build 19; EA, effect allele; AA, alternate allele; EAF, effect allele frequency from discovery data; β, beta; SE, standard error; n, number of participants; p, p value. The locus name indicates the gene that is in the closest proximity to the most associated SNV.

One additional SNV reached genome-wide significance in the full dataset GWAS for each Tpe response trait, all with concordant directions of effect across discovery and replication datasets (Tables 2 and 3). Manhattan plots for the full dataset GWAS results are shown in Figures S4B and S4C, and QQ plots including the discovery (blue) and full dataset (black) GWASs are shown in Figure S5, where the value of the lambdas suggests there was minimal inflation. Regional plots are shown in Figures S8 and S9. We performed conditional analyses and no independent signals were found at any of the identified loci.

**Figure 4:**
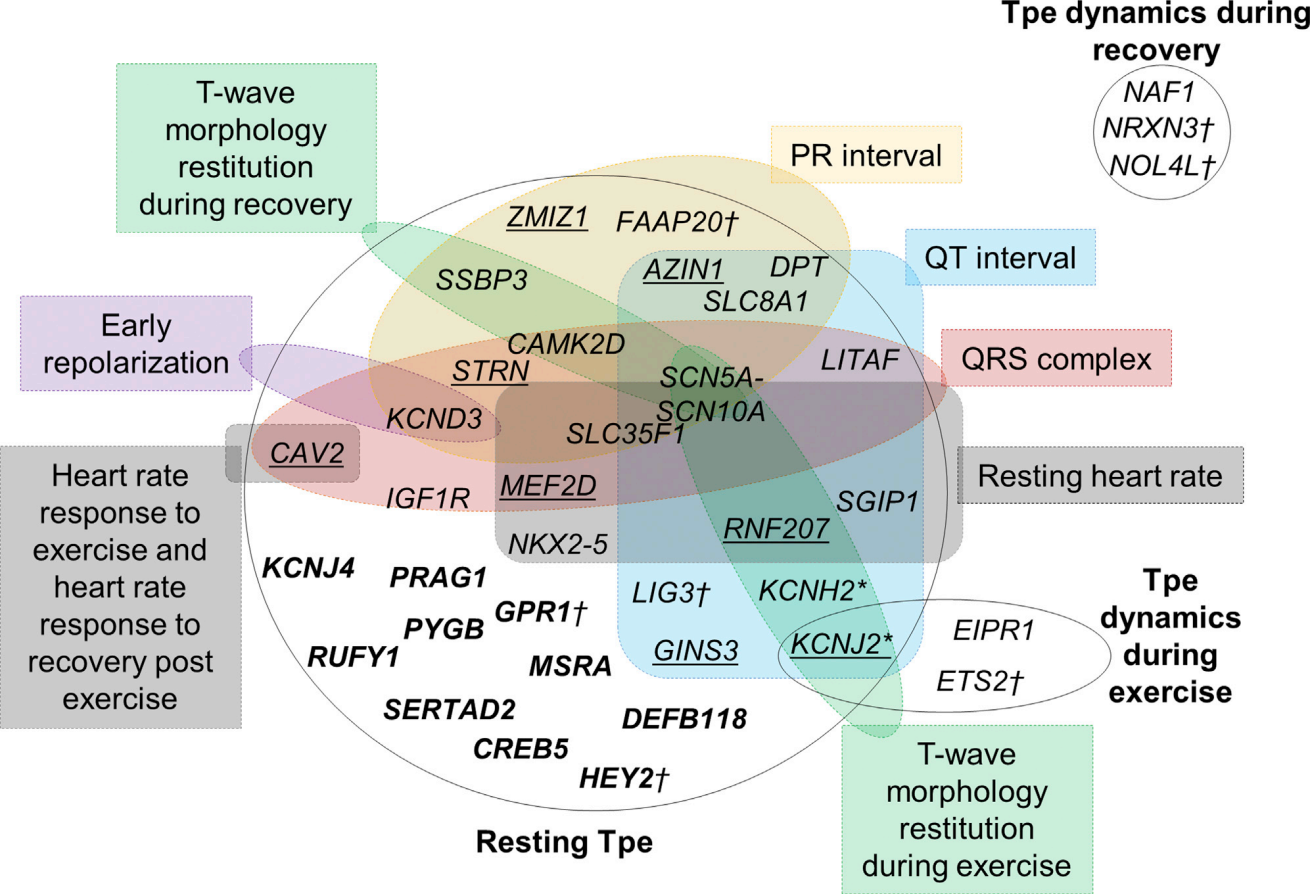
Overlap of Resting Tpe, Tpe Response to Exercise, and Tpe Response to Recovery Loci with Other Electrocardiogram Traits SNVs at loci with a known genome-wide significant association (from PhenoScanner or GWAS catalog) with other ECG traits are grouped accordingly. The locus names indicate the nearest coding genes. The *KCNJ2* locus was shared between resting Tpe and Tpe dynamics during exercise. There was no loci overlap between Tpe response to recovery and resting Tpe or Tpe response to exercise. There was a substantial number of loci for resting Tpe that did not overlap with other ECG traits. Underlined loci are loci that have previously been associated with other ECG markers but the reported variant was not in high LD (r^2^ < 0.8) with our lead variant, so potentially independent signals at those loci. Bold loci are loci that have not been associated with other ECG marker. *†*Indicates sex-specific loci. ^∗^Indicates previously associated with Tpe interval in other studies (PMID: 20215044 and 22342860).

In total, across both the replication stage and full dataset GWAS, we identified two loci for Tpe response to exercise, which explained 0.16% of its variance, and one for Tpe response to recovery, which explained 0.06% of its variance (Figure 3 and Tables 2 and 3). Of note, the one locus identified for Tpe response to recovery did not overlap with resting Tpe interval or Tpe response to exercise (Figure 4).

### Four Male-Specific Loci for Resting Tpe and Three for Tpe Response to Exercise and Recovery

We identified variants associated with resting Tpe in males at four additional loci: *FAAP20* (MIM: 615183), *GPR1* (MIM: 600239), *HEY2* (MIM: 604674), and *LIG3* (MIM: 600940). Variants at these loci were not significant (p > 5 × 10^−8^) in the combined analyses (Table S4A, Figure S10). For Tpe response to exercise, we identified one variant at the *ETS2* locus (MIM: 164740) for males (n = 24,241). This variant was non-significant in the combined sex GWAS (Table S4B, Figure S11). Finally, for Tpe response to recovery, we identified two female-specific variants at loci *NRXN3* (MIM: 600567) and *NOL4L* (Table S4C, Figure S12).

### Bioinformatics for Resting Tpe Loci

None of the lead variants or their close proxies (r^2^ > 0.8) for resting Tpe were annotated as missense variants. However, we identified regulatory variants that might affect gene expression levels of their target genes in heart and brain tissue by interrogating publicly available eQTL datasets using GTEx (see Web Resources). Nine lead variants associated with resting Tpe (at *SSBP3* [MIM: 607390], *SGIP1* [MIM: 611540], *NKX2-5* [MIM: 610610], and *LIG3*) were in high LD (r^2^ > 0.8) with top eQTL variants in cardiac and brain tissue (Table S5). We found strong support for pairwise colocalization of SNVs in GWAS with eQTLs at five genes (*SSBP3*, *SGIP1, IGF1R, LITAF*, and *LIG3*) in cardiac left ventricle, three genes (*SSBP3*, *NDRG4* [MIM: 614463], and *LIG3*) in cardiac atrial appendages and three genes (*NKX2-5, RP11-481J2.2*, and *LIG3*) in brain tissue (Table S5).

We next identified 34 potential target genes at 15 resting Tpe loci whose promoter regions form significant chromatin interactions in brain and heart using publicly available Hi-C data (Table S6A).

These results were used to prepare a list of potential candidate genes for each identified locus for resting Tpe (Table S7A).

### Enriched Tissues, Gene Sets, and Pathways for Resting Tpe Loci

We observed a significant enrichment of resting Tpe loci in heart tissue (Figure S13). By considering all identified loci, our DEPICT analyses identified enrichment of expression in the heart, in the ventricles and in the atria, with the greatest enrichment in the heart (p = 1.87 × 10^−4^, false discovery rate < 0.01, Table S8). We also observed significant enrichments (a false discovery rate < 0.05) in 17 gene sets from the Gene Ontology, 15 from the Mouse Phenotype Ontology, 54 from ENSEMBL, and 3 from Kyoto encyclopedia of genes and genomes. The most significant enrichments were negative regulation of transport (p = 2.26 × 10^−6^) from the Gene Ontology, increased infarction size (p = 4.04 × 10^−6^) from the Mouse Phenotype Ontology, the NOS3 PPI subnetwork (p = 2.62 × 10^−9^) from ENSEMBL, and regulation of actin cytoskeleton (p = 1 × 10^−4^) from Kyoto encyclopedia of genes and genomes (Tables S9A–S9D).

### Bioinformatics Analyses of the Tpe Response to Exercise and Recovery Loci

None of the lead variants for Tpe response to exercise or to recovery or their close proxies (r^2^ > 0.8) were annotated as missense variants or were identified as regulatory variants that might affect gene expression levels of their target genes in heart and brain tissue.

We identified the genes *ETS2* for Tpe response to exercise and *KIK3B* (MIM: 603754) for Tpe response to recovery whose promoter regions formed significant chromatin interactions with them in the left ventricle (Tables S6B and S6C). There were no significant results from DEPICT analyses, so we performed pathway analyses using g:profiler^38^ including only nearest genes or candidate genes indicated from long-range interaction results (Tables S7B and S7C). The top enriched pathways for Tpe response to exercise were regulation of skeletal muscle contraction by action potential (p = 3.77 × 10^−2^) and regulation of skeletal muscle contraction via regulation of action potential (p = 3.77 × 10^−2^, Figure S14). We did not observe any significant biological process for the candidate genes for Tpe response to recovery.

### Association of Resting Tpe and Tpe Response to Exercise and to Recovery Loci with Other Traits

SNVs at 13 loci for resting Tpe had previously been associated (p < 5 × 10^−8^) with other traits, including pulse rate, P-wave duration, resting heart rate (MIM: 607276), QT interval, QRS duration, cardiomegaly, Brugada syndrome (MIM: 601144), and atrial fibrillation (MIM: 608583, Table S10). Variants at two loci (*SSBP3* and *DPT*) for the PR interval, at four loci (*KCND3* [MIM: 605411], *MEF2D* [MIM: 600663], *CAMK2D* [MIM: 607708], and *LITAF*) and at five loci (*SSBP3*, *SCN5A-SCN10A*, *CAMK2D*, *KCNH2* [MIM: 152427], and *KCNJ2* [MIM: 600681]) for the T-wave morphology restitution were genome-wide significant in our results (Table S11).

An overview of loci for resting Tpe and Tpe response to exercise and to recovery with other ECG traits is indicated by a Venn diagram in Figure 4. Interestingly, the loci for Tpe response to exercise and to recovery did not overlap with other ECG traits, except for the *KCNJ2* locus associated with Tpe response to exercise. This locus has also been associated with resting Tpe. It should be noted, however, that both lead SNVs were not in high LD (r^2^ = 0.56, Tables 1 and 2).

### Genetic Risk Score for Resting Tpe

The optimal p value cut-off in the training set was p = 0.012 (Figure S15, 12,107 SNVs). The GRS was not significantly associated with arrhythmic events in the validation subset (p = 0.13, Figure S15).

## Discussion

This is the largest study to date studying the genetic contribution to the Tpe interval, and Tpe response to exercise and recovery. With the unique combination of a robust framework, including independent discovery and replication samples, and dense genetic imputation in ∼72,000 individuals,^25^ we identified 28 loci and 4 male-specific loci for resting Tpe, 10 of which are specific to resting Tpe. We also identified three loci for Tpe response to exercise (one male-specific locus) and three loci for Tpe response to recovery (two female-specific loci). One locus (*KCNJ2*) for the Tpe response to exercise had previously been associated with other ECG traits including resting Tpe. The main biological processes indicated for resting Tpe involved ventricular repolarization and cardiac conduction and contraction.

Of the total 32 loci discovered in this work, 10 (4 validated, 4 identified in the full dataset GWAS, and 2 male-specific) did not overlap with any locus previously reported for another ECG trait (*PRAG1* [MIM: 617344], *PYGB* [MIM: 138550], *CREB5* [MIM: 618262], *KCNJ4* [MIM: 600504], *MSRA*, *RUFY1* [MIM: 610327], *SERTAD2* [MIM: 617851], *DEFB118* [MIM: 607650], *GPR1*, and *HEY2*, respectively; Figure 4). Of the remaining loci, 12 (11 lead and 1 male-specific) were associated with resting QT interval. Two additional loci were associated with resting heart rate, five with QRS complex and three (two lead and one male-specific) with PR interval (Figure 4). These observations underline, as expected, shared genetics among ECG traits, but importantly we also observed specific Tpe loci.

Of the ten resting Tpe-specific loci, a summation of bioinformatics analyses and literature review indicated eight loci (*PRAG1*, *PYGB*, *KCNJ4*, *MSRA*, *RUFY1*, *SERTAD2*, *GPR1*, and *HEY2*) had plausible candidate genes (*PPP1R3B/MFHAS1* [MIM: 610541/605352], *PYGB*, *KCNJ4, GATA4* [MIM: 600576], *RUFY1*, *SERTAD2*, *GPR1/ZDBF2* [MIM: 617059], and *HEY2*; Table S7A). From the candidate genes at validated loci, *PYGB* encodes a glycogen phosphorylase (GP) that is found in the heart. The physiological role of myocardial GP is to provide the energy supply required for myocardial contraction and it is associated with diseases including myocardial infarction (MIM: 608446).^46^ A second candidate gene, *KCNJ4*, functions closely with *KCNJ2* (also identified in this work). Both genes encode the human inward rectifier potassium channels Kir2.1 and Kir2.3. These potassium selective ion channels determine the resting membrane potential and terminal repolarization of the cardiac action potential. Importantly, mutations in *KCNJ4* are associated with electrolyte imbalance and dilated cardiomyopathy (MIM: 115200).^47^ From the candidate genes identified from the full dataset GWAS, *GATA4*, a candidate gene at locus *MSRA*, plays a key role in cardiac development and function.^48^ In co-operation with *TBX5* (MIM: 601620), it binds to cardiac super-enhancers and promotes cardiomyocyte gene expression, while it downregulates endocardial and endothelial gene expression.^48^ Mutations in this gene have been associated with cardiac septal defects,^49^^,^^50^ tetralogy of Fallot (MIM: 187500),^51^ cardiac myocyte enlargement,^52^ and atrial fibrillation.^53^ Finally, from the candidate genes identified in sex-specific analyses, *HEY2* encodes a member of the hairy and enhancer of split-related family of transcription factors. Two similar and redundant genes in the mouse are required for embryonic cardiovascular development. Interestingly, the lead variant we have identified at this locus, rs10457469, is in high LD (r^2^ = 0.97) with rs9388451, which has been reported to be associated with Brugada syndrome through a *HEY2*-dependent alteration of ion channel expression across the cardiac ventricular wall.^54^ Interestingly, *HEY2* represses transcription by the cardiac transcriptional activators *GATA4* and *GATA6* (MIM: 601656).^55^

Bioinformatics analyses on all loci identified in this study indicate that the main biological mechanism underlying resting Tpe is predominantly driven by cellular processes that control ventricular repolarization. As highlighted above, *KCNJ2* and *KCNJ4* are resting Tpe-specific candidate genes involved in ventricular repolarization. In particular, the SNV rs4399570, mapping to *KCNJ2,* demonstrated the strongest association with resting Tpe (p = 5.30 × 10^−143^) and has one of the largest effect sizes for this trait (1.30 ms). Mutations in *KCNJ2* are associated with short QT syndrome 3 (MIM: 609622)^56^ and cardiac arrhythmias.^57^^,^^58^ In addition, we identified variants at *KCNH2* and *RNF207* (MIM: 616923), both loci previously associated with the QT interval.^59^ *KCNH2* is another important gene that encodes a crucial potassium repolarizing current, *HERG*. Finally, *RNF207*, a RING finger protein, is a known modulator of cardiac repolarization through actions on *HERG*.^60^ These four loci were validated in our work.

An additional biological mechanism underlying resting Tpe is cardiac conduction and contraction. Several candidate genes, such as *PYGB*, *GATA4*, and *HEY2* (highlighted before), as well as previously reported *SCN5A-SCN10A, CAMK2D*, and *KCND3* are involved. *CAMK2D* is the candidate gene at the validated locus *CAMK2D* and is a calcium/calmodulin-dependent protein kinase involved in the excitation-contraction coupling in heart by targeting Ca(2+) influx into the myocyte. *KCND3* is the candidate gene at locus *KCND3*, discovered in the full dataset GWAS, and encodes the Ito carrying KV4.3 channel, and gain-of-function mutations have been associated with Brugada syndrome^61^ and atrial fibrillation.^62^ Recent studies have suggested that an increased KV4.3 expression modulates NaV1.5 sodium current, resulting in a loss of conduction.^63^ A possible biological mechanism linking ventricular repolarization and cardiac contraction is cardiac mechano-electric coupling, by which myocardial deformation causes changes in cardiac electrophysiological parameters^64^^,^^65^ and mechanosensitive ion channels modulate ventricular repolarization during ventricular contraction.

Our work significantly expands previous literature on the genetic architecture of the Tpe interval. A previous study^21^ on this topic examined the relationship of seven SNVs previously associated with the QT interval to the Tpe interval in 5,890 individuals, two SNVs at *KCNH2* were genome-wide significant in our results (Table S1). The second study^22^ performed a GWAS for resting Tpe interval on 1,870 individuals. They discovered and validated a strong signal (p = 1.1 × 10^−10^), at *KCNJ2*, a locus that was also highly significant in our results (p = 4.2 × 10^−148^). However, their reported suggestive SNV, rs17749681, at *GRIN2A* (MIM: 138253), was non-significant in our results (Table S1).

The identified loci for Tpe response to exercise and Tpe response to recovery are potentially interesting as there was almost no overlap between traits, with resting Tpe or with other ECG traits. To highlight one of the candidate genes for Tpe response to exercise, *ETS2*, mapping the male-specific locus *ETS2*, plays an important role in a genetic network that governs cardiopoiesis.^66^ It has been shown that variations in *ETS2* abundance in hearts of adult rodents and the associated loss of cardiomyocytes contribute to the longevity variability observed during normal aging of rats through activation of programmed necrosis.^67^ In the development of a functional myocardium and formation of the coronary vasculature, epicardially derived cells play an essential role, and *ETS2* was found to be essential for normal coronary and myocardial development in chicken embryos.^68^

In this study, the number of identified SNVs for Tpe response to exercise and to recovery was limited, and this might be partly due to the low heritability of the traits (2.2% for Tpe response during exercise and 2.4% for Tpe response during recovery). Our data suggest there is a significant genetic contribution to resting Tpe, but its response to heart rate changes is mainly influenced by environmental factors. This is a general feature that is emerging from our studies, namely that the heritability of exercise-induced changes in cardiac electrophysiology is lower than those at rest.^43^^,^^69^ Interventions such as exercise training may therefore have an impact on ventricular repolarization and, thus, reduce its associated risk.

Our sex-specific findings strengthen previous studies concluding that there are sex differences in the resting Tpe and its response to heart rate.^13^^,^^70^, ^71^, ^72^ Therefore, genetics might be playing a role in the modulation of cardiac electrical activity in addition to sex hormones, with men having a greater genetic influence compared to women.

Despite finding a significant association between the GRS and ventricular arrhythmic events in a training cohort, this significance was not validated in an independent subset of individuals. This might indicate that the common variants modulating resting Tpe do not contribute to the pathophysiological mechanisms influencing ventricular arrhythmic risk. Alternatively, given the low incidence of arrhythmic events in the UK Biobank, which comprises a relatively healthy population, the validation analysis might have been underpowered. Future studies should evaluate the prognostic value of the GRS in well-powered cohorts for validation of our negative results.

Our study has some limitations. First, we report results from GWASs including all available samples, which indicate seven loci for resting Tpe, two for Tpe response to exercise, and three loci for Tpe response to recovery with no independent replication, so these loci should be considered as preliminary until they are externally validated. Next, due to the relatively low sample size, we restricted our analysis to common variants (MAF > 1%), so we are unable to comment on the role of rare variants on the Tpe traits. In addition, we only report results for European ancestry as this was by far the largest ancestral group in the UKB cohort. Additional studies will need to investigate whether the findings can be extrapolated to other ancestries. Finally, the range of variation of the Tpe response to exercise and recovery traits is small, and the limited sampling rate of the ECG recordings (500 Hz), corresponding to a temporal resolution of 2 ms, might have hindered the resolution of these measurements.

In summary, our findings provide additional loci for Tpe interval traits and reveal the role of ventricular repolarization and cardiac conduction and contraction in modulating them. Our work may guide future studies identifying new therapeutic targets to modulate resting Tpe and its dynamics to prevent and treat ventricular arrhythmias.

## Declaration of Interests

The authors declare no competing interests.

## Web Resources

1000 Genomes reference panel, https://www.internationalgenome.org/category/reference/
Baseline wonder removal algorithm, https://github.com/Tereshchenkolab/S-ICD_eligibility
GTEx Portal, https://gtexportal.org/home/
GWAS Catalog, https://www.ebi.ac.uk/gwas/
Haplotype Reference Consortium, http://www.haplotype-reference-consortium.org/site
OMIM, https://www.omim.org/
PhenoScanner, http://www.phenoscanner.medschl.cam.ac.uk/
UK Biobank, https://www.ukbiobank.ac.uk

## Supplemental Data

Document S1. Supplemental Methods, Figures S1–S15, and Tables S1, S2, S4, S5, and S8

Table S3Genome-wide Significant SNVs for the Resting Tpe Interval in the Discovery Sample

Table S6 Long-Range Interactors in Heart and Neural Progenitor Cells

Table S7 Candidate Genes

Table S9 DEPICT Gene Set Enrichment across All Resting Tpe Loci

Table S10 Resting Tpe Loci Associations with Other Traits using PhenoScanner

Table S11 Resting Tpe Loci Associations with T-wave Morphology Restitution, and PR Interval and QRS Complex Traits

Document S2. Article plus Supplemental Information
