# Supplementary material for: Common Genetic Variants Modulate the Electrocardiographic Tpeak-to-Tend Interval
Source: Am J Hum Genet. 2020 May 7;106(6):764–78. doi: 10.1016/j.ajhg.2020.04.009 (PMC7273524; doi:10.1016/j.ajhg.2020.04.009)
Supplement: Document S1. Supplemental Methods, Figures S1–S15, and Tables S1, S2, S4, S5, and S8 [file mmc1.pdf]

The American Journal of Human Genetics, Volume *106*

Supplemental Data

Common Genetic Variants Modulate the Electrocardiographic Tpeak-to-Tend Interval

Julia Ramírez, Stefan van Duijvenboden, William J. Young, Michele Orini, Pier D. Lambiase, Patricia B. Munroe, and Andrew Tinker

Supplemental Figures

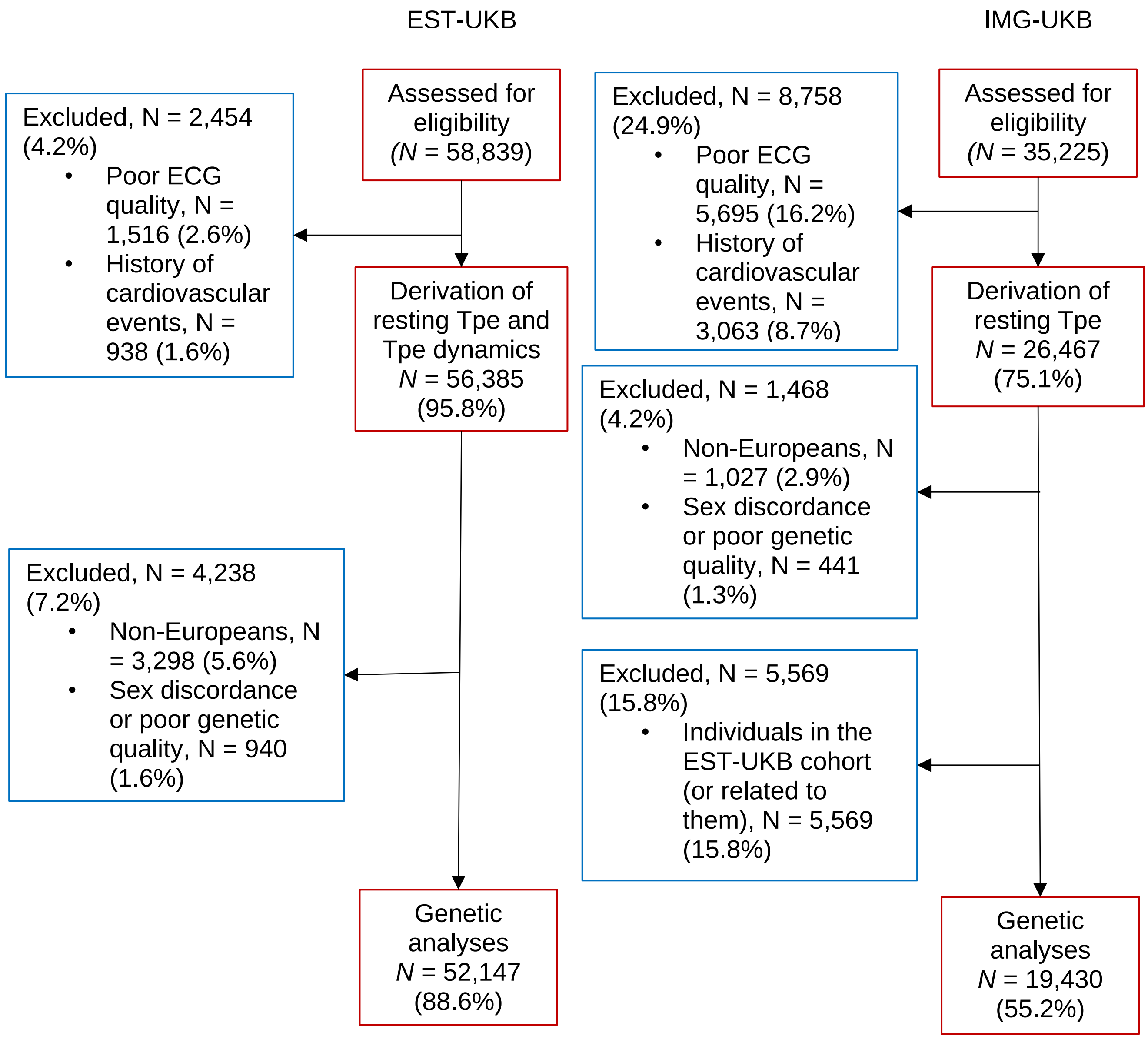

Figure S1: Exercise stress test (EST-UKB) and Imaging (IMG-UKB) study populations flow diagram.

Additional information can be found in Methods.

FULL-UKB cohort

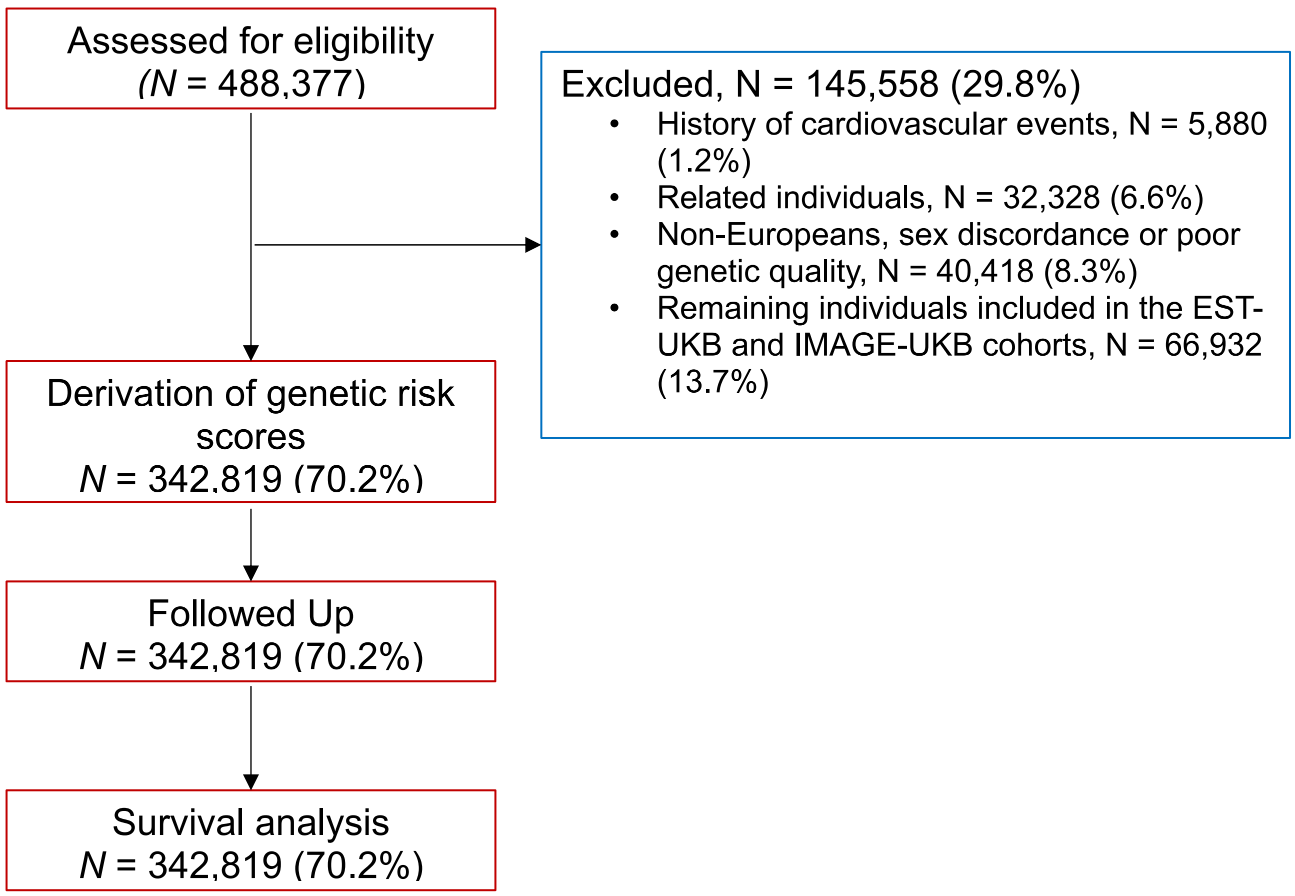

Figure S2: Full cohort (FULL-UKB) study population flow diagram.

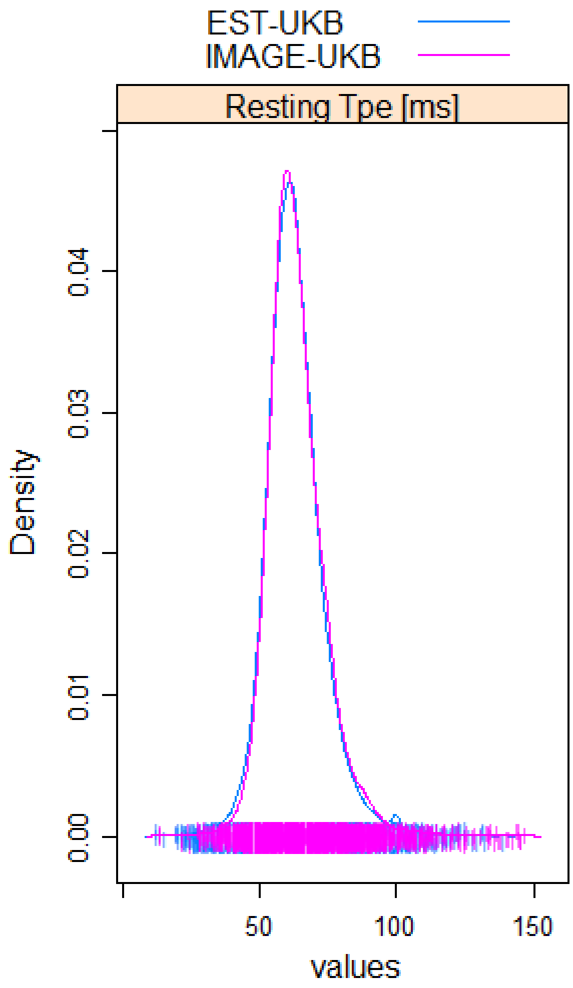

Figure S3: Density plots of resting Tpe intervals in the EST-UKB (blue) and IMAGE-UKB (pink) cohorts.

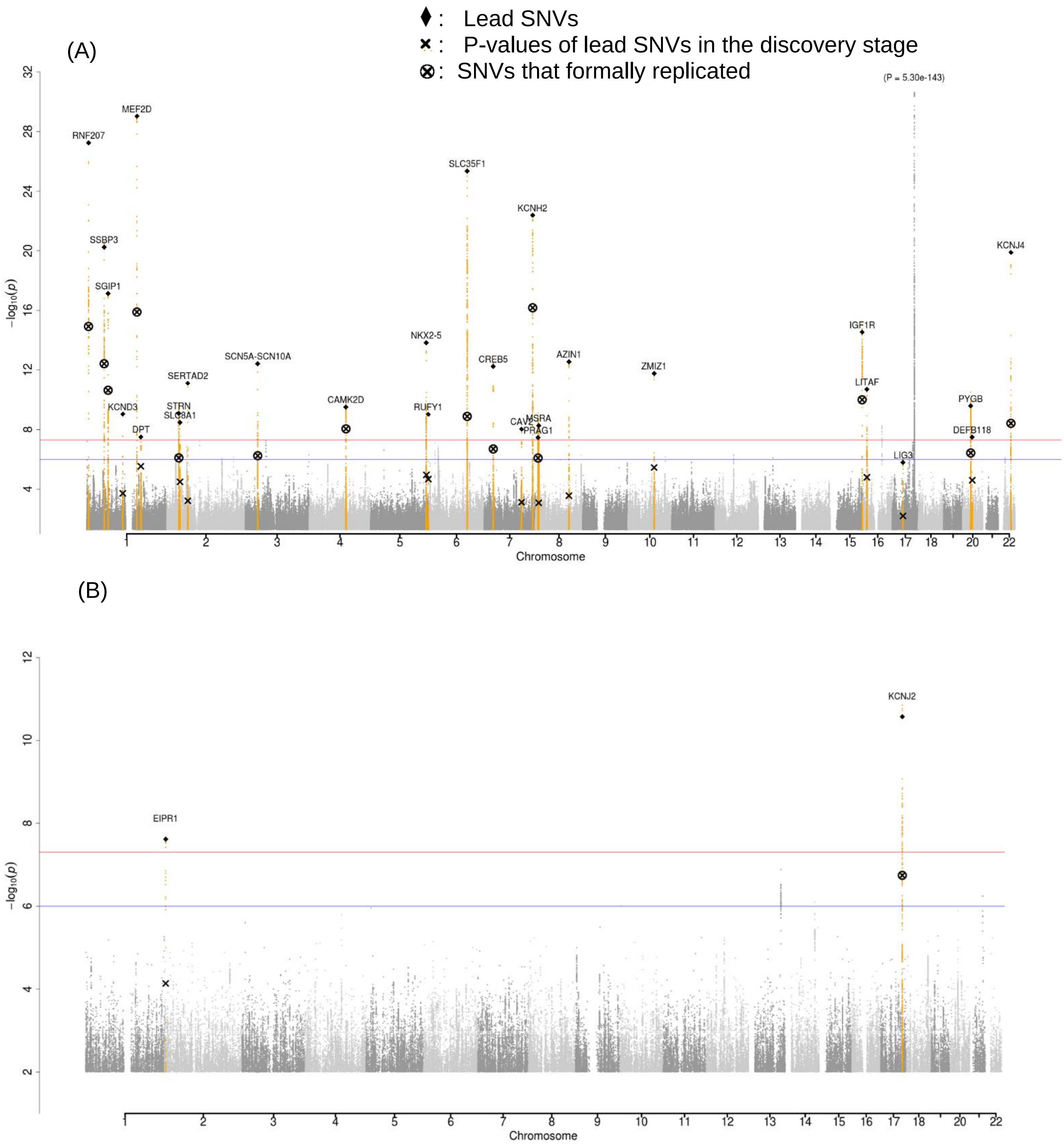
: Lead SNVs
: P-values of lead SNVs in the discovery stage
: SNVs that formally replicated
(A)
(P = 5.30e-143)
MEF2D
RNF207
SLC35F1
KCNH2
SSBP3
KCNJ4
SGIP1
IGF1R
NKX2-5
SCN5A-SCN10A
AZIN1
CREB5
ZMIZ1
SERTAD2
LITAF
CAMK2D
PYGB
KCND3
STRN
RUFY1
SLC8A1
CAV2
HSRA
DPT
PRAG1
DEFB118
LIG3
$-\log_{10}(p)$
Chromosome
(B)
KCNJ2
EIPR1
$-\log_{10}(p)$
Chromosome

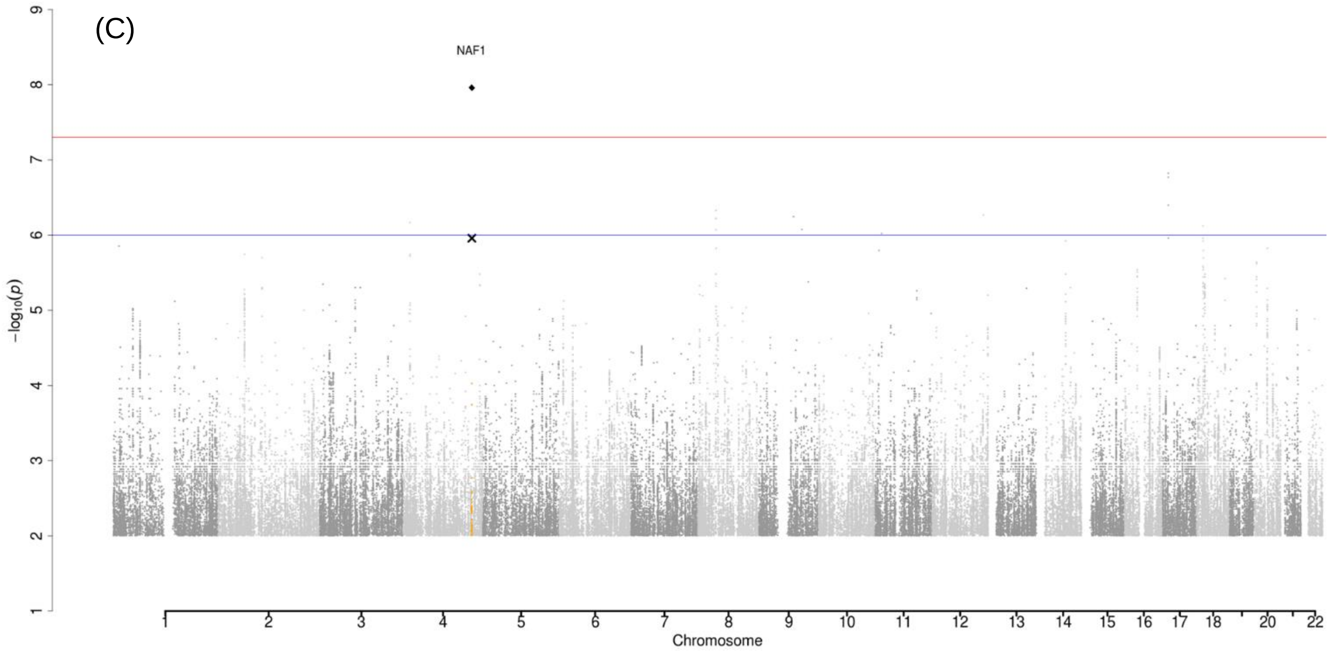

Figure S4: Manhattan plots of Tpe interval (A), Tpe dynamics during exercise (B) and during recovery (C) in the full cohort analysis.

P values, expressed as –log10(*P*), are plotted according to physical genomic locations by chromosome. Lead SNVs are marked by the diamonds. The crosses indicate the *P* values of these SNVs in the discovery data set. Crosses are encircled for SNVs that formally replicated. Locus names of the novel loci correspond to the nearest annotated gene. The blue horizontal line indicates a *P* value threshold of 1×10^{-6}, corresponding to the lookup significance threshold. The red horizontal line indicates a *P*-value threshold of 5×10^{-8}, corresponding to genome-wide significance. Novel loci are highlighted in yellow.

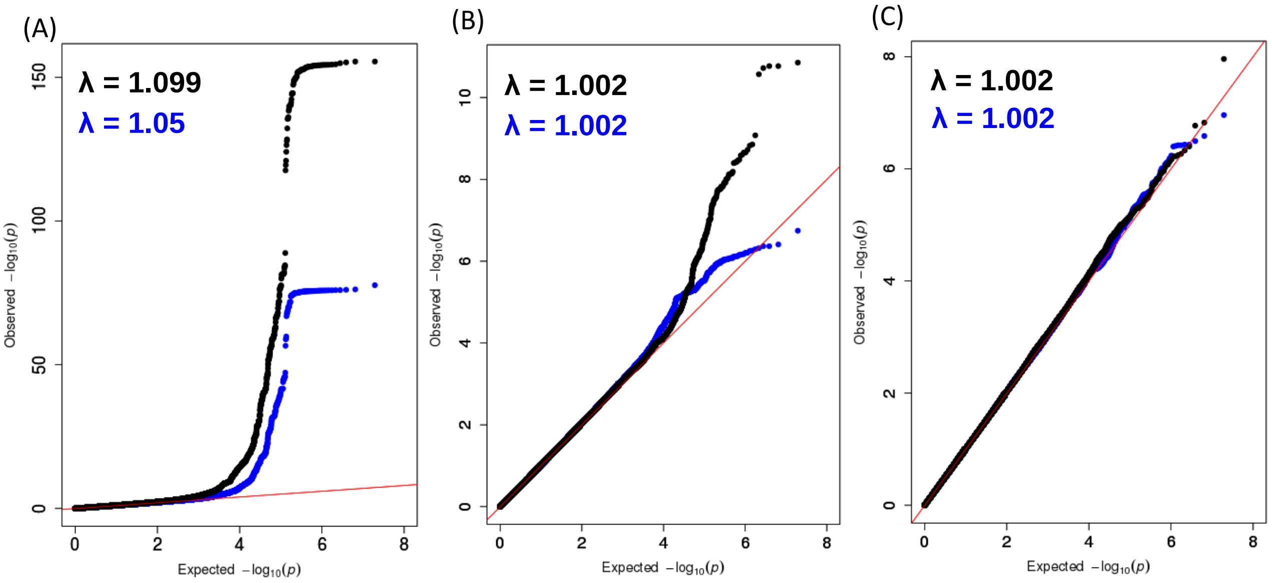

Figure S5: QQ plots for Tpe interval (A), Tpe dynamics during exercise (B) and Tpe dynamics during recovery (C) in the discovery (blue) and full (black) cohorts.

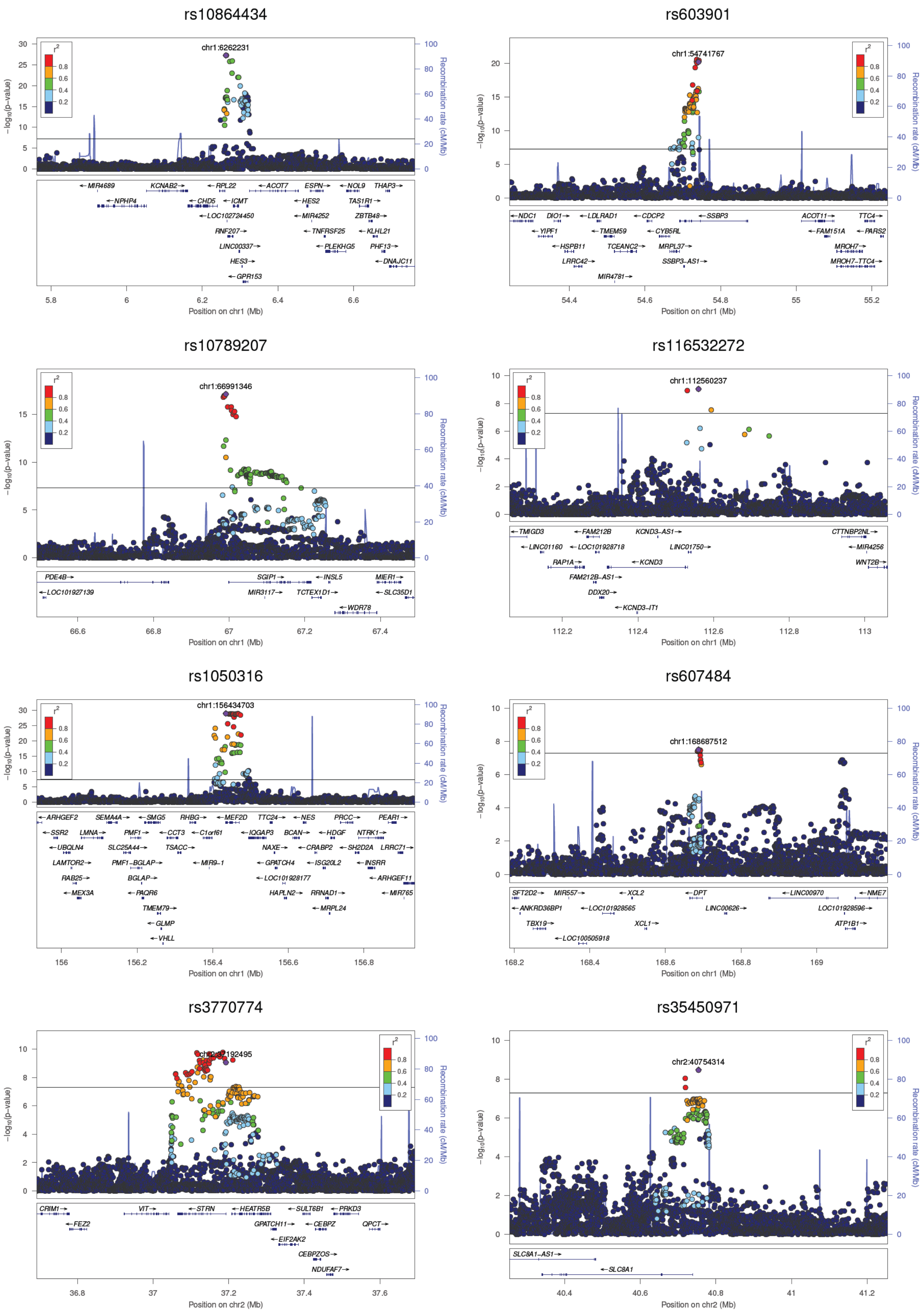
rs10864434
chr1:6262231
Position on chr1 (Mb)
rs603901
chr1:54741767
Position on chr1 (Mb)
rs10789207
chr1:66991346
Position on chr1 (Mb)
rs116532272
chr1:112560237
Position on chr1 (Mb)
rs1050316
chr1:156434703
Position on chr1 (Mb)
rs607484
chr1:168687512
Position on chr1 (Mb)
rs3770774
chr2:37192495
Position on chr2 (Mb)
rs35450971
chr2:40754314
Position on chr2 (Mb)
−log10(p−value)
Recombination rate (cM/Mb)

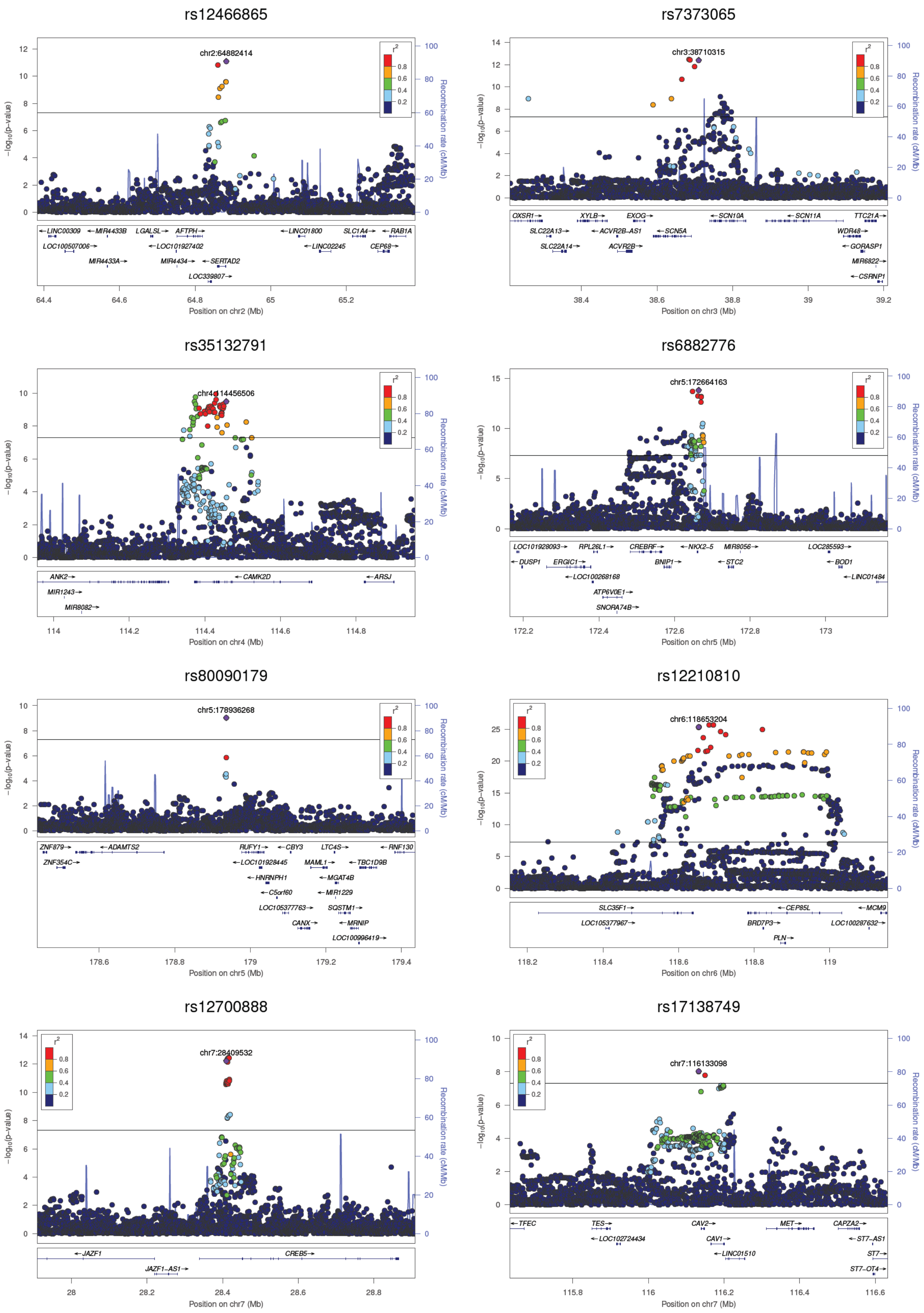

rs12466865
chr2:64882414
Position on chr2 (Mb)
rs7373065
chr3:38710315
Position on chr3 (Mb)
rs35132791
chr4:114456506
Position on chr4 (Mb)
rs6882776
chr5:172664163
Position on chr5 (Mb)
rs80090179
chr5:178936268
Position on chr5 (Mb)
rs12210810
chr6:118653204
Position on chr6 (Mb)
rs12700888
chr7:28409532
Position on chr7 (Mb)
rs17138749
chr7:116133098
Position on chr7 (Mb)
−log10(p−value)
Recombination rate (cM/Mb)
r2
0.8
0.6
0.4
0.2

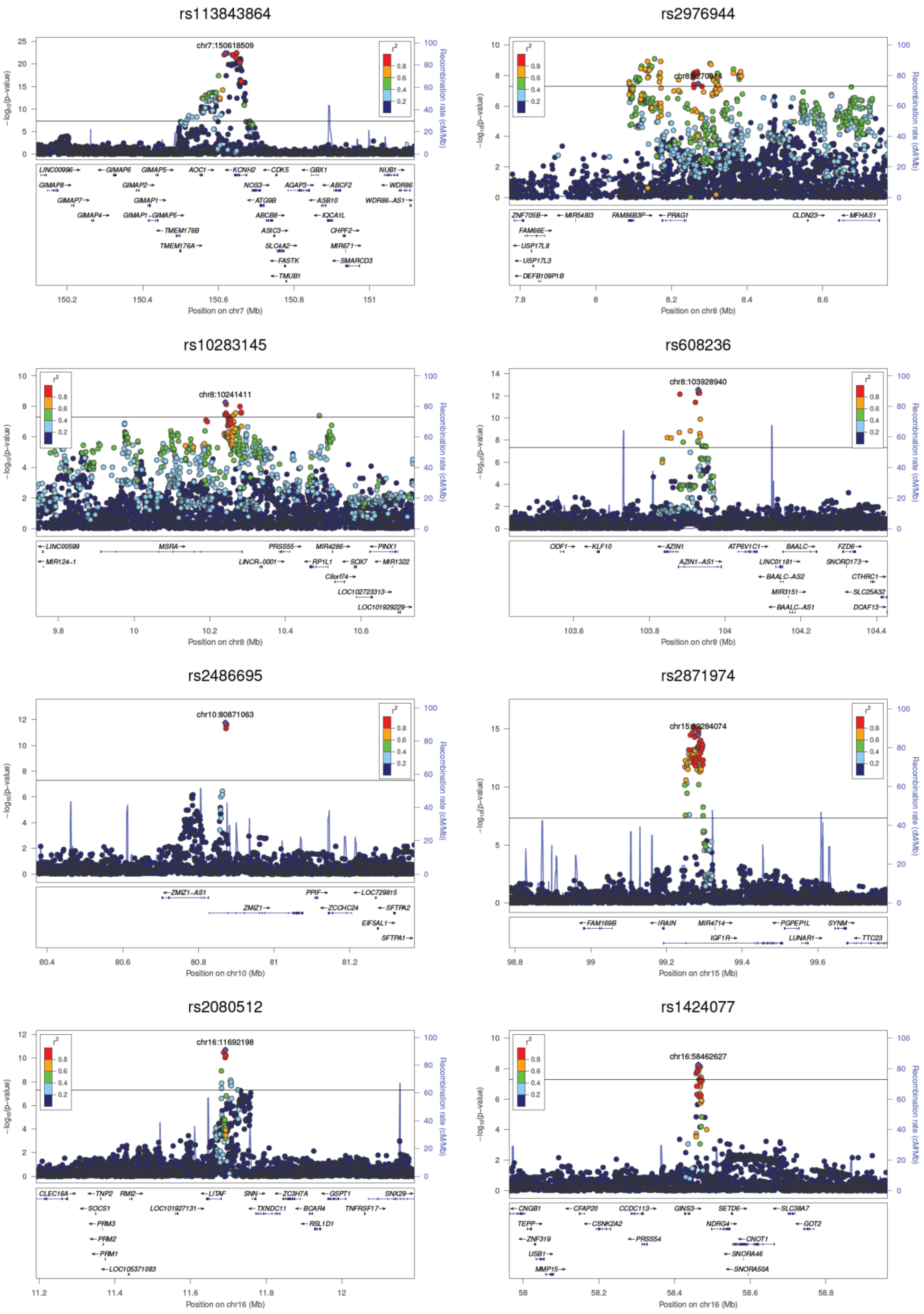

rs113843864
chr7:150618509
LINC00996 GIMAP6 GIMAP5 AOC1 KCNH2 CDK5 GBX1 NUB1
GIMAP8 GIMAP2 NOS3 AGAP3 ABCF2 WDR86
GIMAP7 GIMAP1 ATG9B ASB10 WDR86-AS1
GIMAP4 GIMAP1-GIMAP5 ABCB8 IQCA1L
TMEM176B ASIC3 CHPF2
TMEM176A SLC4A2 MIR671
FASTK SMARCD3
TMUB1
Position on chr7 (Mb)
rs2976944
chr8:8270914
ZNF705B MIR548I3 FAM86B3P PRAG1 CLDN23 MFHAS1
FAM66E
USP17L8
USP17L3
DEFB109P1B
Position on chr8 (Mb)
rs10283145
chr8:10241411
LINC00599 MSRA PRSS55 MIR4286 PINX1
MIR124-1 LINCR-0001 RP1L1 SOX7 MIR1322
C8orf74
LOC102723313
LOC101929229
Position on chr8 (Mb)
rs608236
chr8:103928940
ODF1 KLF10 AZIN1 ATP6V1C1 BAALC FZD6
AZIN1-AS1 LINC01181 SNORD173
BAALC-AS2 CTHRC1
MIR3151 SLC25A32
BAALC-AS1 DCAF13
Position on chr8 (Mb)
rs2486695
chr10:80871063
ZMIZ1-AS1 PPIF LOC729815
ZMIZ1 ZCCHC24 SFTPA2
EIF5AL1
SFTPA1
Position on chr10 (Mb)
rs2871974
chr15:99284074
FAM169B IRAIN MIR4714 PGPEP1L SYNM
IGF1R LUNAR1 TTC23
Position on chr15 (Mb)
rs2080512
chr16:11692198
CLEC16A TNP2 RMI2 LITAF SNN ZC3H7A GSPT1 SNX29
SOCS1 LOC101927131 TXNDC11 BCAR4 TNFRSF17
PRM3 RSL1D1
PRM2
PRM1
LOC105371083
Position on chr16 (Mb)
rs1424077
chr16:58462627
CNGB1 CFAP20 CCDC113 GINS3 SETD6 SLC38A7
TEPP CSNK2A2 NDRG4 GOT2
ZNF319 PRSS54 CNOT1
USB1 SNORA46
MMP15 SNORA50A
Position on chr16 (Mb)
−log10(p-value)
Recombination rate (cM/Mb)
r2

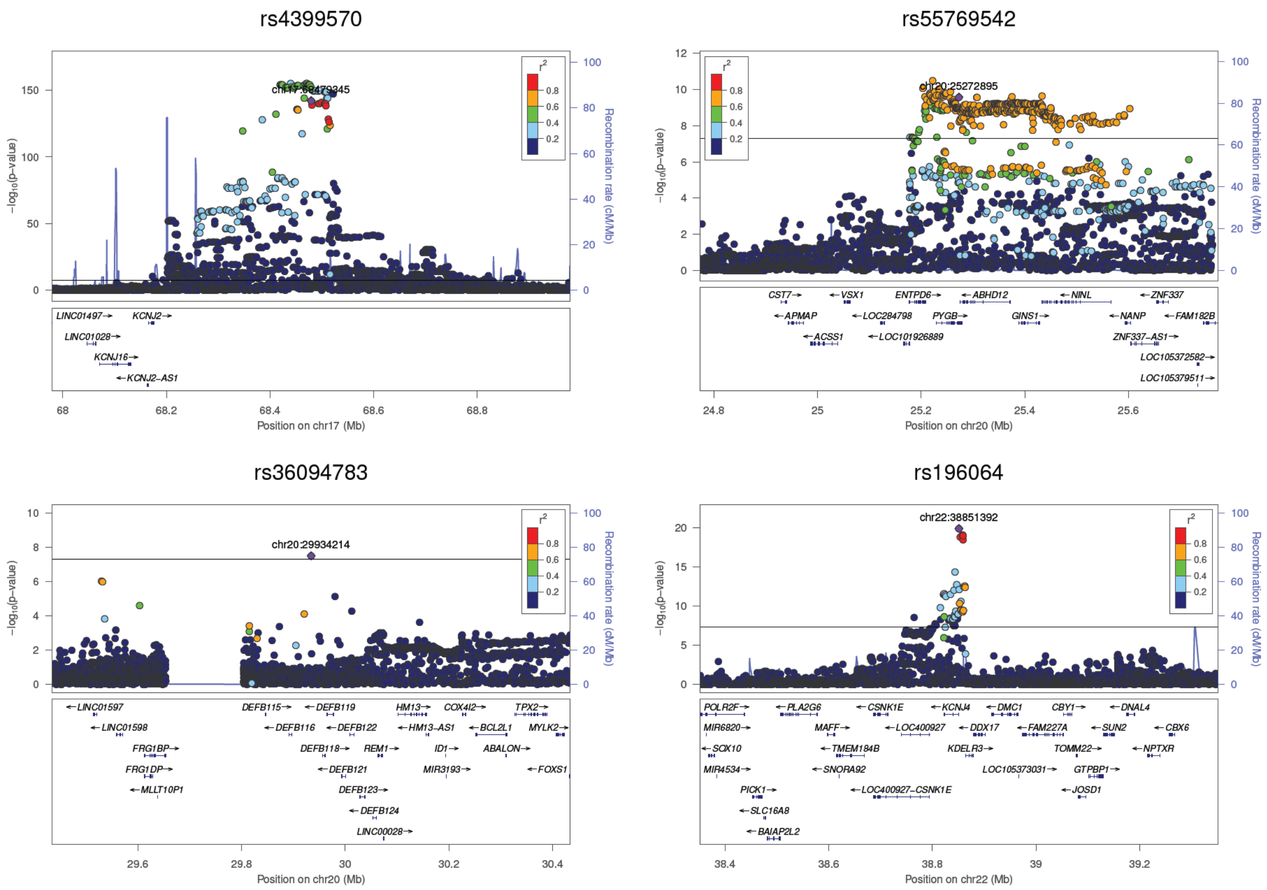

Figure S6: Locus Zoom plots for the twenty-eight (ordered by chromosome and base pair position) identified lead SNVs for resting Tpe.

rs607484

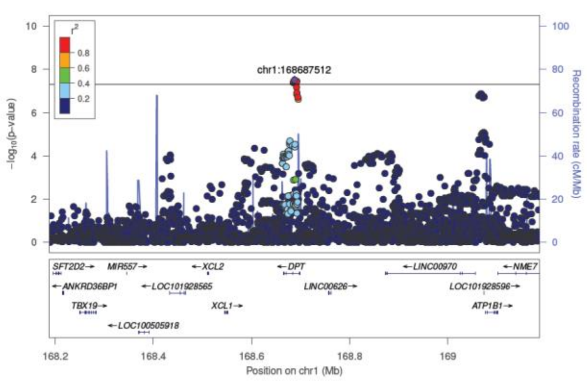

rs761499672

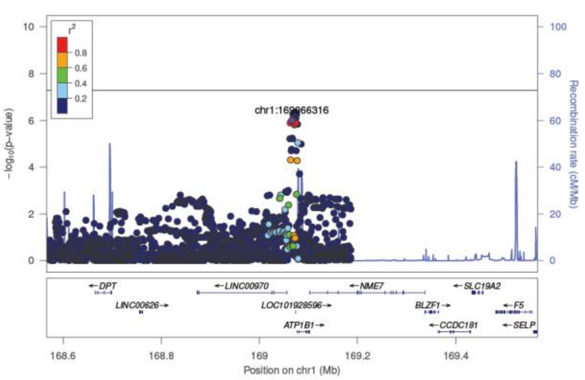

rs7373065

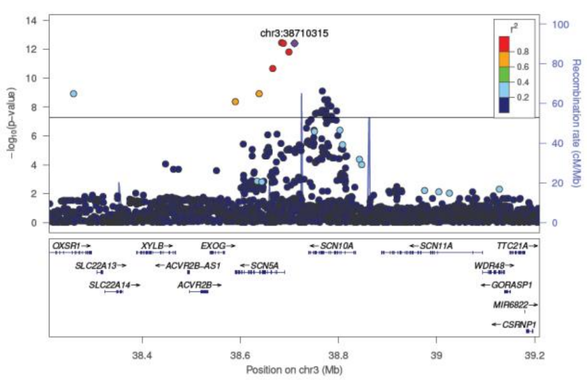

rs6801957

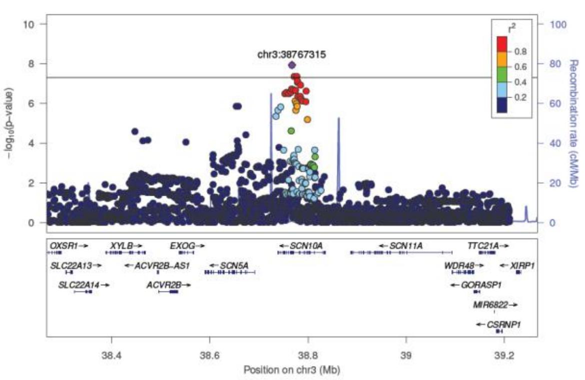

rs2080512

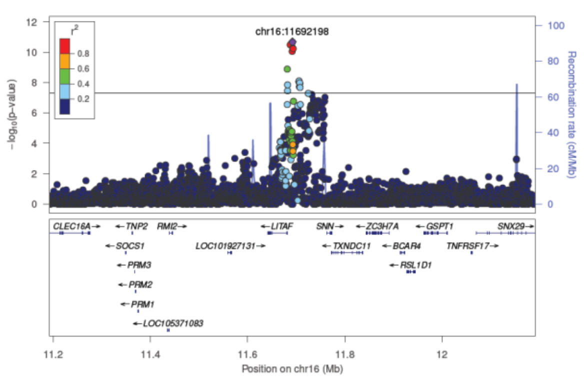

rs570620219

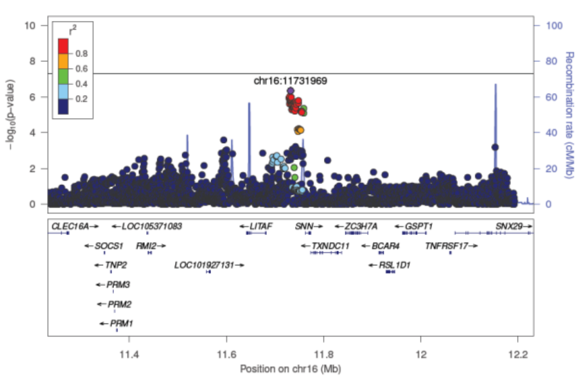

rs7373065

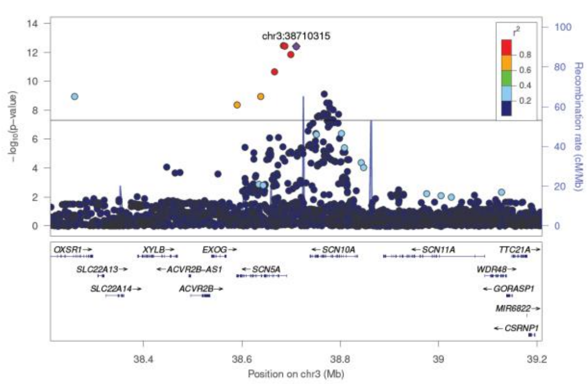

rs6801957

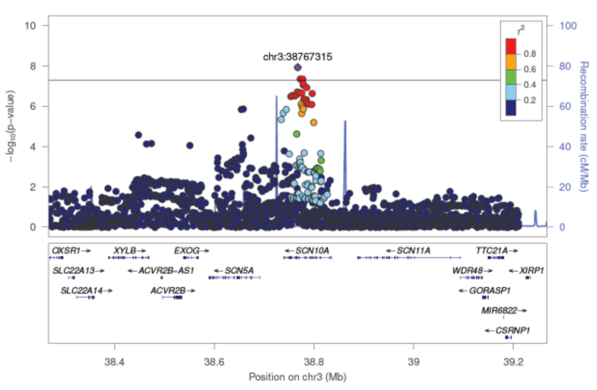

Figure S7: Locus Zoom plots for the four loci with secondary signals for resting Tpe.

Left: lead SNV. Right: LZ plot conditioned on the lead SNV.

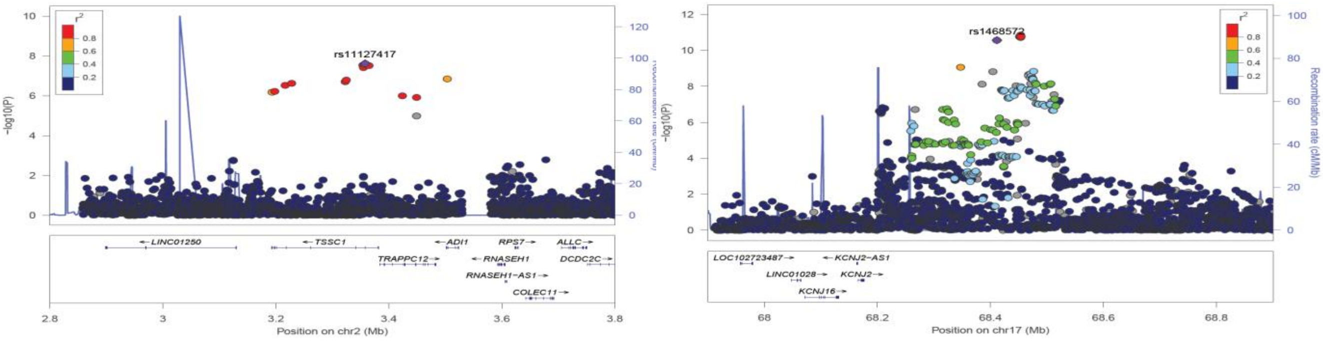

Figure S8: Locus Zoom plots for the identified loci for Tpe dynamics during exercise.

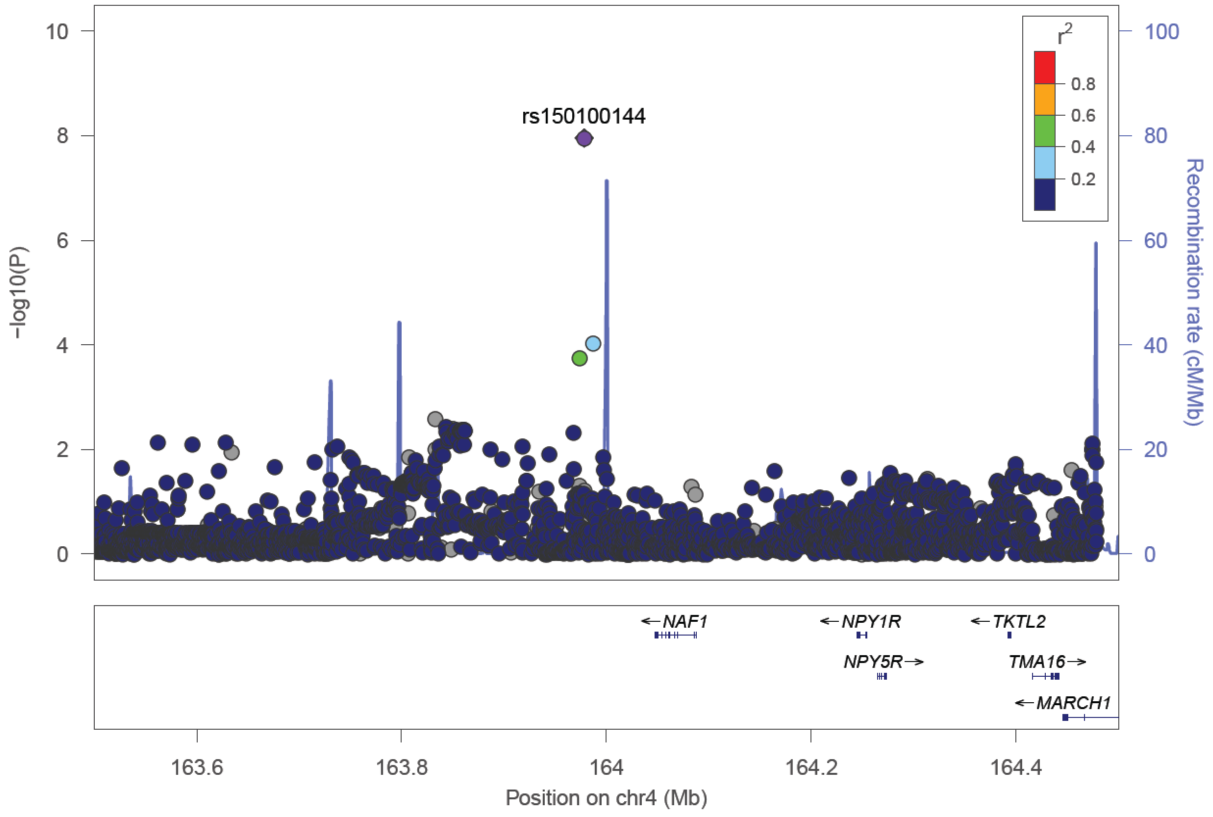

Figure S9: Locus Zoom plots for the identified locus for Tpe dynamics during recovery.

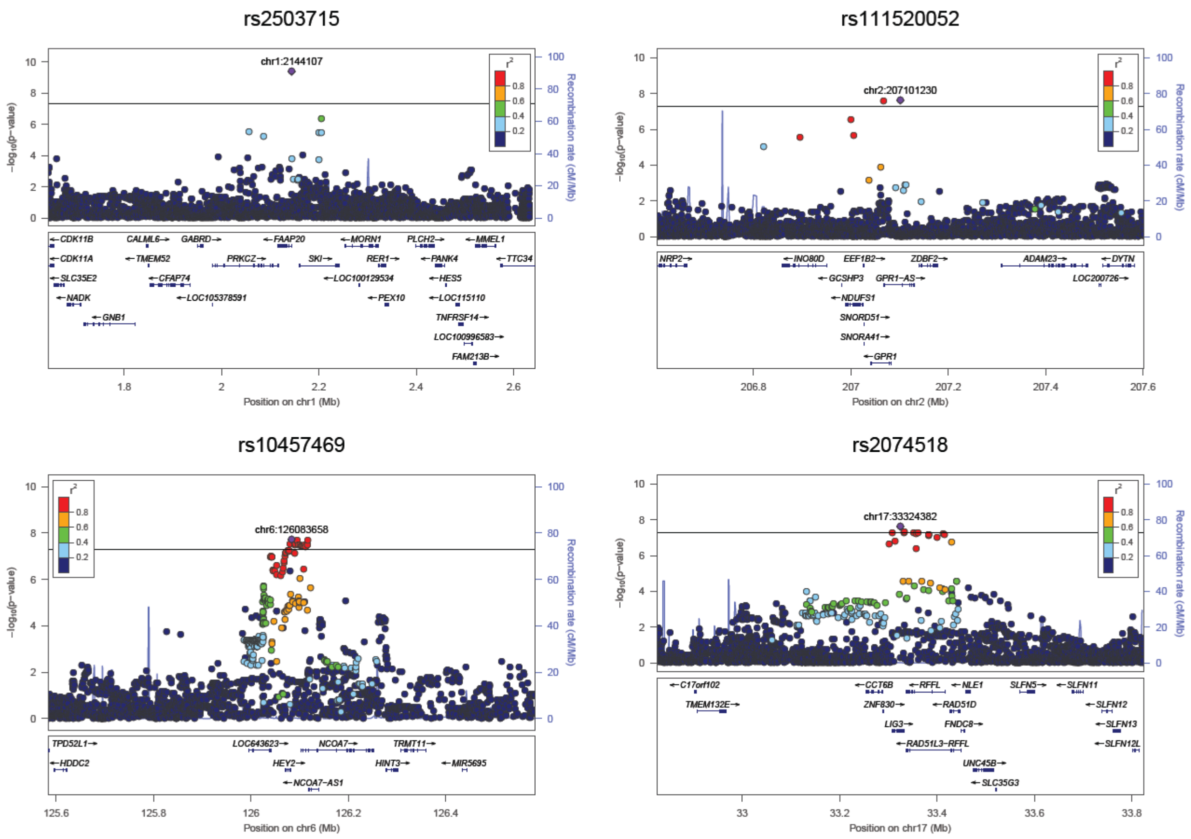

Figure S10: Locus Zoom plots for the male-specific loci for resting Tpe (males only summary statistics).

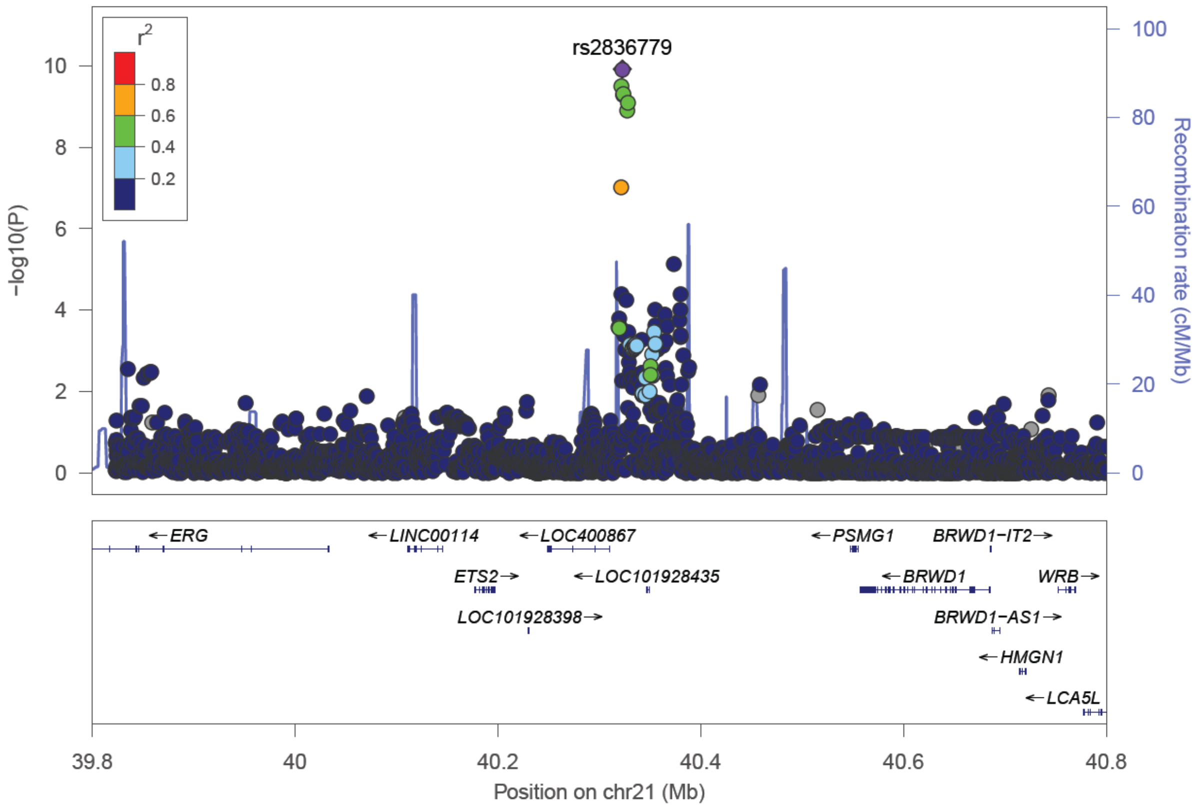

Figure S11: Locus Zoom plots for the sex-specific locus for Tpe dynamics during exercise (males only summary statistics).

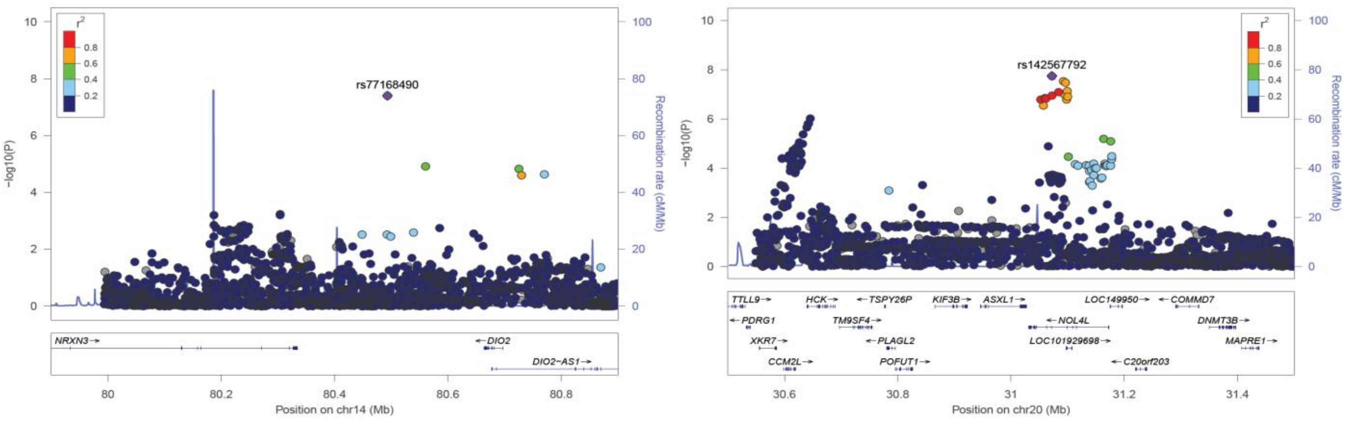

Figure S12: Locus Zoom plots for the sex-specific loci for Tpe dynamics during recovery (females only summary statistics).

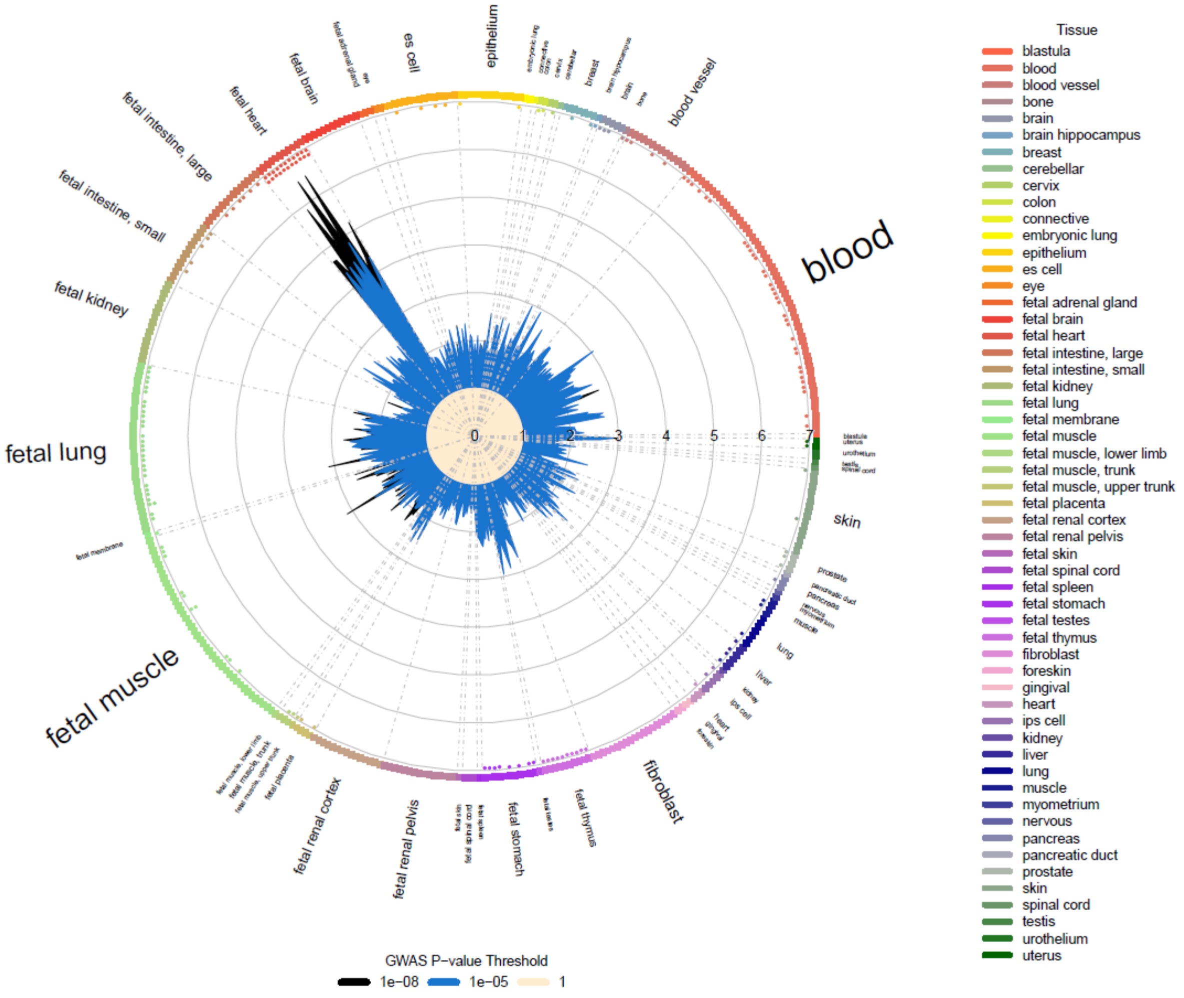

Figure S13: Functional enrichment analysis of resting Tpe loci within DNaseI hypersensitivity spots.

The radial lines show fold enrichment (FE) at eight GWA *P*-value thresholds. The results are shown for each of 424 cell types which are sorted by tissue, represented along the outer circle of the plot. The font size is proportional to the number of cell types from the tissue. FE values are plotted with different colours with respect to different GWA thresholds. Significant enrichment for a given cell type is denoted along the outer circle of the plot from a GWA *P*-value threshold.

source	term name	term ID	n. of term genes	n. of query genes	n. of common genes	corrected p-value	EIPR1	KCNJ2	ETS2
cor	ETS2-ETS1 complex	CORUM:2790	2	1	1	3.11e-03	?	?	co
cor	ETS2-ERG complex	CORUM:2789	2	1	1	3.11e-03	?	?	co
cor	ETS2-SMARCA4-INI1 complex	CORUM:5293	3	1	1	4.66e-03	?	?	co
cor	ETS2-FOS-JUN complex	CORUM:2695	3	1	1	4.66e-03	?	?	co
rea	Classical Kir channels	R-HSA-1296053	4	2	1	1.51e-02	?	r	
mir	hsa-miR-196b-5p	hsa-miR-196b-5p	151	3	2	1.93e-02		mi	mi
mir	hsa-miR-3646	hsa-miR-3646	199	3	2	3.35e-02		mi	mi
BP	regulation of skeletal muscle contraction by action potential	GO:0100001	1	2	1	3.77e-02	?	M	
BP	regulation of skeletal muscle contraction via regulation of action potential	GO:0014861	1	2	1	3.77e-02	?	M	

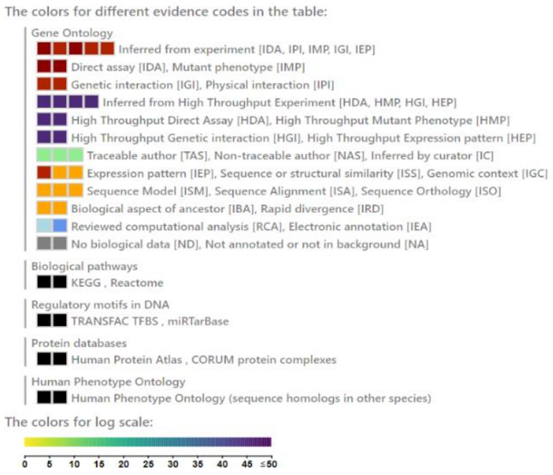

Figure S14: Top biological processes enrichment of candidate genes at Tpe dynamics during exercise loci.

g:profiler GO (gene ontology) term enrichment was performed using the candidate genes for Tpe dynamics during exercise.

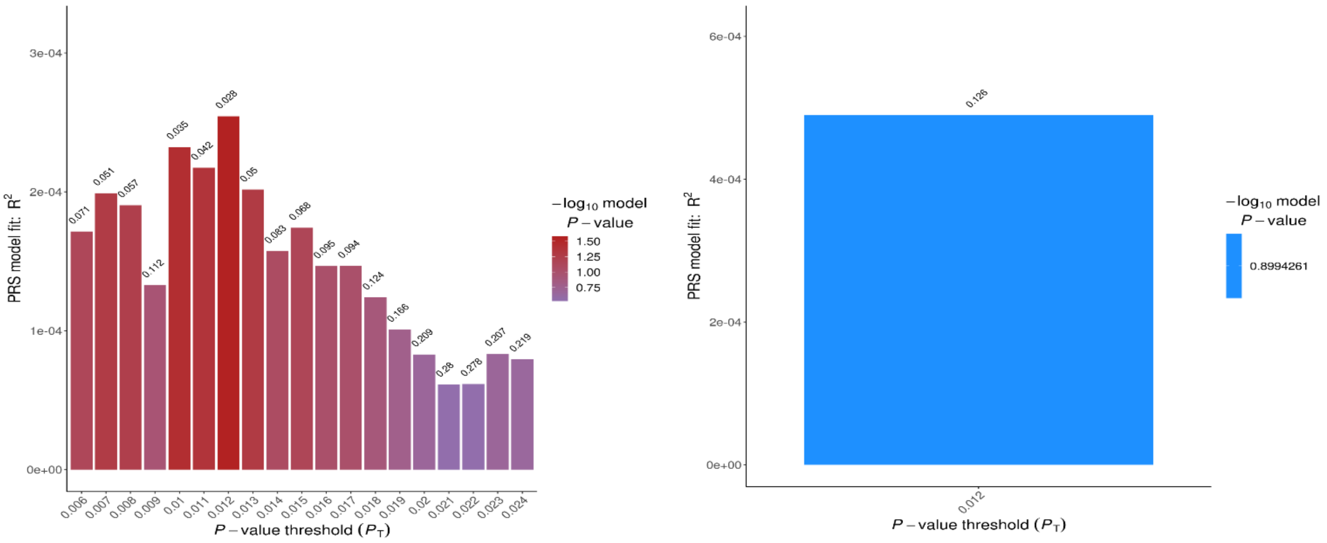

Figure S15: Left, adjusted R-squared versus the *P*-value threshold in the training subset (N = 247,256). The optimal cut-off value is the one for which the adjusted R-squared is highest and the *P*-value is lowest (*P* = 0.012). Right, adjusted R-squared and *P*-value of the logistic regression in the validation set (N = 68,563) using the optimal cut-off value.

Supplemental Tables

**Locus**	**SNV**	**CHR**	**BP**	**EA**	**MAF**	**Reported *P***	***P* in our study**	**N**	**β (ms)**	**PMID**
NOS1AP	rs2880058	1	162014632	A	-	1.00E-02	2.90E-03	5,890	-0.6	20215044
	rs4657139	1	162029907	T	-	3.00E-02	9.60E-04	5,890	-0.5	20215044
	rs10918594	1	162030688	C	-	2.00E-03	1.40E-03	5,890	-0.8	20215044
	rs10494366	1	162085685	T	-	2.00E-02	1.70E-04	5,890	-0.6	20215044
KCNH2	rs1805123	7	150645534	A	-	5.00E-05	4.30E-22	5,890	-1.2	20215044
	rs3807375	7	150667210	G	-	1.00E-03	2.20E-13	5,890	0.8	20215044
KCNE1	rs1805128	21	35821680	G	-	2.00E-01	1.40E-01	5890	-1.300	20215044
GRIN2A	rs17749681	16	9713930	C	0.380	2.10E-07	5.70E-01	1870	1.450	22342860

KCNJ2	**rs721966 9**	**17**	**70525720**	**T**	**0.380**	**1.10E-10**	**4.20E-148**	**1870**	**-1.790**	**22342860**

Table S1: Previously-reported loci associated with resting Tpe interval.

Abbreviations: SNV: single-nucleotide variation, CHR: Chromosome, BP: Position, based on HG build 19, EA: Effect allele, EAF: Effect allele frequency from discovery data, β: Beta, SE: Standard Error, N: number of participants, P: P-value.

The locus name indicates the gene that is in the closest proximity to the most associated SNV.

Bold indicates replicated in an independent study.

Only one genome-wide significant variant has been previously reported.

**Code**	**Definition**
I460	Cardiac arrest with successful resuscitation
I461	Sudden cardiac death, so described
I469	Cardiac arrest, unspecified
I472	Ventricular tachycardia
I490	Ventricular fibrillation and flutter
I499	Cardiac arrhythmia, unspecified

Table S2: ICD-10 codes used in follow-up analysis

						MALES				FEMALES				COMBINED			
Locus	SNV	CHR	BP	EA	EAF	β	SE	P	N	β	SE	P	N	β	SE	P	N
FAAP20	rs2503715	1	2144107	A	0.130	-0.072	0.012	4.20E-10	30532	-0.017	0.011	1.20E-01	34618	-0.043	0.008	6.20E-08	65150
RPL22	rs10864434	1	6262231	A	0.599	-0.052	0.008	1.70E-11	32176	-0.064	0.007	4.60E-18	36482	-0.058	0.005	5.80E-28	68658
SSBP3	rs562408	1	54742618	A	0.433	-0.052	0.008	4.60E-12	32610	-0.046	0.007	1.40E-10	36974	-0.049	0.005	3.20E-21	69584
SGIP1	rs10789207	1	66991346	T	0.785	-0.083	0.009	3.30E-20	33160	-0.026	0.009	2.50E-03	37597	-0.054	0.006	7.50E-18	70757
MEF2D	rs1050316	1	156434703	G	0.346	-0.074	0.008	2.90E-21	33270	-0.052	0.007	3.40E-12	37722	-0.061	0.005	9.40E-30	70993
STRN	rs66993681	2	37118665	C	0.456	0.026	0.008	6.50E-04	33012	0.040	0.007	3.10E-08	37430	0.033	0.005	2.20E-10	70441
SLC8A1	rs35450971	2	40754314	T	0.935	0.034	0.015	2.30E-02	32988	0.085	0.014	5.00E-09	37403	0.062	0.010	3.30E-09	70392
SERTAD2	2:64860029_CTTCAAA_C	2	64860029	CTTCAAA	0.669	-0.032	0.008	4.70E-05	32360	-0.043	0.008	1.60E-08	36690	-0.037	0.006	1.40E-11	69050
GPR1	rs111520052	2	207101230	G	0.988	0.190	0.034	2.40E-08	33011	0.056	0.033	9.10E-02	37429	0.117	0.024	7.30E-07	70440
SCN5A	rs7373065	3	38710315	T	0.019	-0.126	0.028	5.40E-06	31088	-0.155	0.027	7.90E-09	35248	-0.140	0.019	3.80E-13	66336
NKX2-5	rs6884881	5	172673319	T	0.433	-0.042	0.008	2.60E-08	32465	-0.026	0.007	2.60E-04	36810	-0.034	0.005	6.70E-11	69275
SLC35F1	rs12210810	6	118653204	G	0.945	0.124	0.016	2.60E-14	33432	0.114	0.016	2.00E-13	37906	0.118	0.011	4.60E-26	71338
HEY2	rs10457469	6	126083658	G	0.476	-0.042	0.007	1.90E-08	33350	-0.009	0.007	2.20E-01	37813	-0.024	0.005	1.80E-06	71162
CREB5	7:28413064_CT_C	7	28413064	CT	0.252	0.051	0.009	4.00E-09	32058	0.037	0.008	8.50E-06	36348	0.043	0.006	7.20E-13	68406
KCNH2	rs148064265	7	150626596	G	0.792	-0.068	0.009	1.10E-13	32897	-0.050	0.009	1.70E-08	37299	-0.059	0.006	1.20E-20	70196
AZIN1	rs608236	8	103928940	A	0.433	0.033	0.008	8.50E-06	33025	0.041	0.007	1.10E-08	37445	0.038	0.005	2.80E-13	70471
ZMIZ1	rs1658323	10	80874523	C	0.613	-0.030	0.008	8.30E-05	32152	-0.044	0.007	3.70E-09	36455	-0.038	0.005	2.20E-12	68607
IGF1R	rs4965430	15	99268850	C	0.375	-0.045	0.008	3.70E-09	33110	-0.040	0.007	9.00E-08	37541	-0.042	0.005	2.00E-15	70650
LITAF	rs2080512	16	11692198	G	0.538	-0.026	0.007	4.40E-04	33238	-0.041	0.007	5.80E-09	37686	-0.034	0.005	2.00E-11	70924
LIG3	rs2074518	17	33324382	C	0.545	0.042	0.007	2.40E-08	33432	0.009	0.007	1.90E-01	37906	0.025	0.005	1.60E-06	71338
KCNJ2	rs4399570	17	68479345	G	0.699	0.148	0.008	2.20E-74	33301	0.137	0.008	7.40E-71	37757	0.142	0.006	5.30E-143	71058
PYGB	rs3787080	20	25232604	C	0.741	-0.018	0.009	3.20E-02	33407	-0.051	0.008	1.50E-10	37878	-0.036	0.006	6.50E-10	71285
KCNJ4	rs196064	22	38851392	C	0.631	0.052	0.008	1.40E-11	33203	0.045	0.007	6.50E-10	37646	0.049	0.005	1.30E-20	70848

Table S4A: Sex-stratified analyses for resting Tpe interval.

Lead SNVs at *FAAP20*, *GPR1*, *HEY2* and *LIG3* (indicated in yellow) are male specific loci. These variants are non-significant ($P > 0.05$) in females only. They were not selected for replication in the discovery analysis, and were non-significant in the combined analyses.

						MALES				FEMALES				COMBINED			
Locus	SNV	CHR	BP	EA	EAF	β	SE	P	N	β	SE	P	N	β	SE	P	N
ETS2	**rs2836779**	**21**	**40322678**	**C**	**0.353**	**0.061**	**0.009**	**1.20E-10**	**24241**	**0.007**	**0.009**	**4.10E-01**	**27269**	**0.032**	**0.006**	**5.70E-07**	**51510**

Table S4B: Sex-stratified analyses for Tpe dynamics during exercise.

Lead SNV at *ETS2* (indicated in bold type) is a male specific locus. This variant is non-significant ($P > 0.05$) in females only. It was not selected for replication in the discovery analysis, and was non-significant in the combined analyses.

Abbreviations: SNV: single-nucleotide variant CHR: chromosome, BP: Base pair position, based on HG built 18, EA: effect allele, EAF: effect allele frequency, β: Beta in beats per minute, SE: Standard Error, N: effective number of participants, P: P-value.

						MALES				FEMALES				COMBINED			
Locus	SNV	CHR	BP	EA	EAF	β	SE	P	N	β	SE	P	N	β	SE	P	N
NRXN3	rs77168490	14	80493255	A	0.98	-0.032	0.033	3.26E-01	24172	-0.170	0.031	4.00E-08	27331	-0.102	0.022	5.00E-06	51503
NOL4L	rs565497590	20	31049031	G	0.987	0.025	0.040	5.22E-01	23377	0.214	0.037	1.10E-08	26432	0.119	0.027	8.90E-06	49808

Table S4C: Sex-stratified analyses for Tpe dynamics during recovery.

Lead SNVs at *NRXN3* and *NOL4L* (indicated in yellow) are female specific loci. The variants were not selected for replication from the discovery analyses and were non-significant in the combined analyses.

Abbreviations: SNV: single-nucleotide variant CHR: chromosome, BP: Base pair position, based on HG built 18, EA: effect allele, EAF: effect allele frequency, β: Beta in beats per minute, SE: Standard Error, N: effective number of participants, P: P-value.

									p_{12} = 1 x 10^{-6}	
**Locus**	**Lead SNV**	**CHR**	**BP**	**eQTL SNV**	**r2 (Lead SNV-eQTL SNV)**	**eQTL P-value**	**Tissue**	**Transcript**	**PP Different signal%**	**PP common signal%**
SSBP3	rs603901	1	54741767	rs590041	0.990	1.48E-10	Heart_Left_Ventricle	*SSBP3*	6	94
				rs562408	0.990	6.84E-23	Heart_Atrial_Appendage	*SSBP3*	5	95
SGIP1	rs10789207	1	66991346	rs72677052	0.995	9.43E-26	Heart_Left_Ventricle	*SGIP1*	7	93
NKX2-5	rs6882776	5	172664163	rs6891790	0.934	2.29E-09	Brain_Cerebellum	*NKX2-5*	9	91
HEY2	rs10457469	6	126083658	rs3757217	0.990	2.41E-13	Brain_Anterior_cingulate_cortex_BA24	*RP11-624M8.1*	35	60
				rs1811852	0.990	7.87E-09	Heart_Atrial_Appendage	*RP11-624M8.1*	37	57
				rs980014	0.979	4.04E-12	Brain_Amygdala	*RP11-624M8.1*	32	64
MSRA	rs10283145	8	10241411	rs6601450	0.890	4.03E-08	Brain_Hippocampus	*RP11-981G7.6*	40	59
IGF1R	rs2871974	15	99284074	rs6598541	0.972	5.30E-09	Heart_Left_Ventricle	*IGF1R*	6	94
LITAF	rs2080512	16	11692198	rs735951	0.992	9.85E-10	Heart_Left_Ventricle	*LITAF*	4	96
GINS3	rs1424077	16	58462627	rs2042401	0.999	9.62E-17	Brain_Caudate_basal_ganglia	*RP11-481J2.2*	8	92
				rs4784934	0.993	1.63E-17	Brain_Putamen_basal_ganglia	*RP11-481J2.2*	12	88
				rs9928581	0.996	3.15E-13	Heart_Atrial_Appendage	*NDRG4*	11	89
LIG3	rs2074518	17	33324382	rs12945428	0.948	7.04E-24	Heart_Left_Ventricle	*LIG3*	14	83
						7.30E-18	Brain_Cortex	*LIG3*	13	85
				rs2339123	0.934	4.07E-10	Brain_Anterior_cingulate_cortex_BA24	*LIG3*	15	82
				rs1003918	0.992	2.92E-12	Brain_Caudate_basal_ganglia	*LIG3*	17	80
						5.61E-17	Brain_Cerebellar_Hemisphere	*LIG3*	12	85
						5.61E-20	Brain_Cerebellum	*LIG3*	14	84
						3.27E-09	Brain_Hippocampus	*LIG3*	14	84
				rs978202	0.874	2.93E-11	Brain_Frontal_Cortex_BA9	*LIG3*	14	84
				rs1088450	0.933	4.01E-11	Brain_Hypothalamus	*LIG3*	13	85
				rs2074518	1.000	8.12E-12	Brain_Nucleus_accumbens_basal_ganglia	*LIG3*	12	85
						2.09E-09	Brain_Putamen_basal_ganglia	*LIG3*	13	85
						8.91E-23	Heart_Atrial_Appendage	*LIG3*	12	85

Table S5: Expression quantitative trait locus (eQTL) analysis for resting Tpe interval.

Resting Tpe variants with significant eQTLs and its corresponding genes are indicated. The results from proxy variants, with high LD ($r^2 \geq 0.8$) with the lead variant in the UK Biobank study were included if there was tissue expression data in addition to the lead variant. Results were filtered to those reaching a P value ≤ 5 x 10^{-8}. The source was Genotype-Tissue Expression (GTEx) Consortium v7, PubMed ID is 25954001. r^2: A measure for the linkage disequilibrium between the proxy and lead SNVs; P: P value for the association between the variant and RNA tissue expression.

Columns J and K show the posterior probability of different signal and common signal after applying colocalisation test at a prior probability of 1 x 10-6 that a variant is associated with both traits.

Yellow rows indicate loci with tissue specific eQTLs with a strong colocalisation support.

MeSH term	Name	MeSH first level term	MeSH second level term	Nominal P value	False discovery rate
A07.541	Heart	Cardiovascular System	Heart	1.87E-04	<0.01
A07.541 .560	Heart Ventricles	Cardiovascular System	Heart	2.38E-04	<0.01
A07.541 .358	Heart Atria	Cardiovascular System	Heart	4.45E-04	<0.05

Table S8: DEPICT tissue enrichment across all resting Tpe loci.

Supplemental Methods

Phenotypic and genetic QC

In the EST-UKB cohort, the exercise protocol was adapted according to participants' risk factors. Participants were only included in the study if they were allowed to cycle at 50% or 30% of their maximum workload (no risk to minimum risk). If the heart rate reached the pre-set maximum heart rate level (75% of age-predicted maximum heart rate), the test was stopped. Also, if the participant reported chest pain, felt faint, dizzy or unwell, the test was also stopped (https://biobank.ctsu.ox.ac.uk/crystal/docs/Cardio.pdf). We only included participants who terminated the exercise stress test with any discomfort and with a heart rate lower than the pre-set maximum heart rate level.

Individuals were excluded based on existing medical conditions known to affect heart rate (atrial fibrillation, history of myocardial infarction or heart failure, (supra)-ventricular tachycardia, atrioventricular nodal re-entrant tachycardia, second or third degree atrioventricular block, bundle branch block and use of a pacemaker), individuals with a previous cardiovascular event (matching the codes from Supplemental Table 1) and/or individuals on heart rate altering medications (non-dihydropyridine calcium antagonists (Anatomic Therapeutic Chemical (ATC) code C08D, digoxin (ATC code C01AA5), and amiodarone (ATC code C01BD01)). Individuals with an RR interval (inverse of heart rate) change between resting and peak exercise, or between peak exercise and recovery, less than 10 ms or poor quality ECG recording were also excluded.

Individuals with bad genotype quality, provided by UKB, i.e. high missingness or heterozygosity and discordance between the self-reported sex and the sex inferred from the genotypes were excluded[1]. We used the k-means function in R as a clustering algorithm, to objectively and statistically select the clusters according to information from PC1 and PC2. The k-means algorithm 'partitions the points into k groups such that the sum of squares from points to the assigned cluster centres in minimised'. Then, we applied k-means separately to

cluster according to each PC1 and PC2, and initially only with k=4, for a 4-way clustering, to correspond to the 4 main ethnic clusters within UKB: White, African, Asian and Chinese. We then created an overall clustering, according to the intersections of the PC1-kmeans clustering and the PC2-4means clustering, so that participants were only categorised as 'White' overall, if they were contained in the 'White' cluster for both PCA1 and PC2. Next, we created an overall 'Mixed/Other' cluster, for any participants, whose clustering differed between PC1 and PC2. Finally, we combined the PCA ancestry clusters with the self-reported ethnicity. Individuals were only included if the PCA-clustering results matched the self-reported ancestry. However, we count 'mixed', 'other' and 'missing' as being broad/uncertain self-reported ethnicity, which have now been validated more objectively from the genetic PCA data. We restricted our genetic analyses to individuals with European ancestry.

Genetic analyses

For each trait, we carefully selected covariates based on "a priori" physiological knowledge of the marker, and additional confounding factors were identified using linear regression. Selection of covariates was performed for each trait specifically. The main rationale is that the three traits are physiologically different. For example, resting Tpe quantifies the later stage of ventricular repolarization at rest, which is closely modulated by resting RR interval. In contrast, Tpe response to exercise and recovery quantify the changes in Tpe with exercise and recovery which highly dependent on the corresponding changes in heart rate. We also checked the influence of additional confounding factors, such as smoking, alcohol or diabetes and only included those that were significantly associated.

ECG lead placement during the exercise test

The cardio assessment involved a 3 leads (lead I, II, and III) ECG recording (AM-USB 6.5, Cardiosoft v6.51) at a frequency of 500 Hz. The ECG was recorded using four electrodes placed on the right and left antecubital fossa and wrist (Figure R1) and stored in an xml-file of Cardiosoft (https://biobank.ctsu.ox.ac.uk/crystal/docs/Cardio.pdf).

Genetic risk score analyses

Ventricular arrhythmic risk was defined as arrhythmic mortality or admission to hospital with a ventricular arrhythmic diagnosis. The exact International Classification of Diseases, Tenth Revision codes used to define ventricular arrhythmic events are presented in Table S2. Date of death was obtained from death certificates held by the National Health Service (NHS) Information Centre and the NHS Central Register Scotland for participants from England and Wales and participants from Scotland, respectively. Diagnoses were captured using the "Spell and Episode" category from the Hospital Episode Statistics records. This category contains main and secondary diagnoses, coded according to *ICD-10*, made during the hospital inpatient stay. The main diagnoses are those taken to be the main reason for hospital admission, while secondary diagnoses are more often contributory or underlying conditions. We used both the main and secondary diagnoses for recording prevalent and incident risk factors, conditions and events. Date of the event was defined as the date of the first diagnosis.

Variants with minor allele frequency < 0.05 and imputation quality ≤ 0.3 were removed from the calculation. PRSice clumped variants to obtain SNVs in linkage equilibrium ($r^2 < 0.1$) within a 250 kb window. Multiple GRSs were computed at a large number of GWAS *P*-value thresholds ranging from 1×10^{-4} to 0.5 with 5×10^{-5} increments. PRSice then performed a logistic regression analysis between each GRS and ventricular arrhythmic risk, adjusting for age, sex, diabetes, cholesterol, BMI, systolic blood pressure (SBP), the genotyping array and the 10 first genetic principal components. The optimal GRS was then chosen as the one with the smallest *P*-value.

Supplemental References

1. Bycroft, C., Freeman, C., Petkova, D., Band, G., Elliott, L.T., Sharp, K., Motyer, A., Vukcevic, D., Delaneau, O., O'Connell, J., et al. (2017). Genome-wide genetic data on ~500,000 UK Biobank participants. bioRxiv.
