# Supplementary material for: Common Genetic Variants Modulate the Electrocardiographic Tpeak-to-Tend Interval
Source: Am J Hum Genet. 2020 May 7;106(6):764–78. doi: 10.1016/j.ajhg.2020.04.009 (PMC7273524; doi:10.1016/j.ajhg.2020.04.009)
Supplement: Supplementary file 8 — Document S2. Article plus Supplemental Information [file mmc8.pdf]

ARTICLE

Common Genetic Variants Modulate the Electrocardiographic Tpeak-to-Tend Interval

Julia Ramírez,[1,2,5] Stefan van Duijvenboden,[1,2,5] William J. Young,[1,3] Michele Orini,[1,2,3,6] Pier D. Lambiase,[2,3,6] Patricia B. Munroe,[1,4,6,*] and Andrew Tinker[1,4,6,*]

Sudden cardiac death is responsible for half of all deaths from cardiovascular disease. The analysis of the electrophysiological substrate for arrhythmias is crucial for optimal risk stratification. A prolonged T-peak-to-Tend (Tpe) interval on the electrocardiogram is an independent predictor of increased arrhythmic risk, and Tpe changes with heart rate are even stronger predictors. However, our understanding of the electrophysiological mechanisms supporting these risk factors is limited. We conducted genome-wide association studies (GWASs) for resting Tpe and Tpe response to exercise and recovery in ~30,000 individuals, followed by replication in independent samples (~42,000 for resting Tpe and ~22,000 for Tpe response to exercise and recovery), all from UK Biobank. Fifteen and one single-nucleotide variants for resting Tpe and Tpe response to exercise, respectively, were formally replicated. In a full dataset GWAS, 13 further loci for resting Tpe, 1 for Tpe response to exercise and 1 for Tpe response to exercise were genome-wide significant ($p \leq 5 \times 10^{-8}$). Sex-specific analyses indicated seven additional loci. In total, we identify 32 loci for resting Tpe, 3 for Tpe response to exercise and 3 for Tpe response to recovery modulating ventricular repolarization, as well as cardiac conduction and contraction. Our findings shed light on the genetic basis of resting Tpe and Tpe response to exercise and recovery, unveiling plausible candidate genes and biological mechanisms underlying ventricular excitability.

Introduction

Sudden cardiac death is a leading cause of mortality and is responsible for approximately half of all deaths from cardiovascular disease.[1] Most importantly, the vast majority of sudden cardiac deaths occur in the general population without known traditional risk factors.[2] Guidelines exist for preventive strategies, such as insertion of implantable cardioverter defibrillators in high-risk patient groups.[3] However, risk stratification is heavily reliant on the assessment of left ventricular systolic function, which has low specificity, as opposed to the analysis of the electrophysiological substrate for arrhythmias.

The surface electrocardiogram (ECG) is a widely available non-invasive tool, which provides a rapid assessment of underlying cardiac electrophysiology and is therefore a useful method to infer arrhythmic risk. An abnormally prolonged Tpeak-to-Tend (Tpe) interval on the ECG is a risk factor for ventricular arrhythmic mortality and all-cause mortality, independent of age, sex, comorbidities, QRS duration, and corrected QT interval (MIM: 610141), not only in healthy subjects[4] but also individuals with acquired QT prolongation[5,6] and cardiac patients.[7–12] In addition, the response of the Tpe interval to heart rate has also been reported to be significantly associated with sudden cardiac death in patients with heart failure.[13,14]

Although the general view is that the Tpe interval and the T-wave more commonly reflect spatial dispersion of repolarization in different regions of the heart, the exact nature of this is disputed.[15–17] One pre-eminent suggestion is that it reflects differences in transmural repolarization, but this is largely based on the *ex vivo* ventricular wedge preparation and has not been reproduced in the intact heart.[16,18,19] Thus, novel approaches are needed to improve our understanding of the biology underpinning T-wave morphology and specifically Tpe in the intact human heart.

Prior work in twin studies has demonstrated that resting Tpe interval is heritable (52%–63%)[20] and, consequently, genetic analyses have been undertaken to uncover its genetic determinants, identifying five loci[21,22] (Table S1). However, no genome-wide association study (GWAS) has been performed for resting Tpe interval in relatively large cohorts (>6,000 individuals) and the genetic basis of Tpe response to exercise and to recovery has never been studied.

Our objective was to identify genetic variants significantly associated with three traits (Figure 1) in a large middle-aged population from the UK: (1) resting Tpe interval (n = 71,338), (2) Tpe response to exercise (n = 51,897), and (3) Tpe response to recovery (n = 51,503). We applied extensive bioinformatics analyses to investigate the main biological pathways linking the identified loci and the three traits.

[1]Clinical Pharmacology, William Harvey Research Institute, Barts and The London School of Medicine and Dentistry, Queen Mary University of London, London EC1M 6BQ, UK; [2]Institute of Cardiovascular Science, University College London, London WC1E 6BT, UK; [3]Barts Heart Centre, St Bartholomew's Hospital, London EC1A 7BE, UK; [4]NIHR Barts Cardiovascular Biomedical Research Unit, Barts and The London School of Medicine and Dentistry, Queen Mary University of London, London EC1M 6BQ, UK
[5]These authors contributed equally to this work
[6]These authors contributed equally to this work
*Correspondence: p.b.munroe@qmul.ac.uk (P.B.M.), a.tinker@qmul.ac.uk (A.T.)
https://doi.org/10.1016/j.ajhg.2020.04.009.
© 2020 The Authors. This is an open access article under the CC BY license (http://creativecommons.org/licenses/by/4.0/).

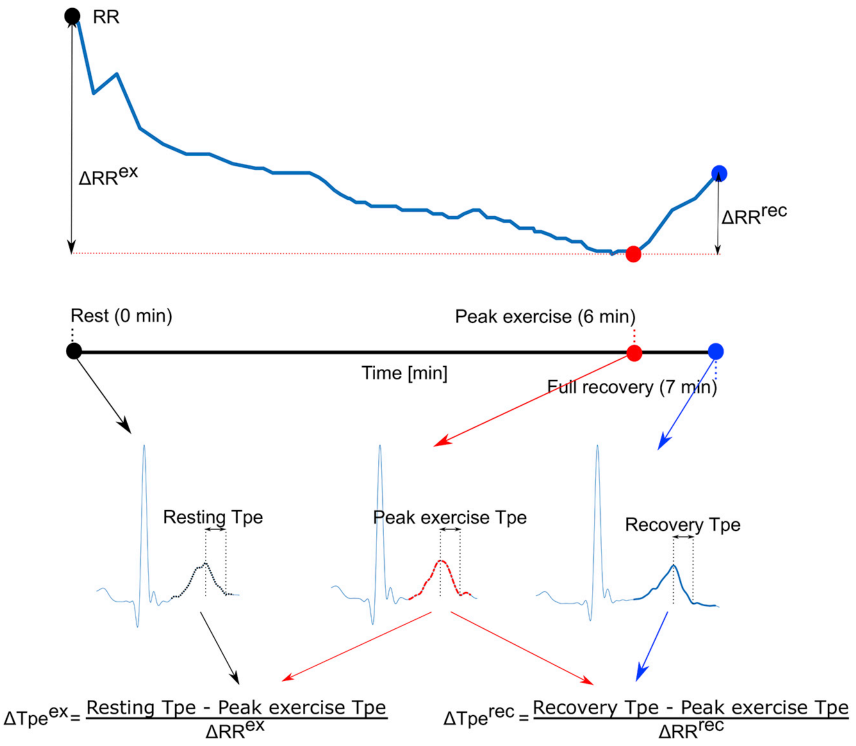

Figure 1. Assessment of Tpe Indices in the EST-UKB Cohort
(Top) Illustration of the RR profile during the exercise stress test. (Bottom) Three averaged heartbeats are derived at rest (black filled circle), peak exercise (red filled circle), and full recovery (blue filled circle), respectively. Resting, peak exercise, and recovery Tpe intervals were derived as the temporal differences between the corresponding T-wave offset and T-wave peak timing locations. Tpe dynamics during exercise was derived by quantifying the difference between the Tpe intervals at rest (black T-wave) and at peak exercise (red T-wave), normalized by the RR change during this interval. Similarly, Tpe dynamics during recovery was derived by quantifying the difference between the Tpe intervals at peak exercise (red T-wave) and full recovery (blue T-wave), normalized by the subsequent RR change.

Material and Methods

Anonymized data and materials have been returned to UK Biobank (UKB) and can be accessed per request.

UK Biobank

UKB is a prospective study of 488,377 volunteers comprising relatively even numbers of men and women aged 40–69 years old at recruitment (2006–2008). The UKB study has approval from the North West Multi-Centre Research Ethics Committee, and all participants provided informed consent.[23] The work was undertaken as part of UKB application 8256.

Genotyping was performed by UKB using the Applied Biosystems UK BiLEVE Axiom Array or the UKB AxiomTM Array.[24] Single-nucleotide variants (SNVs) were imputed centrally by UKB using the Haplotype Reference Consortium (HRC) and the 1000 Genomes Project (1000G) reference panels. Information on UKB array design and protocols is available on the UKB website (see Web Resources).

Participants were genotyped using a customized array (including GWAS and exome content) and with genome-wide imputation based on HRC and 1000G sequencing data.[25] A sub-cohort of 58,839 individuals completed an exercise test using a stationary bicycle in conjunction with an ECG recording (lead I, 2009, EST-UKB cohort). In parallel, a sub-cohort of 35,225 individuals participated in an imaging study (05/2014–03/2019; the collection is ongoing, IMAGE-UKB), which included 10 s 12-lead ECG recordings. All ECGs were acquired following the same protocol (see UKB website in Web Resources) and analyzed with the methods explained below.

Phenotypic and Genetic QC

Detailed information about the phenotypic and genetic quality control (QC) are indicated in Figure S1 and Supplemental Methods. Of the 56,385 individuals from EST-UKB who passed the phenotypic QC, 52,147 complied with genetic QC and were of European ancestry. Similarly, of the 26,467 individuals from IMAGE-UKB who passed the phenotypic QC, 24,999 complied with genetic QC and were of European ancestry. Then, 5,569 individuals who were in both the EST-UKB and IMAGE-UKB cohorts were excluded from the IMAGE-UKB cohort. After exclusions, there were 52,147 individuals from the EST-UKB cohort and 19,430 individuals from the IMAGE-UKB cohort available for genetic analyses (Figure S1).

Derivation of Resting Tpe and Tpe Response to Exercise and Recovery from the EST-UKB Cohort

The bicycle ergometer test followed a standardized protocol of 15 s resting period, followed by 6 min of exercise during which the workload was gradually increased, and a 1-min recovery period without pedalling. Pre-processing of the ECG signals from the EST-UKB cohort included low-pass filtering at 50 Hz to remove electric and muscle noise but still allow QRS detection.[26] Baseline wander was removed by further high-pass filtering the ECG signals at 0.5 Hz. Automatic quantification of resting Tpe and Tpe response to exercise and recovery (shown in Figure 1) was performed on every ECG recording in three steps:

(1) We signal-averaged the heartbeats within a window of 15 s during rest (black), at peak exercise (red), and 50 s after peak exercise (blue) to attenuate noise and artifacts and reveal small variations in the QRS-T-waveform. The onset, peak, and offset timings of the waveforms were located using bespoke software.[16,27]
(2) We derived resting, peak exercise, and recovery Tpe intervals as the temporal differences between the corresponding T-wave end and T-wave peak timing locations.
(3) Tpe response to exercise was derived by quantifying the difference between the Tpe intervals at rest (black T-wave)

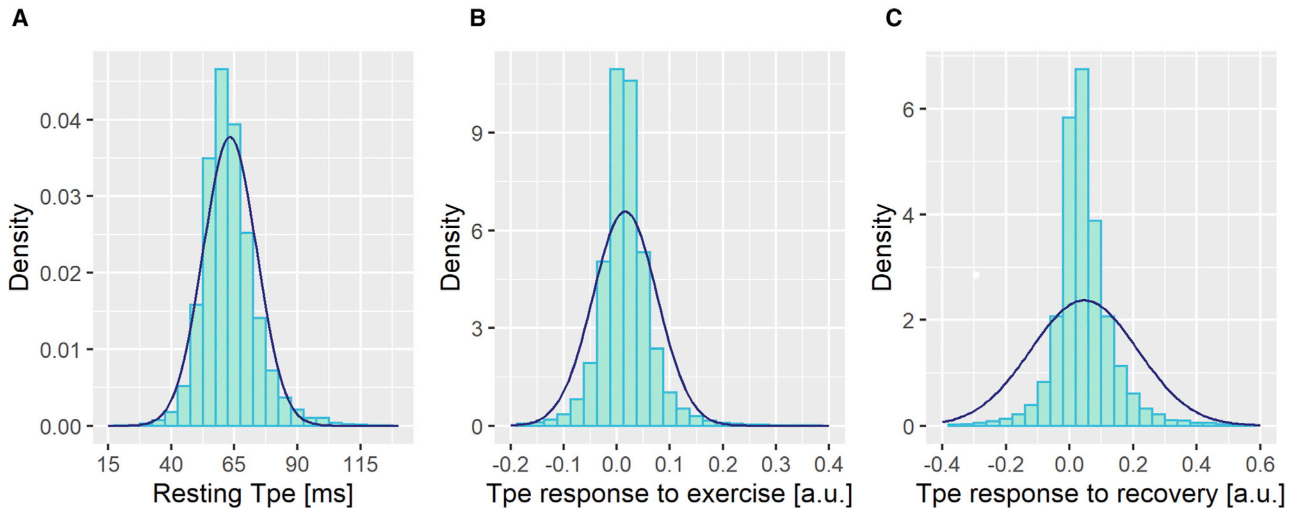

Figure 2. Density Plots of Tpe Phenotypes
Resting Tpe (A), Tpe dynamics during exercise (B), and Tpe dynamics during recovery (C). The blue curves indicate a normal distribution using the mean and standard deviation from each distribution.

and at peak exercise (red T-wave), normalized by the RR change during this interval, ΔRR^{ex}. Similarly, Tpe response to recovery was derived by quantifying the difference between the Tpe intervals at peak exercise (red T-wave) and full recovery (blue T-wave), normalized by the subsequent RR change, ΔRR^{rec}.

Derivation of Resting Tpe from the IMAGE-UKB Cohort

We chose lead I for analysis in the IMAGE-UKB cohort to match the EST-UKB signal. We removed baseline wander using a publicly available algorithm (see Web Resources). We pre-processed and signal averaged the heartbeats in the 10 s recordings as in the EST-UKB cohort. The onset, peak, and end timings of the waveforms were located using the same bespoke software as in previous studies.[16,27] Resting Tpe was derived as the temporal difference between the T-wave end and the T-wave peak timing locations.

For resting Tpe, we pooled the measurements from both EST-UKB and IMAGE-UKB cohorts together, leading to 71,338 individuals with resting Tpe. Inverse-normal transformation of resting Tpe and Tpe response to exercise and recovery was performed, as the distributions were skewed (Figure 2).

Genetic Analyses

An overview of the study design is provided in Figure 3. We randomly divided our cleaned datasets into discovery ($n \approx 30{,}000$) and replication ($n \approx 42{,}000$ for resting Tpe and $n \approx 22{,}000$ for Tpe response to exercise and to recovery) datasets. To ensure that there was no overlap across datasets, we removed first- and second-degree related individuals (kinship coefficient > 0.88) from the replication cohort as indicated from UKB.[24] We next selected model SNVs from directly genotyped SNVs using PLINK 1.9.[28] This selection was based on the following criteria: minor allele frequency (MAF) > 5%, a Hardy-Weinberg equilibrium with a threshold of p value $= 1 \times 10^{-6}$, and missingness < 0.0015. Model SNVs are used to learn the parameters from the mixed models for both the heritability estimation and the GWASs (see below).

Then, we estimated the proportion of resting Tpe, and Tpe response to exercise and recovery explained by additive genetic variation (heritability), as well as their genetic correlation with each other, using a variance components method (BOLT-REML),[29] with the model SNVs and ~9 million imputed variants with MAF ≥ 1% and INFO > 0.3 using the full cohorts (Figure 3).

Next, we performed a GWAS for each trait using a linear mixed model method (BOLT-LMM)[30] under the additive genetic model, including the model SNVs and ~9 million imputed SNVs with MAF ≥ 1% and INFO > 0.3 in the discovery dataset (Figure 3). For resting Tpe, we included the following covariates (details can be found in the Supplemental Methods): sex, age, body mass index (BMI), smoking status, resting RR, and a binary indicator variable for the genotyping array (UKB versus UK BiLEVE). For Tpe response to exercise, we included sex, age, BMI, diabetic status, resting RR, ΔRR^{ex}, and the genetic array. For Tpe response to recovery, we included sex, age, BMI, diabetic status, recovery RR, ΔRR^{rec}, and the genetic array.

Replication Analyses

All SNVs with $p < 1 \times 10^{-6}$ from the discovery GWAS for each trait were compiled and mapped to individual loci based on a genomic distance of >500 Kb to each side of another SNV. If multiple SNVs fitted the selection criteria for a single region, only the SNV with the smallest p value was taken forward into replication. As a QC step, we reviewed each selected SNV to check for unrealistically high effect sizes or large standard errors, and none were observed. Regional plots were produced for all selected SNVs and these were carefully reviewed. Twenty-one variants for resting Tpe, 4 variants for Tpe response to exercise, and 7 variants for Tpe response to recovery were taken forward into replication. Replication was confirmed if $p \leq 0.05/21 = 2.4 \times 10^{-3}$ for resting Tpe, $p \leq 0.05/4 = 1.3 \times 10^{-2}$ for Tpe response to exercise, and $p \leq 0.05/7 = 7.1 \times 10^{-3}$ for Tpe response to recovery and the effect was in the direction observed in discovery analyses for each trait in the replication cohort (Figure 3).

Full Dataset Analyses

In addition to the two-stage study design of discovery and replication cohorts, we also conducted a full dataset GWAS including all

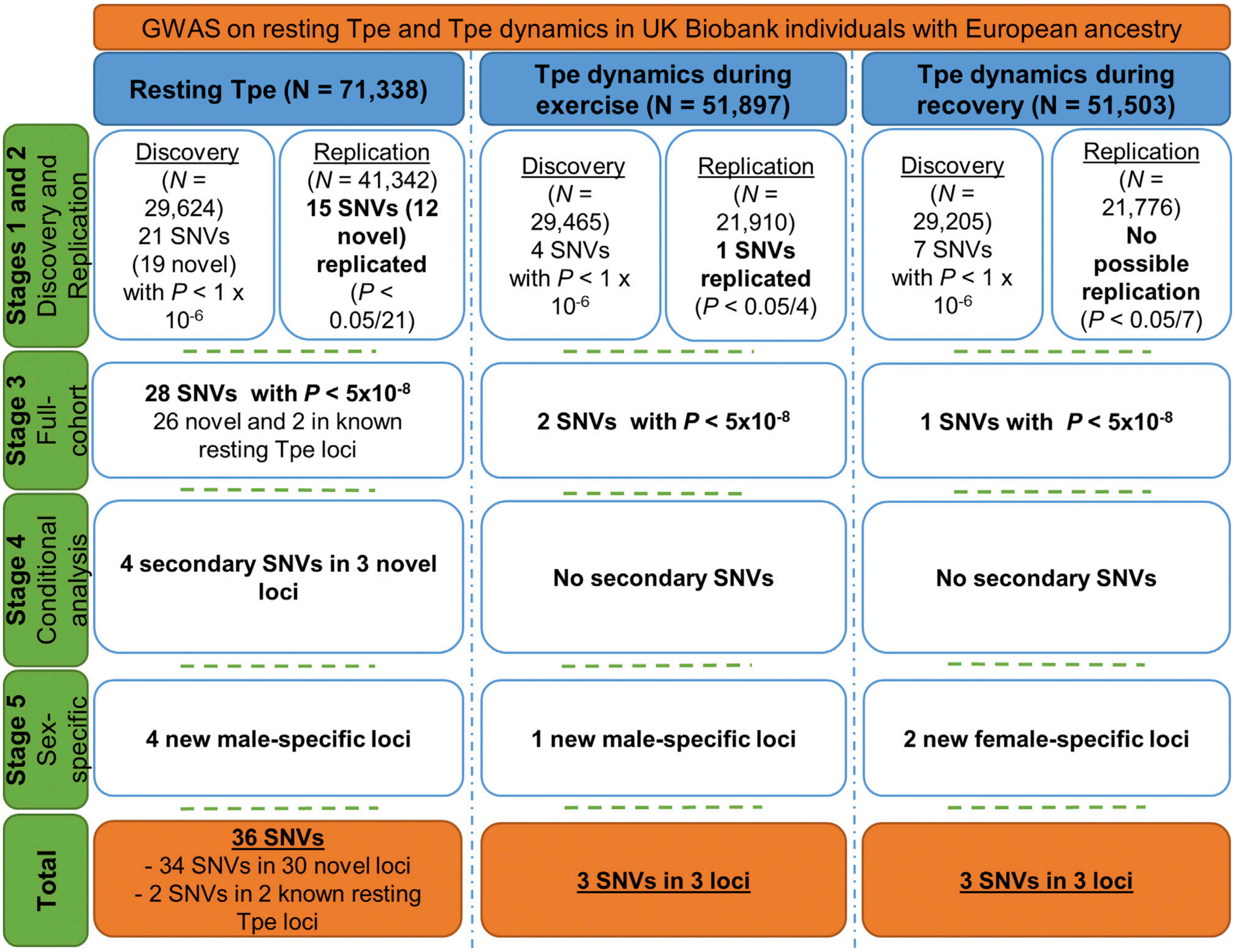

Figure 3. Analytical 5-Stage Approach Flowchart
Tpe, T-peak-to-Tend interval; SNV, single-nucleotide variant. Additional information can be found in Material and Methods.

individuals ($n = 71{,}338$ for resting Tpe, $n = 51{,}897$ for Tpe response to exercise, and $n = 51{,}503$ for Tpe response to recovery) using BOLT-LMM.[30] Additional loci for each trait reaching a genome-wide significance threshold ($p \leq 5 \times 10^{-8}$) from the full dataset GWAS are reported (Figure 3). To test for polygenicity, any underlying residual population stratification or QC factors affecting our GWAS results, we run LD Score Regression.[31]

Conditional Analyses

To examine whether there were independent SNVs at these loci, we applied genome-wide complex trait analysis.[32] We declared a secondary signal if (1) the identified SNV original p value was lower than 1×10^{-6}; (2) there was less than a 1.5-fold difference between the lead SNV and secondary association p values on a $-\log_{10}$ scale, i.e., if $-\log_{10}(p_{lead})/-\log_{10}(p_{sec}) < 1.5$; and (3) there was less than a 1.5-fold difference between the main association and conditional association p values on a $-\log_{10}$ scale, i.e., if $-\log_{10}(p_{sec})/-\log_{10}(p_{cond}) < 1.5$.[33]

Sex-Stratified Analyses

For each trait, we performed a GWAS for men and women separately in the full cohort including the same covariates in the regression model as specified above, but excluding sex (Figure 3).

Percent Variance Explained

The percent variance explained of each variant was calculated by estimating the residuals from the regression model against the covariates used in each respective genetic model. We then fitted a second linear model for the trait residuals with all the identified variants plus the top ten principal components. The percent variance explained was the difference between the adjusted R-squared parameters from each model.[33]

Bioinformatics Analyses

We performed several analyses to annotate the identified SNVs, at the variant and gene level (all SNVs in linkage disequilibrium [LD], $r^2 \geq 0.8$ with the traits associated SNVs were considered). LD was calculated using genetic data from UKB in order to calculate pairwise-LD for all associated SNVs. The r^2 of pairwise SNVs (minimum $r^2 = 0.8$ and maximum distance between a pair of SNVs is 4 Mb) were computed using PLINK.[28]

Using the University of California, Santa Cruz known genes, we annotated each lead SNV with the nearest coding genes and those located within 50 kb. At the variant level, we used Variant Effect Predictor[34] to obtain comprehensive functional characterization of variants, including their gene location, conservation, and amino acid substitution impact based on a range of prediction tools including SIFT and PolyPhen-2.

We evaluated all SNVs in LD ($r^2 \geq 0.8$) with our validated lead SNVs for evidence of mediation of expression quantitative trait loci (eQTL) using the GTEx database, focusing on loci with the strongest evidence of eQTL associations in brain, heart, and adrenal tissue. We also performed colocalization analyses using COLOC[35] including all SNVs within all loci with evidence of eQTLs in the relevant tissues and analyzed each eQTL-GWAS dataset pair. This tool is based on a Bayesian statistical methodology that tests pairwise colocalization of SNVs in GWAS with eQTLs and generates posterior probabilities for each locus weighting the evidence for competing hypothesis of either no colocalization or sharing of a distinct SNV at each locus. A posterior probability of $\geq$75% was considered strong evidence of the tissue-specific eQTL-GWAS pair influencing both the expression and GWAS trait at a particular region. We, then, identified variants with regulatory potential using RegulomeDB[36] and found genes whose promoter regions form significant chromatin interaction with them from a range of tissues, including brain, heart, and adrenal long-range chromatin interaction (Hi-C) data. We found the most significant promoter interactions for all potential regulatory SNVs (RegulomeDB score $\leq$ 5) in LD ($r^2 \geq 0.8$) with our sentinel SNVs and chose the interactors with the SNVs of highest regulatory potential to annotate the loci.

We also performed enrichment testing across all loci. We used DEPICT[37] to identify cells and tissues in which resting Tpe and Tpe response to exercise and to recovery loci were highly expressed. Due to the limited number of identified loci for Tpe response to exercise and to recovery, we used g:profiler[38] to perform functional profiling of gene lists using various kinds of biological evidence (including GO, HPO annotation). Enrichment results with false discovery rate < 5% were deemed significant.

Furthermore, to systematically characterize the functional, cellular, and regulatory contribution of genetic variation, we used GARFIELD,[39] analyzing the enrichment of genome-wide association summary statistics in tissue-specific functional elements at given significance thresholds.

The National Center for Biotechnology Information (NCBI) Gene database and GeneCards: The Human Gene Database were used to obtain official full names and, where relevant, common aliases for each candidate gene product. NCBI's PubMed was used to interrogate primary literature pertaining to gene function. We also reviewed gene-specific animal models using International Mouse Phenotyping Consortium[40] and the Mouse Genome Informatics database.

Finally, to explore shared mechanisms of disease, we assessed association of our identified SNVs (and their proxies, $r^2 \geq 0.8$) with other traits from published GWAS using PhenoScanner.[41] Our group has recently performed GWASs on the PR interval (MIM: 108980)[42] and on two traits related to the Tpe interval, T-wave morphology restitution during exercise, and during recovery,[43] but results are not yet available in PhenoScanner or GWAS Catalog. In addition, a recent paper also still not in PhenoScanner or GWAS Catalog reported genetic variants significantly associated with the QRS complex.[44] We, therefore, performed a lookup of the reported lead SNVs in our results to check for pleiotropy.

Genetic Risk Score Analyses

To evaluate the impact of a genetically prolonged Tpe interval on ventricular arrhythmic risk (definition can be found in Table S2 and in the Supplemental Methods), we split all remaining individuals from UKB into training (n = 274,256, 0.6% arrhythmic events) and validation (n = 68,563, 0.6% arrhythmic events) subsets. These were unrelated UKB individuals of European ancestry not included in the EST-UKB and IMAGE-UKB cohorts, who passed genetic QC, were free of a previous history of CV events, and were unrelated (FULL-UKB, Figure S2). This split was random, but we ensured a similar prevalence of events across both subsets. We obtained the optimal p value cut-off for the GRS using PRSice v.2[45] in the training subset (Supplemental Methods). We then applied logistic regression to test for an association between the GRS derived with the optimal p value cut-off and ventricular arrhythmic risk in the validation set.

Results

The median (interquartile range) values of resting Tpe in both the EST-UKB and the IMAGE-UKB cohorts was 62 (12) ms (Figure S3). Histograms showing the distribution of the three traits are provided in Figure 2. The heritability estimate of resting Tpe was 15.6%, and its genetic correlations were 0.30 with Tpe response to exercise and 0.11 with Tpe response to recovery. The heritability estimates of the Tpe responses to exercise and to recovery were relatively low, 2.2% and 2.4%, respectively, and their genetic correlation to each other was 0.55.

28 Genetic Loci Are Associated with Resting Tpe

In the discovery GWAS for resting Tpe, 12 loci were genome-wide significant ($p \leq 5 \times 10^{-8}$, Table S3). Using a significance threshold of $p < 1 \times 10^{-6}$, 21 variants (considering one lead SNV per 1 Mb region) were identified as significant and taken forward into replication in ~42,000 independent samples from UKB. Of the 21 selected SNVs for resting Tpe, 15 formally replicated ($p \leq 0.05/21 = 2.4 \times 10^{-3}$) and all had concordant directions of effect (Table 1).

Thirteen additional SNVs (also considering one lead SNV per 1 Mb region) reached genome-wide significance in the full dataset GWAS, all with concordant directions of effect across discovery, validation, and full cohort datasets (Table 1). Manhattan plots in the full dataset GWAS results are shown in Figure S4A, and QQ plots including the discovery (blue) and full dataset (black) GWASs are shown in Figure S5. The intercept of the LD Score Regression[31] was 1.005 (standard error of 0.0097), indicating inflation of the lambdas is predominantly due to polygenicity and not to underlying QC factors or population stratification. Regional plots are shown in Figure S6.

Conditional analyses showed evidence for four secondary independent signals at loci *DPT* (MIM: 125597), *SCN5A-SCN10A* (MIM: 600163), and *LITAF* (MIM: 603795, Table 1, Figure S7). The secondary signal at *DPT*, rs761499672, was located 379 kb away from the lead SNV, rs607484; while the secondary signals at *SCN5A-SCN10A*, rs6797133 and rs6801957, were located 54 kb

Table 1. Discovery, Replication, and Full GWAS Results for the Lead SNVs for Resting Tpe Interval

						Discovery					Replication					Combined				
Locus	SNV	CHR	BP	EA	AA	EAF	β	SE	p	n	EAF	β	SE	p	n	EAF	β	SE	p	n
RNF207	**rs10864434**	**1**	**6262231**	**A**	**T**	**0.602**	**−0.066**	**0.008**	**1.20E−15**	**28,511**	**0.600**	**−0.054**	**0.007**	**2.50E−14**	**39,789**	**0.601**	**−0.058**	**0.005**	**5.80E−28**	**68,658**
SSBP3	**rs603901**	**1**	**54741767**	**C**	**T**	**0.434**	**−0.059**	**0.008**	**3.80E−13**	**29,111**	**0.436**	**−0.040**	**0.007**	**7.30E−09**	**40,626**	**0.435**	**−0.049**	**0.005**	**5.70E−21**	**70,103**
SGIP1	**rs10789207**	**1**	**66991346**	**T**	**C**	**0.787**	**−0.065**	**0.010**	**2.30E−11**	**29,383**	**0.784**	**−0.043**	**0.008**	**1.50E−07**	**41,005**	**0.786**	**−0.054**	**0.006**	**7.50E−18**	**70,757**
KCND3	rs116532272	1	112560237	G	A	0.986	0.133	0.036	1.90E−04	26,964	0.986	0.140	0.030	3.20E−06	37,629	0.986	0.140	0.023	9.10E−10	64,931
MEF2D	**rs1050316**	**1**	**156434703**	**G**	**T**	**0.345**	**−0.070**	**0.008**	**1.30E−16**	**29,481**	**0.349**	**−0.055**	**0.007**	**1.40E−14**	**41,142**	**0.347**	**−0.061**	**0.005**	**9.40E−30**	**70,993**
DPT†	rs607484	1	168687512	T	C	0.733	0.042	0.009	2.90E−06	29,624	0.734	0.024	0.008	1.90E−03	41,342	0.733	0.032	0.006	3.10E−08	71,338
STRN	**rs3770774**	**2**	**37192495**	**T**	**C**	**0.518**	**−0.040**	**0.008**	**7.90E−07**	**29,310**	**0.520**	**−0.029**	**0.007**	**2.30E−05**	**40,903**	**0.519**	**−0.032**	**0.005**	**8.20E−10**	**70,581**
SLC8A1	rs35450971	2	40754314	T	C	0.936	0.068	0.016	3.20E−05	29,231	0.935	0.056	0.014	4.60E−05	40,794	0.935	0.062	0.010	3.30E−09	70,392
SERTAD2	rs12466865	2	64882414	C	T	0.639	−0.029	0.008	6.00E−04	28,778	0.639	−0.042	0.007	4.80E−09	40,161	0.639	−0.037	0.005	7.70E−12	69,301
SCN5A-SCN10A†	**rs7373065**	**3**	**38710315**	**T**	**C**	**0.020**	**−0.149**	**0.030**	**5.70E−07**	**27,547**	**0.019**	**−0.143**	**0.026**	**2.60E−08**	**38,443**	**0.019**	**−0.140**	**0.019**	**3.80E−13**	**66,336**
CAMK2D	**rs35132791**	**4**	**114456506**	**C**	**G**	**0.744**	**−0.053**	**0.009**	**8.90E−09**	**29,455**	**0.740**	**−0.025**	**0.008**	**9.90E−04**	**41,107**	**0.742**	**−0.037**	**0.006**	**3.10E−10**	**70,932**
NKX2-5	rs6882776	5	172664163	G	A	0.720	−0.039	0.009	1.10E−05	29,157	0.716	−0.050	0.008	5.70E−11	40,690	0.717	−0.044	0.006	1.50E−14	70,213
RUFY1	rs80090179	5	178936268	T	G	0.989	−0.170	0.040	2.10E−05	27,892	0.989	−0.144	0.034	2.00E−05	38,925	0.989	−0.156	0.026	9.50E−10	67,167
SLC35F1	**rs12210810**	**6**	**118653204**	**G**	**C**	**0.944**	**0.106**	**0.018**	**1.30E−09**	**29,624**	**0.945**	**0.123**	**0.015**	**1.20E−16**	**41,342**	**0.945**	**0.118**	**0.011**	**4.60E−26**	**71,338**
CREB5	**rs12700888**	**7**	**28409532**	**A**	**C**	**0.264**	**0.047**	**0.009**	**2.00E−07**	**29,355**	**0.261**	**0.040**	**0.008**	**2.30E−07**	**40,967**	**0.262**	**0.042**	**0.006**	**5.80E−13**	**70,691**
CAV2	rs17138749	7	116133098	A	C	0.838	−0.036	0.011	7.50E−04	29,511	0.837	−0.042	0.009	6.00E−06	41,184	0.838	−0.040	0.007	9.30E−09	71,065
KCNH2	**rs113843864**	**7**	**150618509**	**G**	**A**	**0.752**	**0.077**	**0.009**	**6.70E−17**	**29,582**	**0.752**	**0.047**	**0.008**	**2.60E−09**	**41,283**	**0.752**	**0.059**	**0.006**	**4.10E−23**	**71,236**
PRAG1	**rs2976944**	**8**	**8270914**	**T**	**C**	**0.486**	**−0.040**	**0.008**	**8.10E−07**	**29,199**	**0.489**	**−0.025**	**0.007**	**2.60E−04**	**40,749**	**0.488**	**−0.028**	**0.005**	**3.50E−08**	**70,315**
MSRA	rs10283145	8	10241411	C	T	0.484	0.027	0.008	8.20E−04	29,386	0.482	0.034	0.007	4.80E−07	41,009	0.483	0.030	0.005	5.50E−09	70,764
AZIN1	rs608236	8	103928940	A	G	0.433	0.030	0.008	2.70E−04	29,264	0.433	0.044	0.007	1.30E−10	40,839	0.433	0.038	0.005	2.80E−13	70,471
ZMIZ1	rs2486695	10	80871063	G	A	0.612	−0.038	0.008	3.50E−06	29,466	0.615	−0.038	0.007	4.70E−08	41,121	0.613	−0.037	0.005	1.70E−12	70,957
IGF1R	**rs2871974**	**15**	**99284074**	**C**	**T**	**0.363**	**−0.054**	**0.008**	**1.00E−10**	**29,500**	**0.358**	**−0.032**	**0.007**	**5.40E−06**	**41,169**	**0.360**	**−0.042**	**0.005**	**2.90E−15**	**71,040**
LITAF†	rs2080512	16	11692198	G	T	0.538	−0.035	0.008	1.60E−05	29,452	0.539	−0.031	0.007	3.40E−06	41,102	0.538	−0.034	0.005	2.00E−11	70,924
GINS3	rs1424077	16	58462627	G	A	0.273	−0.050	0.009	1.70E−08	29,486	0.275	−0.020	0.008	7.50E−03	41,150	0.274	−0.033	0.006	5.60E−09	71,006
KCNJ2*	**rs4399570**	**17**	**68479345**	**G**	**A**	**0.698**	**0.162**	**0.009**	**2.20E−78**	**29,508**	**0.699**	**0.125**	**0.007**	**1.60E−64**	**41,180**	**0.699**	**0.142**	**0.006**	**5.30E−143**	**71,058**
PYGB	**rs55769542**	**20**	**25272895**	**C**	**CA**	**0.674**	**−0.047**	**0.009**	**3.70E−07**	**25,533**	**0.674**	**−0.030**	**0.008**	**8.30E−05**	**35,633**	**0.674**	**−0.037**	**0.006**	**2.60E−10**	**61,487**

(*Continued on next page*)

Table 1. *Continued*

						Discovery					Replication					Combined				
DEFB118	rs36094783	20	29934214	G	A	0.932	−0.068	0.016	2.50E−05	28,334	0.932	−0.050	0.014	2.70E−04	39,541	0.932	−0.057	0.010	3.20E−08	68,231
KCNJ4	**rs196064**	**22**	**38851392**	**C**	**T**	**0.632**	**0.049**	**0.008**	**3.80E−09**	**29,421**	**0.634**	**0.049**	**0.007**	**3.00E−12**	**41,058**	**0.633**	**0.049**	**0.005**	**1.30E−20**	**70,848**

Abbreviations: SNV, single-nucleotide variation; CHR, chromosome; BP, position, based on HG build 19; EA, effect allele; AA, alternate allele; EAF, effect allele frequency from discovery data; β, beta; SE, standard error; n, number of participants; p, p value. The locus name indicates the gene that is in the closest proximity to the most associated SNV. Replicated SNVs in the replication cohort are indicated in bold type.
[a]Indicates has a secondary signal. The secondary signal at DPT, rs761499672, was located 379 kb away from the lead SNV, rs607484, while the secondary signals at SCN5A-SCN10A, rs6797133 and rs6801957, were located 54 kb and 57 kb, respectively, away from the lead SNV (rs7373065); and the secondary signal at LITAF, rs570620219, was located 40 kb away from the lead SNV (rs2080512).
[b]Lead SNV is in moderate LD ($r^2 = 0.56$) with the lead SNV for Tpe response to exercise.

and 57 kb, respectively, away from the lead SNV (rs7373065). Finally, the secondary signal at *LITAF*, rs570620219, was located 40 kb away from the lead SNV (rs2080512).

Taken together, across both the replication stage and full dataset GWAS, we identified 32 SNVs (28 lead SNVs + 4 secondary SNVs) in 28 loci for resting Tpe (Figure 3; Table 1), which explained 3.20% of its variance. This corresponds to ~21% of its estimated heritability.

Three Genetic Loci Are Associated with the Tpe Response to Exercise and Recovery

For Tpe response to exercise and to recovery traits, no genome-wide significant loci were found in the discovery cohorts. Four and seven variants for each Tpe response trait, respectively, met our pre-defined threshold of $p < 10^{-6}$ to take forward into replication in ~22,000 independent samples. One of the selected SNVs for Tpe response to exercise formally replicated ($p \leq 0.05/4 = 0.0125$) and had concordant directions of effect in discovery and replication datasets (Table 2). None of the seven SNVs for Tpe response to recovery that were taken forward into replication formally replicated (Table 3).

One additional SNV reached genome-wide significance in the full dataset GWAS for each Tpe response trait, all with concordant directions of effect across discovery and replication datasets (Tables 2 and 3). Manhattan plots for the full dataset GWAS results are shown in Figures S4B and S4C, and QQ plots including the discovery (blue) and full dataset (black) GWASs are shown in Figure S5, where the value of the lambdas suggests there was minimal inflation. Regional plots are shown in Figures S8 and S9. We performed conditional analyses and no independent signals were found at any of the identified loci.

In total, across both the replication stage and full dataset GWAS, we identified two loci for Tpe response to exercise, which explained 0.16% of its variance, and one for Tpe response to recovery, which explained 0.06% of its variance (Figure 3 and Tables 2 and 3). Of note, the one locus identified for Tpe response to recovery did not overlap with resting Tpe interval or Tpe response to exercise (Figure 4).

Four Male-Specific Loci for Resting Tpe and Three for Tpe Response to Exercise and Recovery

We identified variants associated with resting Tpe in males at four additional loci: *FAAP20* (MIM: 615183), *GPR1* (MIM: 600239), *HEY2* (MIM: 604674), and *LIG3* (MIM: 600940). Variants at these loci were not significant ($p > 5 \times 10^{-8}$) in the combined analyses (Table S4A, Figure S10). For Tpe response to exercise, we identified one variant at the *ETS2* locus (MIM: 164740) for males ($n = 24{,}241$). This variant was non-significant in the combined sex GWAS (Table S4B, Figure S11). Finally, for Tpe response to recovery, we identified two female-specific variants at loci *NRXN3* (MIM: 600567) and *NOL4L* (Table S4C, Figure S12).

Table 2. Discovery, Replication, and Full GWAS Results for the Lead SNVs for Tpe Dynamics during Exercise

						Discovery					Replication					Combined				
Locus	SNV	CHR	BP	EA	AA	EAF	β	SE	p	n	EAF	β	SE	p	n	EAF	β	SE	p	n
EIPR1	rs11127417	2	3357993	G	T	0.012	0.154	0.038	4.30E−05	29,389	0.011	0.165	0.045	2.30E−04	21,854	0.012	0.161	0.029	2.40E−08	51,764
KCNJ2[a]	**rs1468572**	**17**	**68411445**	**T**	**C**	**0.781**	**0.051**	**0.010**	**2.20E−07**	**29,010**	**0.781**	**0.048**	**0.012**	**2.70E−05**	**21,572**	**0.780**	**0.050**	**0.007**	**2.70E−11**	**51,096**

Abbreviations: SNV, single-nucleotide variation; CHR, chromosome; BP, position, based on HG build 19; EA, effect allele; AA, alternate allele; EAF, effect allele frequency from discovery data; β, beta; SE, standard error; n, number of participants; p, p value. The locus name indicates the gene that is in the closest proximity to the most associated SNV. Replicated SNV is indicated in bold type.
[a]Lead SNV is in moderate LD (r^2 = 0.56) with the lead SNV for resting Tpe.

Bioinformatics for Resting Tpe Loci

None of the lead variants or their close proxies ($r^2 > 0.8$) for resting Tpe were annotated as missense variants. However, we identified regulatory variants that might affect gene expression levels of their target genes in heart and brain tissue by interrogating publicly available eQTL datasets using GTEx (see Web Resources). Nine lead variants associated with resting Tpe (at *SSBP3* [MIM: 607390], *SGIP1* [MIM: 611540], *NKX2-5* [MIM: 610610], and *LIG3*) were in high LD ($r^2 > 0.8$) with top eQTL variants in cardiac and brain tissue (Table S5). We found strong support for pairwise colocalization of SNVs in GWAS with eQTLs at five genes (*SSBP3*, *SGIP1, IGF1R, LITAF*, and *LIG3*) in cardiac left ventricle, three genes (*SSBP3*, *NDRG4* [MIM: 614463], and *LIG3*) in cardiac atrial appendages and three genes (*NKX2-5, RP11-481J2.2*, and *LIG3*) in brain tissue (Table S5).

We next identified 34 potential target genes at 15 resting Tpe loci whose promoter regions form significant chromatin interactions in brain and heart using publicly available Hi-C data (Table S6A).

These results were used to prepare a list of potential candidate genes for each identified locus for resting Tpe (Table S7A).

Enriched Tissues, Gene Sets, and Pathways for Resting Tpe Loci

We observed a significant enrichment of resting Tpe loci in heart tissue (Figure S13). By considering all identified loci, our DEPICT analyses identified enrichment of expression in the heart, in the ventricles and in the atria, with the greatest enrichment in the heart ($p = 1.87 \times 10^{-4}$, false discovery rate < 0.01, Table S8). We also observed significant enrichments (a false discovery rate < 0.05) in 17 gene sets from the Gene Ontology, 15 from the Mouse Phenotype Ontology, 54 from ENSEMBL, and 3 from Kyoto encyclopedia of genes and genomes. The most significant enrichments were negative regulation of transport ($p = 2.26 \times 10^{-6}$) from the Gene Ontology, increased infarction size ($p = 4.04 \times 10^{-6}$) from the Mouse Phenotype Ontology, the NOS3 PPI subnetwork ($p = 2.62 \times 10^{-9}$) from ENSEMBL, and regulation of actin cytoskeleton ($p = 1 \times 10^{-4}$) from Kyoto encyclopedia of genes and genomes (Tables S9A–S9D).

Bioinformatics Analyses of the Tpe Response to Exercise and Recovery Loci

None of the lead variants for Tpe response to exercise or to recovery or their close proxies ($r^2 > 0.8$) were annotated as missense variants or were identified as regulatory variants that might affect gene expression levels of their target genes in heart and brain tissue.

We identified the genes *ETS2* for Tpe response to exercise and *KIK3B* (MIM: 603754) for Tpe response to recovery whose promoter regions formed significant chromatin interactions with them in the left ventricle

Table 3. Discovery, Replication, and Full GWAS Results for the Lead SNV for Tpe Dynamics during Recovery

						Discovery					Replication					Combined				
Locus	SNV	CHR	BP	EA	AA	EAF	β	SE	p	n	EAF	β	SE	p	n	EAF	β	SE	p	n
NAF1	rs150100144	4	163978319	G	AA	0.962	0.109	0.024	3.40E−06	23,300	0.961	0.088	0.031	4.49E−03	17,206	0.962	0.101	0.018	1.10E−08	41,090

Abbreviations: SNV, single-nucleotide variation; CHR, chromosome; BP, position, based on HG build 19; EA, effect allele; AA, alternate allele; EAF, effect allele frequency from discovery data; β, beta; SE, standard error; n, number of participants; p, p value. The locus name indicates the gene that is in the closest proximity to the most associated SNV.

(Tables S6B and S6C). There were no significant results from DEPICT analyses, so we performed pathway analyses using g:profiler[38] including only nearest genes or candidate genes indicated from long-range interaction results (Tables S7B and S7C). The top enriched pathways for Tpe response to exercise were regulation of skeletal muscle contraction by action potential ($p = 3.77 \times 10^{-2}$) and regulation of skeletal muscle contraction via regulation of action potential ($p = 3.77 \times 10^{-2}$, Figure S14). We did not observe any significant biological process for the candidate genes for Tpe response to recovery.

Association of Resting Tpe and Tpe Response to Exercise and to Recovery Loci with Other Traits

SNVs at 13 loci for resting Tpe had previously been associated ($p < 5 \times 10^{-8}$) with other traits, including pulse rate, P-wave duration, resting heart rate (MIM: 607276), QT interval, QRS duration, cardiomegaly, Brugada syndrome (MIM: 601144), and atrial fibrillation (MIM: 608583, Table S10). Variants at two loci (*SSBP3* and *DPT*) for the PR interval, at four loci (*KCND3* [MIM: 605411], *MEF2D* [MIM: 600663], *CAMK2D* [MIM: 607708], and *LITAF*) and at five loci (*SSBP3*, *SCN5A-SCN10A*, *CAMK2D*, *KCNH2* [MIM: 152427], and *KCNJ2* [MIM: 600681]) for the T-wave morphology restitution were genome-wide significant in our results (Table S11).

An overview of loci for resting Tpe and Tpe response to exercise and to recovery with other ECG traits is indicated by a Venn diagram in Figure 4. Interestingly, the loci for Tpe response to exercise and to recovery did not overlap with other ECG traits, except for the *KCNJ2* locus associated with Tpe response to exercise. This locus has also been associated with resting Tpe. It should be noted, however, that both lead SNVs were not in high LD ($r^2 = 0.56$, Tables 1 and 2).

Genetic Risk Score for Resting Tpe

The optimal p value cut-off in the training set was $p = 0.012$ (Figure S15, 12,107 SNVs). The GRS was not significantly associated with arrhythmic events in the validation subset ($p = 0.13$, Figure S15).

Discussion

This is the largest study to date studying the genetic contribution to the Tpe interval, and Tpe response to exercise and recovery. With the unique combination of a robust framework, including independent discovery and replication samples, and dense genetic imputation in ~72,000 individuals,[25] we identified 28 loci and 4 male-specific loci for resting Tpe, 10 of which are specific to resting Tpe. We also identified three loci for Tpe response to exercise (one male-specific locus) and three loci for Tpe response to recovery (two female-specific loci). One locus (*KCNJ2*) for the Tpe response to exercise had previously been

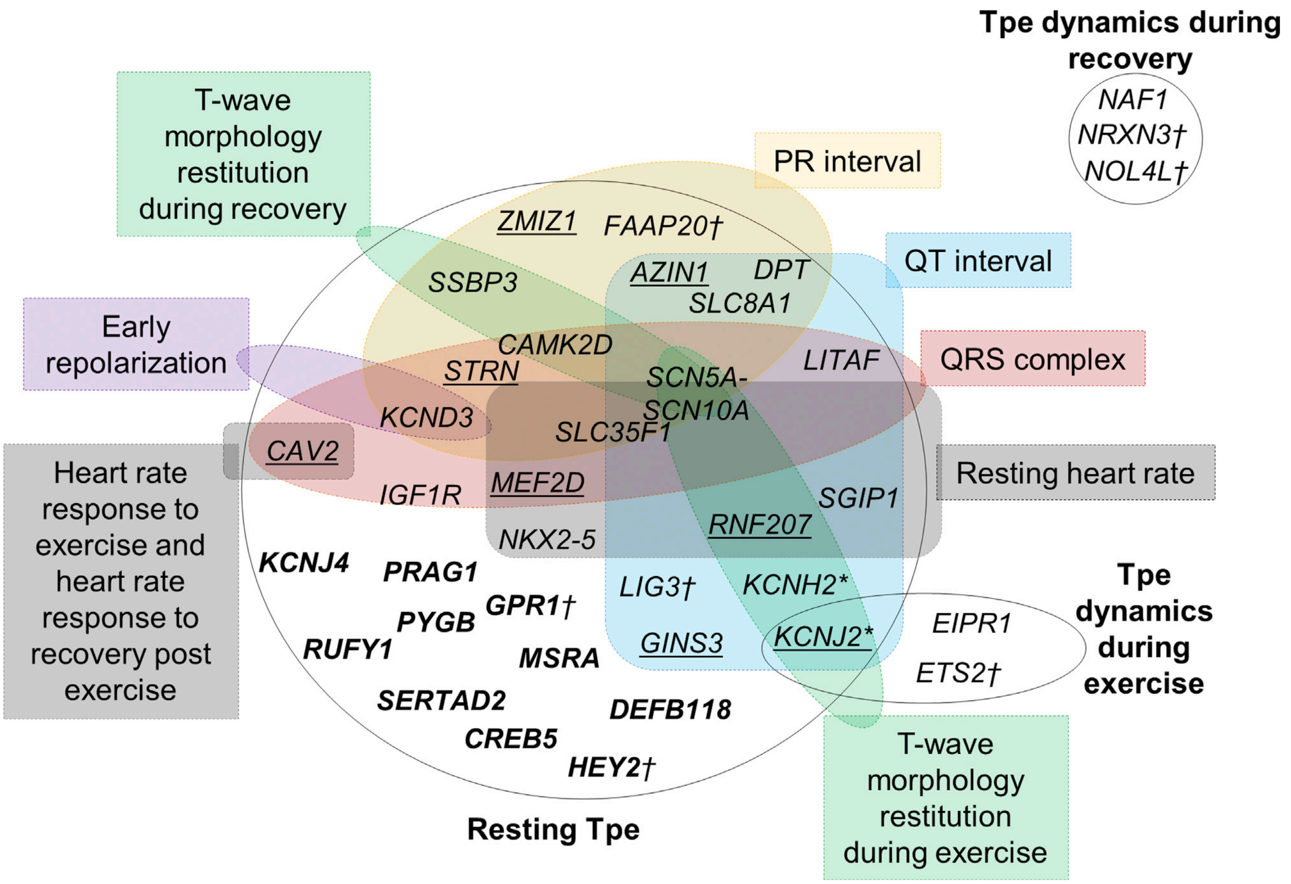

Figure 4. Overlap of Resting Tpe, Tpe Response to Exercise, and Tpe Response to Recovery Loci with Other Electrocardiogram Traits
SNVs at loci with a known genome-wide significant association (from PhenoScanner or GWAS catalog) with other ECG traits are grouped accordingly. The locus names indicate the nearest coding genes. The *KCNJ2* locus was shared between resting Tpe and Tpe dynamics during exercise. There was no loci overlap between Tpe response to recovery and resting Tpe or Tpe response to exercise. There was a substantial number of loci for resting Tpe that did not overlap with other ECG traits. Underlined loci are loci that have previously been associated with other ECG markers but the reported variant was not in high LD ($r^2 < 0.8$) with our lead variant, so potentially independent signals at those loci. Bold loci are loci that have not been associated with other ECG marker.
†Indicates sex-specific loci.
*Indicates previously associated with Tpe interval in other studies (PMID: 20215044 and 22342860).

associated with other ECG traits including resting Tpe. The main biological processes indicated for resting Tpe involved ventricular repolarization and cardiac conduction and contraction.

Of the total 32 loci discovered in this work, 10 (4 validated, 4 identified in the full dataset GWAS, and 2 male-specific) did not overlap with any locus previously reported for another ECG trait (*PRAG1* [MIM: 617344], *PYGB* [MIM: 138550], *CREB5* [MIM: 618262], *KCNJ4* [MIM: 600504], *MSRA*, *RUFY1* [MIM: 610327], *SERTAD2* [MIM: 617851], *DEFB118* [MIM: 607650], *GPR1*, and *HEY2*, respectively; Figure 4). Of the remaining loci, 12 (11 lead and 1 male-specific) were associated with resting QT interval. Two additional loci were associated with resting heart rate, five with QRS complex and three (two lead and one male-specific) with PR interval (Figure 4). These observations underline, as expected, shared genetics among ECG traits, but importantly we also observed specific Tpe loci.

Of the ten resting Tpe-specific loci, a summation of bioinformatics analyses and literature review indicated eight loci (*PRAG1*, *PYGB*, *KCNJ4*, *MSRA*, *RUFY1*, *SERTAD2*, *GPR1*, and *HEY2*) had plausible candidate genes (*PPP1R3B/MFHAS1* [MIM: 610541/605352], *PYGB*, *KCNJ4*, *GATA4* [MIM: 600576], *RUFY1*, *SERTAD2*, *GPR1/ZDBF2* [MIM: 617059], and *HEY2*; Table S7A). From the candidate genes at validated loci, *PYGB* encodes a glycogen phosphorylase (GP) that is found in the heart. The physiological role of myocardial GP is to provide the energy supply required for myocardial contraction and it is associated with diseases including myocardial infarction (MIM: 608446).[46] A second candidate gene, *KCNJ4*, functions closely with *KCNJ2* (also identified in this work). Both genes encode the human inward rectifier potassium channels Kir2.1 and Kir2.3. These potassium selective ion channels determine the resting membrane potential and terminal repolarization of the cardiac action potential. Importantly, mutations in *KCNJ4* are associated with electrolyte imbalance and dilated cardiomyopathy (MIM: 115200).[47] From the candidate genes identified from the full dataset GWAS, *GATA4*, a candidate gene at locus *MSRA*, plays a key role

in cardiac development and function.[48] In co-operation with *TBX5* (MIM: 601620), it binds to cardiac super-enhancers and promotes cardiomyocyte gene expression, while it downregulates endocardial and endothelial gene expression.[48] Mutations in this gene have been associated with cardiac septal defects,[49,50] tetralogy of Fallot (MIM: 187500),[51] cardiac myocyte enlargement,[52] and atrial fibrillation.[53] Finally, from the candidate genes identified in sex-specific analyses, *HEY2* encodes a member of the hairy and enhancer of split-related family of transcription factors. Two similar and redundant genes in the mouse are required for embryonic cardiovascular development. Interestingly, the lead variant we have identified at this locus, rs10457469, is in high LD ($r^2 = 0.97$) with rs9388451, which has been reported to be associated with Brugada syndrome through a *HEY2*-dependent alteration of ion channel expression across the cardiac ventricular wall.[54] Interestingly, *HEY2* represses transcription by the cardiac transcriptional activators *GATA4* and *GATA6* (MIM: 601656).[55]

Bioinformatics analyses on all loci identified in this study indicate that the main biological mechanism underlying resting Tpe is predominantly driven by cellular processes that control ventricular repolarization. As highlighted above, *KCNJ2* and *KCNJ4* are resting Tpe-specific candidate genes involved in ventricular repolarization. In particular, the SNV rs4399570, mapping to *KCNJ2,* demonstrated the strongest association with resting Tpe ($p = 5.30 \times 10^{-143}$) and has one of the largest effect sizes for this trait (1.30 ms). Mutations in *KCNJ2* are associated with short QT syndrome 3 (MIM: 609622)[56] and cardiac arrhythmias.[57,58] In addition, we identified variants at *KCNH2* and *RNF207* (MIM: 616923), both loci previously associated with the QT interval.[59] *KCNH2* is another important gene that encodes a crucial potassium repolarizing current, *HERG*. Finally, *RNF207*, a RING finger protein, is a known modulator of cardiac repolarization through actions on *HERG*.[60] These four loci were validated in our work.

An additional biological mechanism underlying resting Tpe is cardiac conduction and contraction. Several candidate genes, such as *PYGB*, *GATA4*, and *HEY2* (highlighted before), as well as previously reported *SCN5A-SCN10A, CAMK2D*, and *KCND3* are involved. *CAMK2D* is the candidate gene at the validated locus *CAMK2D* and is a calcium/calmodulin-dependent protein kinase involved in the excitation-contraction coupling in heart by targeting Ca(2+) influx into the myocyte. *KCND3* is the candidate gene at locus *KCND3*, discovered in the full dataset GWAS, and encodes the Ito carrying KV4.3 channel, and gain-of-function mutations have been associated with Brugada syndrome[61] and atrial fibrillation.[62] Recent studies have suggested that an increased KV4.3 expression modulates NaV1.5 sodium current, resulting in a loss of conduction.[63] A possible biological mechanism linking ventricular repolarization and cardiac contraction is cardiac mechano-electric coupling, by which myocardial deformation causes changes in cardiac electrophysiological parameters[64,65] and mechanosensitive ion channels modulate ventricular repolarization during ventricular contraction.

Our work significantly expands previous literature on the genetic architecture of the Tpe interval. A previous study[21] on this topic examined the relationship of seven SNVs previously associated with the QT interval to the Tpe interval in 5,890 individuals, two SNVs at *KCNH2* were genome-wide significant in our results (Table S1). The second study[22] performed a GWAS for resting Tpe interval on 1,870 individuals. They discovered and validated a strong signal ($p = 1.1 \times 10^{-10}$), at *KCNJ2*, a locus that was also highly significant in our results ($p = 4.2 \times 10^{-148}$). However, their reported suggestive SNV, rs17749681, at *GRIN2A* (MIM: 138253), was non-significant in our results (Table S1).

The identified loci for Tpe response to exercise and Tpe response to recovery are potentially interesting as there was almost no overlap between traits, with resting Tpe or with other ECG traits. To highlight one of the candidate genes for Tpe response to exercise, *ETS2*, mapping the male-specific locus *ETS2*, plays an important role in a genetic network that governs cardiopoiesis.[66] It has been shown that variations in *ETS2* abundance in hearts of adult rodents and the associated loss of cardiomyocytes contribute to the longevity variability observed during normal aging of rats through activation of programmed necrosis.[67] In the development of a functional myocardium and formation of the coronary vasculature, epicardially derived cells play an essential role, and *ETS2* was found to be essential for normal coronary and myocardial development in chicken embryos.[68]

In this study, the number of identified SNVs for Tpe response to exercise and to recovery was limited, and this might be partly due to the low heritability of the traits (2.2% for Tpe response during exercise and 2.4% for Tpe response during recovery). Our data suggest there is a significant genetic contribution to resting Tpe, but its response to heart rate changes is mainly influenced by environmental factors. This is a general feature that is emerging from our studies, namely that the heritability of exercise-induced changes in cardiac electrophysiology is lower than those at rest.[43,69] Interventions such as exercise training may therefore have an impact on ventricular repolarization and, thus, reduce its associated risk.

Our sex-specific findings strengthen previous studies concluding that there are sex differences in the resting Tpe and its response to heart rate.[13,70–72] Therefore, genetics might be playing a role in the modulation of cardiac electrical activity in addition to sex hormones, with men having a greater genetic influence compared to women.

Despite finding a significant association between the GRS and ventricular arrhythmic events in a training cohort, this significance was not validated in an independent subset of individuals. This might indicate that the common variants modulating resting Tpe do not

contribute to the pathophysiological mechanisms influencing ventricular arrhythmic risk. Alternatively, given the low incidence of arrhythmic events in the UK Biobank, which comprises a relatively healthy population, the validation analysis might have been underpowered. Future studies should evaluate the prognostic value of the GRS in well-powered cohorts for validation of our negative results.

Our study has some limitations. First, we report results from GWASs including all available samples, which indicate seven loci for resting Tpe, two for Tpe response to exercise, and three loci for Tpe response to recovery with no independent replication, so these loci should be considered as preliminary until they are externally validated. Next, due to the relatively low sample size, we restricted our analysis to common variants (MAF $> 1\%$), so we are unable to comment on the role of rare variants on the Tpe traits. In addition, we only report results for European ancestry as this was by far the largest ancestral group in the UKB cohort. Additional studies will need to investigate whether the findings can be extrapolated to other ancestries. Finally, the range of variation of the Tpe response to exercise and recovery traits is small, and the limited sampling rate of the ECG recordings (500 Hz), corresponding to a temporal resolution of 2 ms, might have hindered the resolution of these measurements.

In summary, our findings provide additional loci for Tpe interval traits and reveal the role of ventricular repolarization and cardiac conduction and contraction in modulating them. Our work may guide future studies identifying new therapeutic targets to modulate resting Tpe and its dynamics to prevent and treat ventricular arrhythmias.

Supplemental Data

Supplemental Data can be found online at https://doi.org/10.1016/j.ajhg.2020.04.009.

Acknowledgments

This research is supported by Medical Research Council grant MR/N025083/1. J.R. acknowledges support from the European Union's Horizon 2020 research and innovation program under the Marie Sklodowska-Curie grant agreement no 786833. We also wish to acknowledge the National Institutes of Health Research (NIHR) Cardiovascular Biomedical Centre at Barts and The London, Queen Mary University of London. P.D.L. acknowledges support from the UCLH Biomedicine NIHR, Barts Heart Centre BRC. W.J.Y. is funded by the Medical Research Council (grant code MR/R017468/1). This research utilized Queen Mary's Apocrita HPC facility, supported by QMUL Research-IT (https://doi.org/10.5281/zenodo.438045).

Declaration of Interests

The authors declare no competing interests.

Received: November 5, 2019
Accepted: April 8, 2020
Published: May 7, 2020

Web Resources

1000 Genomes reference panel, https://www.internationalgenome.org/category/reference/

Baseline wonder removal algorithm, https://github.com/Tereshchenkolab/S-ICD_eligibility

GTEx Portal, https://gtexportal.org/home/

GWAS Catalog, https://www.ebi.ac.uk/gwas/

Haplotype Reference Consortium, http://www.haplotype-reference-consortium.org/site

OMIM, https://www.omim.org/

PhenoScanner, http://www.phenoscanner.medschl.cam.ac.uk/

UK Biobank, https://www.ukbiobank.ac.uk

References

1. Wong, C.X., Brown, A., Lau, D.H., Chugh, S.S., Albert, C.M., Kalman, J.M., and Sanders, P. (2019). Epidemiology of Sudden Cardiac Death: Global and Regional Perspectives. Heart Lung Circ. *28*, 6–14.
2. Hayashi, M., Shimizu, W., and Albert, C.M. (2015). The spectrum of epidemiology underlying sudden cardiac death. Circ. Res. *116*, 1887–1906.
3. Myerburg, R.J., Reddy, V., and Castellanos, A. (2009). Indications for implantable cardioverter-defibrillators based on evidence and judgment. J. Am. Coll. Cardiol. *54*, 747–763.
4. Panikkath, R., Reinier, K., Uy-Evanado, A., Teodorescu, C., Hattenhauer, J., Mariani, R., Gunson, K., Jui, J., and Chugh, S.S. (2011). Prolonged Tpeak-to-tend interval on the resting ECG is associated with increased risk of sudden cardiac death. Circ Arrhythm Electrophysiol *4*, 441–447.
5. Yamaguchi, M., Shimizu, M., Ino, H., Terai, H., Uchiyama, K., Oe, K., Mabuchi, T., Konno, T., Kaneda, T., and Mabuchi, H. (2003). T wave peak-to-end interval and QT dispersion in acquired long QT syndrome: a new index for arrhythmogenicity. Clin. Sci. (Lond.) *105*, 671–676.
6. Tse, G., Gong, M., Meng, L., Wong, C.W., Bazoukis, G., Chan, M.T.V., Wong, M.C.S., Letsas, K.P., Baranchuk, A., Yan, G.-X., et al. (2018). Predictive Value of Tpeak – Tend Indices for Adverse Outcomes in Acquired QT Prolongation: A Meta-Analysis. Front. Physiol. *9*, 1226.
7. Tse, G., Gong, M., Li, C.K.H., Leung, K.S.K., Georgopoulos, S., Bazoukis, G., Letsas, K.P., Sawant, A.C., Mugnai, G., Wong, M.C.S., et al.; International Health Informatics Study (IHIS) Network (2018). T_{peak}-T_{end}, T_{peak}-T_{end}/QT ratio and T_{peak}-T_{end} dispersion for risk stratification in Brugada Syndrome: A systematic review and meta-analysis. J. Arrhythm. *34*, 587–597.
8. Letsas, K.P., Weber, R., Astheimer, K., Kalusche, D., and Arentz, T. (2010). Tpeak-Tend interval and Tpeak-Tend/QT ratio as markers of ventricular tachycardia inducibility in subjects with Brugada ECG phenotype. Europace *12*, 271–274.
9. Dinshaw, L., Münch, J., Dickow, J., Lezius, S., Willems, S., Hoffmann, B.A., and Patten, M. (2018). The T-peak-to-T-end interval: a novel ECG marker for ventricular arrhythmia and appropriate ICD therapy in patients with hypertrophic cardiomyopathy. Clin. Res. Cardiol. *107*, 130–137.

10. Watanabe, N., Kobayashi, Y., Tanno, K., Miyoshi, F., Asano, T., Kawamura, M., Mikami, Y., Adachi, T., Ryu, S., Miyata, A., and Katagiri, T. (2004). Transmural dispersion of repolarization and ventricular tachyarrhythmias. J. Electrocardiol. *37*, 191–200.
11. Rosenthal, T.M., Stahls, P.F., 3rd, Abi Samra, F.M., Bernard, M.L., Khatib, S., Polin, G.M., Xue, J.Q., and Morin, D.P. (2015). T-peak to T-end interval for prediction of ventricular tachyarrhythmia and mortality in a primary prevention population with systolic cardiomyopathy. Heart Rhythm *12*, 1789–1797.
12. Vehmeijer, J.T., Koyak, Z., Vink, A.S., Budts, W., Harris, L., Silversides, C.K., Oechslin, E.N., Zwinderman, A.H., Mulder, B.J.M., and de Groot, J.R. (2019). Prolonged T_{peak} -T_{end} interval is a risk factor for sudden cardiac death in adults with congenital heart disease. Congenit. Heart Dis. *14*, 952–957.
13. Ramírez, J., Laguna, P., Bayés de Luna, A., Malik, M., and Pueyo, E. (2014). QT/RR and T-peak-to-end/RR curvatures and slopes in chronic heart failure: relation to sudden cardiac death. J. Electrocardiol. *47*, 842–848.
14. Ramírez, J., Monasterio, V., Mincholé, A., Llamedo, M., Lenis, G., Cygankiewicz, I., Bayés de Luna, A., Malik, M., Martínez, J.P., Laguna, P., and Pueyo, E. (2015). Automatic SVM classification of sudden cardiac death and pump failure death from autonomic and repolarization ECG markers. J. Electrocardiol. *48*, 551–557.
15. Antzelevitch, C., Sicouri, S., Di Diego, J.M., Burashnikov, A., Viskin, S., Shimizu, W., Yan, G.-X., Kowey, P., and Zhang, L. (2007). Does Tpeak-Tend provide an index of transmural dispersion of repolarization? Heart Rhythm *4*, 1114–1116, author reply 1116–1119.
16. Srinivasan, N.T., Orini, M., Providencia, R., Simon, R., Lowe, M., Segal, O.R., Chow, A.W., Schilling, R.J., Hunter, R.J., Taggart, P., et al. (2019). Differences in the Upslope of the Precordial Body Surface ECG T-Wave Reflect Right To Left Dispersion of Repolarization in the Intact Human Heart. Heart Rhythm *16*, 943–951.
17. Malik, M., Huikuri, H.V., Lombardi, F., Schmidt, G., Verrier, R.L., Zabel, M.; and e-Rhythm Group of EHRA (2019). Is the T_{peak}-T_{end} interval as a measure of repolarization heterogeneity dead or just seriously wounded? Heart Rhythm *16*, 952–953.
18. Orini, M., Srinivasan, N., Taggart, P., and Lambiase, P.D. (2018). Evaluation of Multi-Lead ECG Markers to Track Changes in Dispersion of Ventricular Repolarization in the Intact Human Heart. In 2018 Computing in Cardiology Conference (CinC), pp. 1–4.
19. Meijborg, V.M.F., Conrath, C.E., Opthof, T., Belterman, C.N.W., de Bakker, J.M., and Coronel, R. (2014). Electrocardiographic T wave and its relation with ventricular repolarization along major anatomical axes. Circ Arrhythm Electrophysiol *7*, 524–531.
20. Hodkinson, E.C., Neijts, M., Sadrieh, A., Imtiaz, M.S., Baumert, M., Subbiah, R.N., Hayward, C.S., Boomsma, D., Willemsen, G., Vandenberg, J.I., et al. (2016). Heritability of ECG Biomarkers in the Netherlands Twin Registry Measured from Holter ECGs. Front. Physiol. *7*, 154.
21. Porthan, K., Marjamaa, A., Viitasalo, M., Väänänen, H., Jula, A., Toivonen, L., Nieminen, M.S., Newton-Cheh, C., Salomaa, V., Kontula, K., and Oikarinen, L. (2010). Relationship of common candidate gene variants to electrocardiographic T-wave peak to T-wave end interval and T-wave morphology parameters. Heart Rhythm *7*, 898–903.
22. Marjamaa, A., Oikarinen, L., Porthan, K., Ripatti, S., Peloso, G., Noseworthy, P.A., Viitasalo, M., Nieminen, M.S., Toivonen, L., Kontula, K., et al. (2012). A common variant near the KCNJ2 gene is associated with T-peak to T-end interval. Heart Rhythm *9*, 1099–1103.
23. Sudlow, C., Gallacher, J., Allen, N., Beral, V., Burton, P., Danesh, J., Downey, P., Elliott, P., Green, J., Landray, M., et al. (2015). UK biobank: an open access resource for identifying the causes of a wide range of complex diseases of middle and old age. PLoS Med. *12*, e1001779.
24. Bycroft, C., Freeman, C., Petkova, D., Band, G., Elliott, L.T., Sharp, K., Motyer, A., Vukcevic, D., Delaneau, O., O'Connell, J., et al. (2017). Genome-wide genetic data on ~500,000 UK Biobank participants. bioRxiv. https://doi.org/10.1101/166298.
25. Huang, J., Howie, B., McCarthy, S., Memari, Y., Walter, K., Min, J.L., Danecek, P., Malerba, G., Trabetti, E., Zheng, H.-F., et al.; UK10K Consortium (2015). Improved imputation of low-frequency and rare variants using the UK10K haplotype reference panel. Nat. Commun. *6*, 8111.
26. Orini, M., Tinker, A., Munroe, P.B., and Lambiase, P.D. (2017). Long-term intra-individual reproducibility of heart rate dynamics during exercise and recovery in the UK Biobank cohort. PLoS ONE *12*, e0183732.
27. Orini, M., Pueyo, E., Laguna, P., and Bailón, R. (2018). A Time-Varying Nonparametric Methodology for Assessing Changes in QT Variability Unrelated to Heart Rate Variability. IEEE Trans. Biomed. Eng. *65*, 1443–1451.
28. Chang, C.C., Chow, C.C., Tellier, L.C.A.M., Vattikuti, S., Purcell, S.M., and Lee, J.J. (2015). Second-generation PLINK: rising to the challenge of larger and richer datasets. Gigascience *4*, 7.
29. Loh, P.-R., Bhatia, G., Gusev, A., Finucane, H.K., Bulik-Sullivan, B.K., Pollack, S.J., de Candia, T.R., Lee, S.H., Wray, N.R., Kendler, K.S., et al.; Schizophrenia Working Group of Psychiatric Genomics Consortium (2015). Contrasting genetic architectures of schizophrenia and other complex diseases using fast variance-components analysis. Nat. Genet. *47*, 1385–1392.
30. Loh, P.-R., Tucker, G., Bulik-Sullivan, B.K., Vilhjálmsson, B.J., Finucane, H.K., Salem, R.M., Chasman, D.I., Ridker, P.M., Neale, B.M., Berger, B., et al. (2015). Efficient Bayesian mixed-model analysis increases association power in large cohorts. Nat. Genet. *47*, 284–290.
31. Bulik-Sullivan, B.K., Loh, P.-R., Finucane, H.K., Ripke, S., Yang, J., Patterson, N., Daly, M.J., Price, A.L., Neale, B.M.; and Schizophrenia Working Group of the Psychiatric Genomics Consortium (2015). LD Score regression distinguishes confounding from polygenicity in genome-wide association studies. Nat. Genet. *47*, 291–295.
32. Yang, J., Lee, S.H., Goddard, M.E., and Visscher, P.M. (2011). GCTA: a tool for genome-wide complex trait analysis. Am. J. Hum. Genet. *88*, 76–82.
33. Warren, H.R., Evangelou, E., Cabrera, C.P., Gao, H., Ren, M., Mifsud, B., Ntalla, I., Surendran, P., Liu, C., Cook, J.P., et al.; International Consortium of Blood Pressure (ICBP) 1000G Analyses; BIOS Consortium; Lifelines Cohort Study; Understanding Society Scientific group; CHD Exome+ Consortium; ExomeBP Consortium; T2D-GENES Consortium; GoT2DGenes Consortium; Cohorts for Heart and Ageing Research in

Genome Epidemiology (CHARGE) BP Exome Consortium; International Genomics of Blood Pressure (iGEN-BP) Consortium; and UK Biobank CardioMetabolic Consortium BP working group (2017). Genome-wide association analysis identifies novel blood pressure loci and offers biological insights into cardiovascular risk. Nat. Genet. *49*, 403–415.
34. McLaren, W., Gil, L., Hunt, S.E., Riat, H.S., Ritchie, G.R.S., Thormann, A., Flicek, P., and Cunningham, F. (2016). The Ensembl Variant Effect Predictor. Genome Biol. *17*, 122.
35. Giambartolomei, C., Vukcevic, D., Schadt, E.E., Franke, L., Hingorani, A.D., Wallace, C., and Plagnol, V. (2014). Bayesian test for colocalisation between pairs of genetic association studies using summary statistics. PLoS Genet. *10*, e1004383.
36. Boyle, A.P., Hong, E.L., Hariharan, M., Cheng, Y., Schaub, M.A., Kasowski, M., Karczewski, K.J., Park, J., Hitz, B.C., Weng, S., et al. (2012). Annotation of functional variation in personal genomes using RegulomeDB. Genome Res. *22*, 1790–1797.
37. Pers, T.H., Karjalainen, J.M., Chan, Y., Westra, H.-J., Wood, A.R., Yang, J., Lui, J.C., Vedantam, S., Gustafsson, S., Esko, T., et al.; Genetic Investigation of ANthropometric Traits (GIANT) Consortium (2015). Biological interpretation of genome-wide association studies using predicted gene functions. Nat. Commun. *6*, 5890.
38. Raudvere, U., Kolberg, L., Kuzmin, I., Arak, T., Adler, P., Peterson, H., and Vilo, J. (2019). g:Profiler: a web server for functional enrichment analysis and conversions of gene lists (2019 update). Nucleic Acids Res. *47* (W1), W191–W198.
39. Iotchkova, V., Ritchie, G.R.S., Geihs, M., Morganella, S., Min, J.L., Walter, K., Timpson, N., Dunham, I., Birney, E., and Soranzo, N. (2016). GARFIELD - GWAS Analysis of Regulatory or Functional Information Enrichment with LD correction. bioRxiv. https://doi.org/10.1101/085738.
40. Smith, C.L., Blake, J.A., Kadin, J.A., Richardson, J.E., Bult, C.J.; and Mouse Genome Database Group (2018). Mouse Genome Database (MGD)-2018: knowledgebase for the laboratory mouse. Nucleic Acids Res. *46* (D1), D836–D842.
41. Staley, J.R., Blackshaw, J., Kamat, M.A., Ellis, S., Surendran, P., Sun, B.B., Paul, D.S., Freitag, D., Burgess, S., Danesh, J., et al. (2016). PhenoScanner: a database of human genotype-phenotype associations. Bioinformatics *32*, 3207–3209.
42. Ntalla, I., Weng, L.-C., Cartwright, J.H., Hall, A.W., Sveinbjornsson, G., Tucker, N.R., Choi, S.H., Chaffin, M.D., Roselli, C., Barnes, M.R., et al. (2019). Multi-ancestry GWAS of the electrocardiographic PR interval identifies 210 loci underlying cardiac conduction. bioRxiv. https://doi.org/10.1101/712398.
43. Ramírez, J., van Duijvenboden, S., Aung, N., Laguna, P., Pueyo, E., Tinker, A., Lambiase, P.D., Orini, M., and Munroe, P.B. (2019). Cardiovascular Predictive Value and Genetic Basis of Ventricular Repolarization Dynamics. Circ Arrhythm Electrophysiol *12*, e007549.
44. Norland, K., Sveinbjornsson, G., Thorolfsdottir, R.B., Davidsson, O.B., Tragante, V., Rajamani, S., Helgadottir, A., Gretarsdottir, S., van Setten, J., Asselbergs, F.W., et al. (2019). Sequence variants with large effects on cardiac electrophysiology and disease. Nat. Commun. *10*, 4803.
45. Choi, S.W., and O'Reilly, P.F. (2019). PRSice-2: Polygenic Risk Score software for biobank-scale data. Gigascience *8*, 8.
46. Dobric, M., Ostojic, M., Giga, V., Djordjevic-Dikic, A., Stepanovic, J., Radovanovic, N., and Beleslin, B. (2015). Glycogen phosphorylase BB in myocardial infarction. Clin. Chim. Acta *438*, 107–111.
47. Szuts, V., Ménesi, D., Varga-Orvos, Z., Zvara, Á., Houshmand, N., Bitay, M., Bogáts, G., Virág, L., Baczkó, I., Szalontai, B., et al. (2013). Altered expression of genes for Kir ion channels in dilated cardiomyopathy. Can. J. Physiol. Pharmacol. *91*, 648–656.
48. Ang, Y.-S., Rivas, R.N., Ribeiro, A.J.S., Srivas, R., Rivera, J., Stone, N.R., Pratt, K., Mohamed, T.M.A., Fu, J.-D., Spencer, C.I., et al. (2016). Disease Model of GATA4 Mutation Reveals Transcription Factor Cooperativity in Human Cardiogenesis. Cell *167*, 1734–1749.e22.
49. Tomita-Mitchell, A., Maslen, C.L., Morris, C.D., Garg, V., and Goldmuntz, E. (2007). GATA4 sequence variants in patients with congenital heart disease. J. Med. Genet. *44*, 779–783.
50. Wu, G., Shan, J., Pang, S., Wei, X., Zhang, H., and Yan, B. (2012). Genetic analysis of the promoter region of the GATA4 gene in patients with ventricular septal defects. Transl. Res. *159*, 376–382.
51. Yang, Y.-Q., Gharibeh, L., Li, R.-G., Xin, Y.-F., Wang, J., Liu, Z.-M., Qiu, X.-B., Xu, Y.-J., Xu, L., Qu, X.-K., et al. (2013). GATA4 loss-of-function mutations underlie familial tetralogy of fallot. Hum. Mutat. *34*, 1662–1671.
52. Sunagawa, Y., Morimoto, T., Takaya, T., Kaichi, S., Wada, H., Kawamura, T., Fujita, M., Shimatsu, A., Kita, T., and Hasegawa, K. (2010). Cyclin-dependent kinase-9 is a component of the p300/GATA4 complex required for phenylephrine-induced hypertrophy in cardiomyocytes. J. Biol. Chem. *285*, 9556–9568.
53. Nielsen, J.B., Thorolfsdottir, R.B., Fritsche, L.G., Zhou, W., Skov, M.W., Graham, S.E., Herron, T.J., McCarthy, S., Schmidt, E.M., Sveinbjornsson, G., et al. (2018). Biobank-driven genomic discovery yields new insight into atrial fibrillation biology. Nat. Genet. *50*, 1234–1239.
54. Veerman, C.C., Podliesna, S., Tadros, R., Lodder, E.M., Mengarelli, I., de Jonge, B., Beekman, L., Barc, J., Wilders, R., Wilde, A.A.M., et al. (2017). The Brugada Syndrome Susceptibility Gene *HEY2* Modulates Cardiac Transmural Ion Channel Patterning and Electrical Heterogeneity. Circ. Res. *121*, 537–548.
55. Shirvani, S., Xiang, F., Koibuchi, N., and Chin, M.T. (2006). CHF1/Hey2 suppresses SM-MHC promoter activity through an interaction with GATA-6. Biochem. Biophys. Res. Commun. *339*, 151–156.
56. Priori, S.G., Pandit, S.V., Rivolta, I., Berenfeld, O., Ronchetti, E., Dhamoon, A., Napolitano, C., Anumonwo, J., di Barletta, M.R., Gudapakkam, S., et al. (2005). A novel form of short QT syndrome (SQT3) is caused by a mutation in the KCNJ2 gene. Circ. Res. *96*, 800–807.
57. Eckhardt, L.L., Farley, A.L., Rodriguez, E., Ruwaldt, K., Hammill, D., Tester, D.J., Ackerman, M.J., and Makielski, J.C. (2007). KCNJ2 mutations in arrhythmia patients referred for LQT testing: a mutation T305A with novel effect on rectification properties. Heart Rhythm *4*, 323–329.
58. Whittaker, D.G., Ni, H., El Harchi, A., Hancox, J.C., and Zhang, H. (2017). Atrial arrhythmogenicity of KCNJ2 mutations in short QT syndrome: Insights from virtual human atria. PLoS Comput. Biol. *13*, e1005593.
59. Pfeufer, A., Sanna, S., Arking, D.E., Müller, M., Gateva, V., Fuchsberger, C., Ehret, G.B., Orrú, M., Pattaro, C., Köttgen, A., et al. (2009). Common variants at ten loci modulate the QT interval duration in the QTSCD Study. Nat. Genet. *41*, 407–414.

60. Roder, K., Werdich, A.A., Li, W., Liu, M., Kim, T.Y., Organ-Darling, L.E., Moshal, K.S., Hwang, J.M., Lu, Y., Choi, B.-R., et al. (2014). RING finger protein RNF207, a novel regulator of cardiac excitation. J. Biol. Chem. *289*, 33730–33740.

61. van den Berg, M.P., and Bezzina, C.R. (2011). KCND3 mutations in Brugada syndrome: the plot thickens. Heart Rhythm *8*, 1033–1035.

62. Olesen, M.S., Refsgaard, L., Holst, A.G., Larsen, A.P., Grubb, S., Haunsø, S., Svendsen, J.H., Olesen, S.-P., Schmitt, N., and Calloe, K. (2013). A novel KCND3 gain-of-function mutation associated with early-onset of persistent lone atrial fibrillation. Cardiovasc. Res. *98*, 488–495.

63. Portero, V., Wilders, R., Casini, S., Charpentier, F., Verkerk, A.O., and Remme, C.A. (2018). K_V4.3 Expression Modulates Na_V1.5 Sodium Current. Front. Physiol. *9*, 178.

64. Quinn, T.A., Kohl, P., and Ravens, U. (2014). Cardiac mechano-electric coupling research: fifty years of progress and scientific innovation. Prog. Biophys. Mol. Biol. *115*, 71–75.

65. Orini, M., Nanda, A., Yates, M., Di Salvo, C., Roberts, N., Lambiase, P.D., and Taggart, P. (2017). Mechano-electrical feedback in the clinical setting: Current perspectives. Prog. Biophys. Mol. Biol. *130* (Pt B), 365–375.

66. Islas, J.F., Liu, Y., Weng, K.-C., Robertson, M.J., Zhang, S., Prejusa, A., Harger, J., Tikhomirova, D., Chopra, M., Iyer, D., et al. (2012). Transcription factors ETS2 and MESP1 transdifferentiate human dermal fibroblasts into cardiac progenitors. Proc. Natl. Acad. Sci. USA *109*, 13016–13021.

67. Sheydina, A., Volkova, M., Jiang, L., Juhasz, O., Zhang, J., Tae, H.-J., Perino, M.G., Wang, M., Zhu, Y., Lakatta, E.G., and Boheler, K.R. (2012). Linkage of cardiac gene expression profiles and ETS2 with lifespan variability in rats. Aging Cell *11*, 350–359.

68. Lie-Venema, H., Gittenberger-de Groot, A.C., van Empel, L.J., Boot, M.J., Kerkdijk, H., de Kant, E., and DeRuiter, M.C. (2003). Ets-1 and Ets-2 transcription factors are essential for normal coronary and myocardial development in chicken embryos. Circ. Res. *92*, 749–756.

69. Ramírez, J., Duijvenboden, S.V., Ntalla, I., Mifsud, B., Warren, H.R., Tzanis, E., Orini, M., Tinker, A., Lambiase, P.D., and Munroe, P.B. (2018). Thirty loci identified for heart rate response to exercise and recovery implicate autonomic nervous system. Nat. Commun. *9*, 1947.

70. Cheng, J. (2006). Evidences of the gender-related differences in cardiac repolarization and the underlying mechanisms in different animal species and human. Fundam. Clin. Pharmacol. *20*, 1–8.

71. Valverde, E.R., Biagetti, M.O., Bertran, G.R., Arini, P.D., Bidoggia, H., and Quinteiro, R.A. (2003). Developmental changes of cardiac repolarization in rabbits: implications for the role of sex hormones. Cardiovasc. Res. *57*, 625–631.

72. Smetana, P., Batchvarov, V., Hnatkova, K., John Camm, A., and Malik, M. (2003). Sex differences in the rate dependence of the T wave descending limb. Cardiovasc. Res. *58*, 549–554.

The American Journal of Human Genetics, Volume *106*

Supplemental Data

Common Genetic Variants Modulate the Electrocardiographic Tpeak-to-Tend Interval

Julia Ramírez, Stefan van Duijvenboden, William J. Young, Michele Orini, Pier D. Lambiase, Patricia B. Munroe, and Andrew Tinker

Supplemental Figures

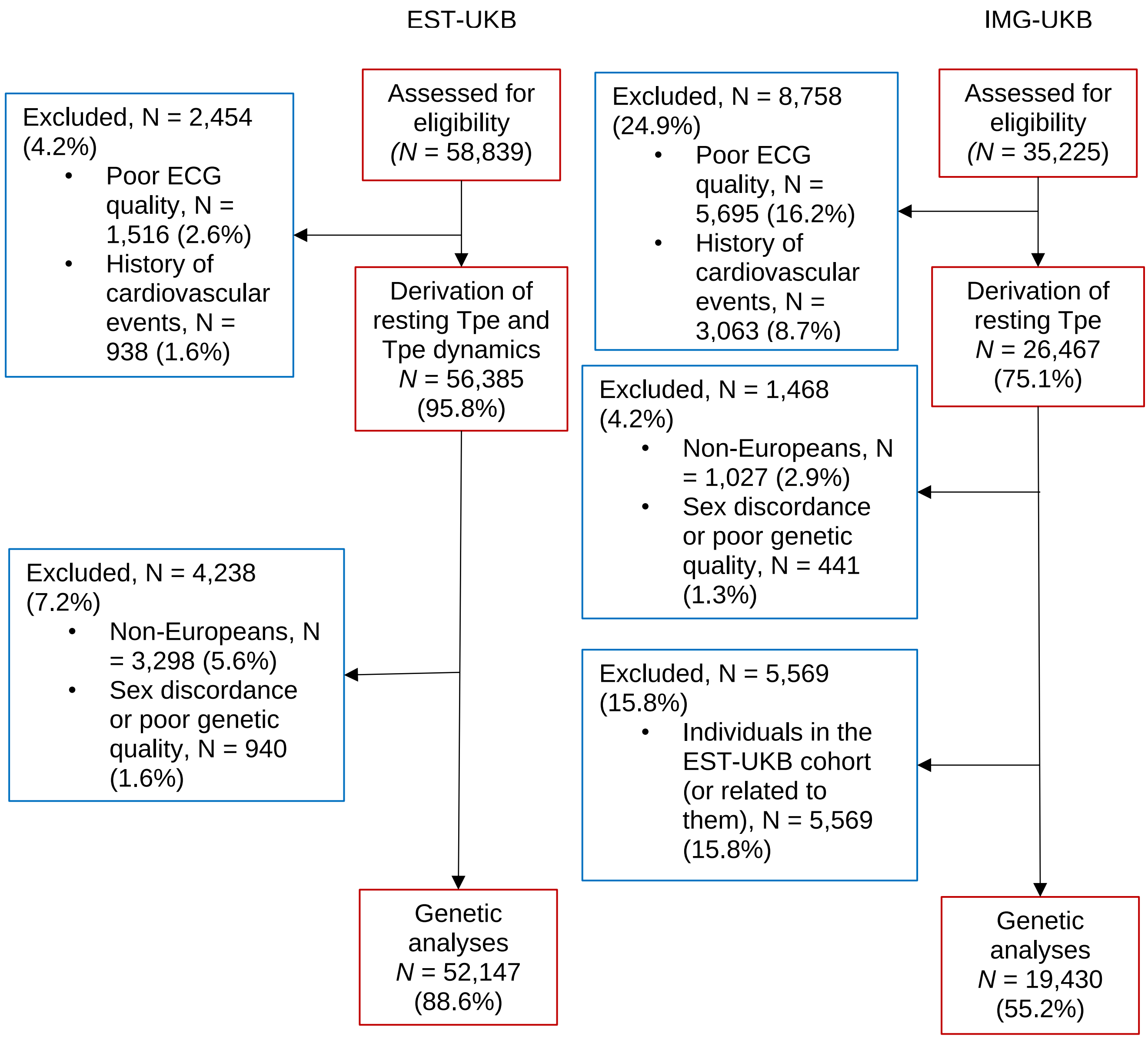

Figure S1: Exercise stress test (EST-UKB) and Imaging (IMG-UKB) study populations flow diagram.

Additional information can be found in Methods.

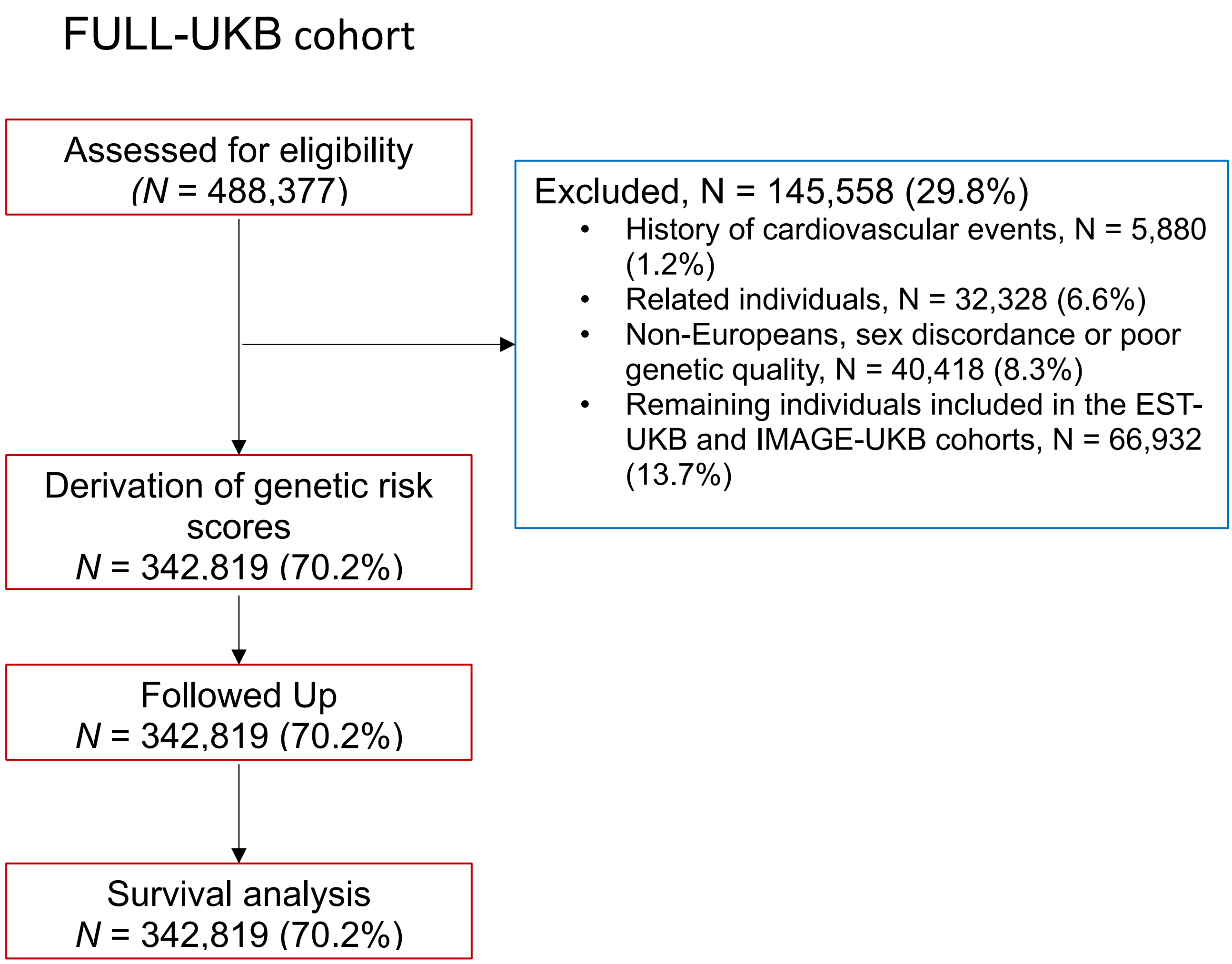

Figure S2: Full cohort (FULL-UKB) study population flow diagram.

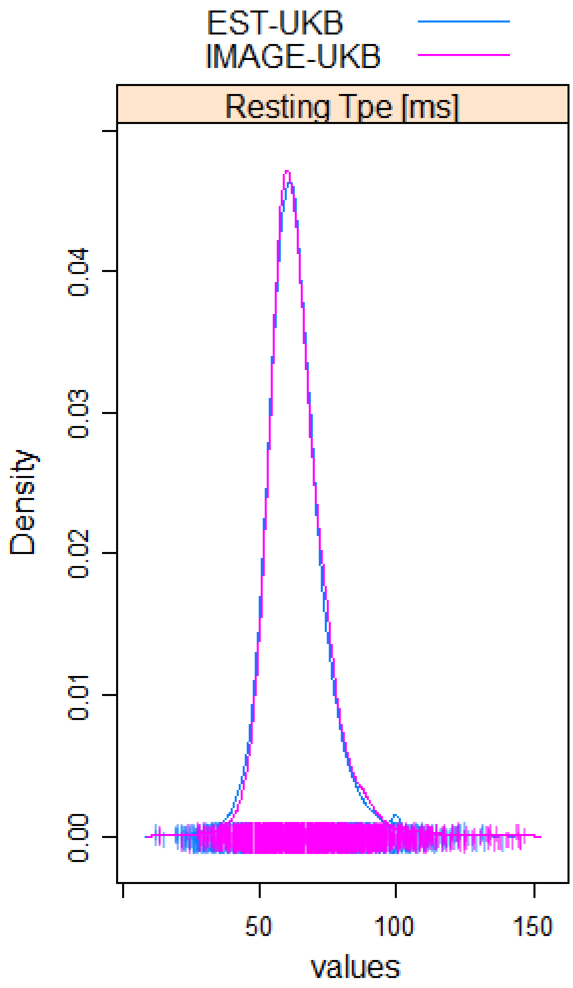

Figure S3: Density plots of resting Tpe intervals in the EST-UKB (blue) and IMAGE-UKB (pink) cohorts.

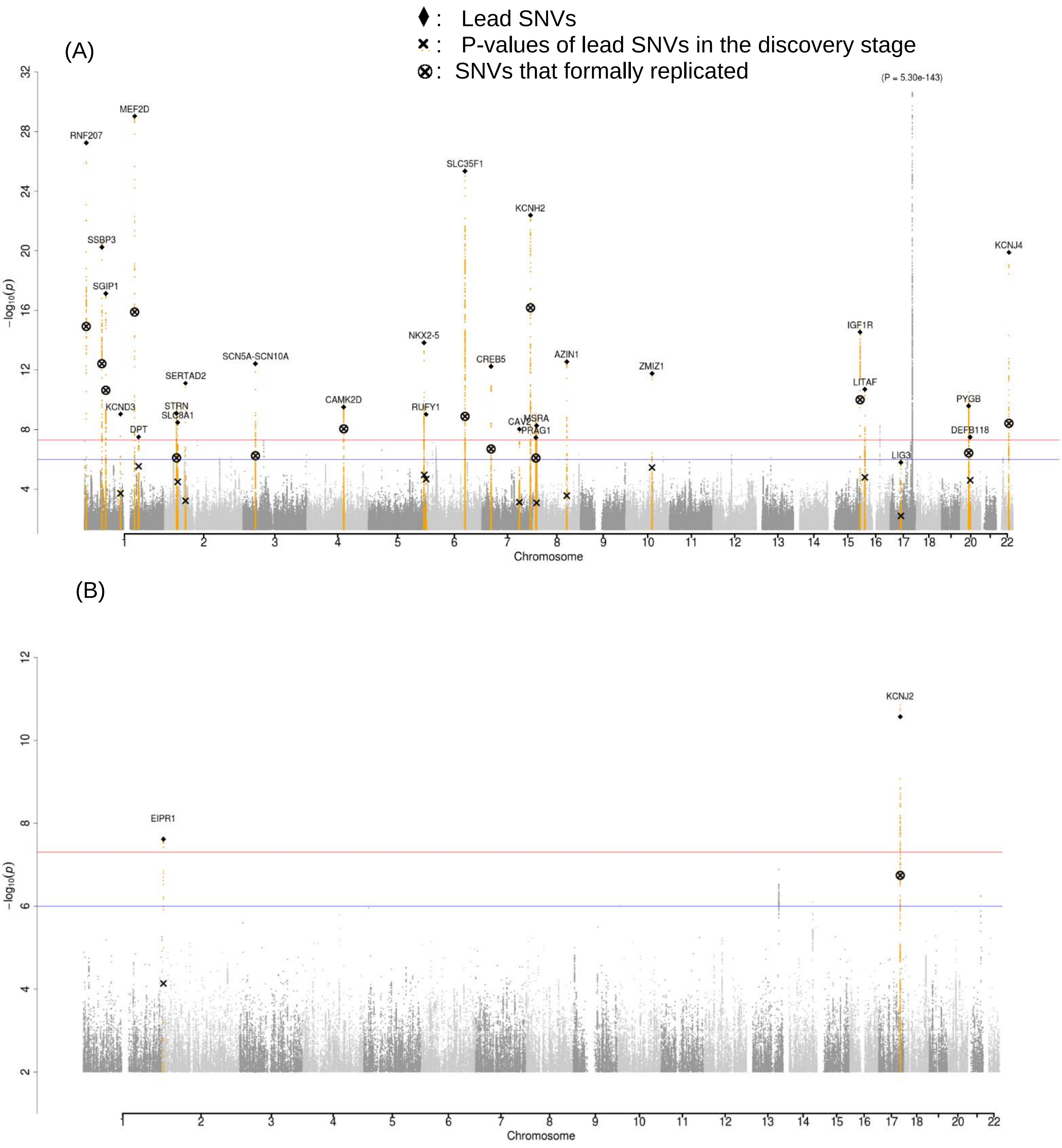

◆ : Lead SNVs
× : P-values of lead SNVs in the discovery stage
⊗ : SNVs that formally replicated
(A)
(P = 5.30e-143)
MEF2D
RNF207
SLC35F1
KCNH2
SSBP3
KCNJ4
SGIP1
IGF1R
NKX2-5
SCN5A-SCN10A
CREB5
AZIN1
ZMIZ1
SERTAD2
LITAF
CAMK2D
PYGB
KCND3
STRN
SLC8A1
RUFY1
CAV2
HSRA
PRAG1
DPT
DEFB118
LIG3
$-\log_{10}(p)$
Chromosome
(B)
KCNJ2
EIPR1
$-\log_{10}(p)$
Chromosome

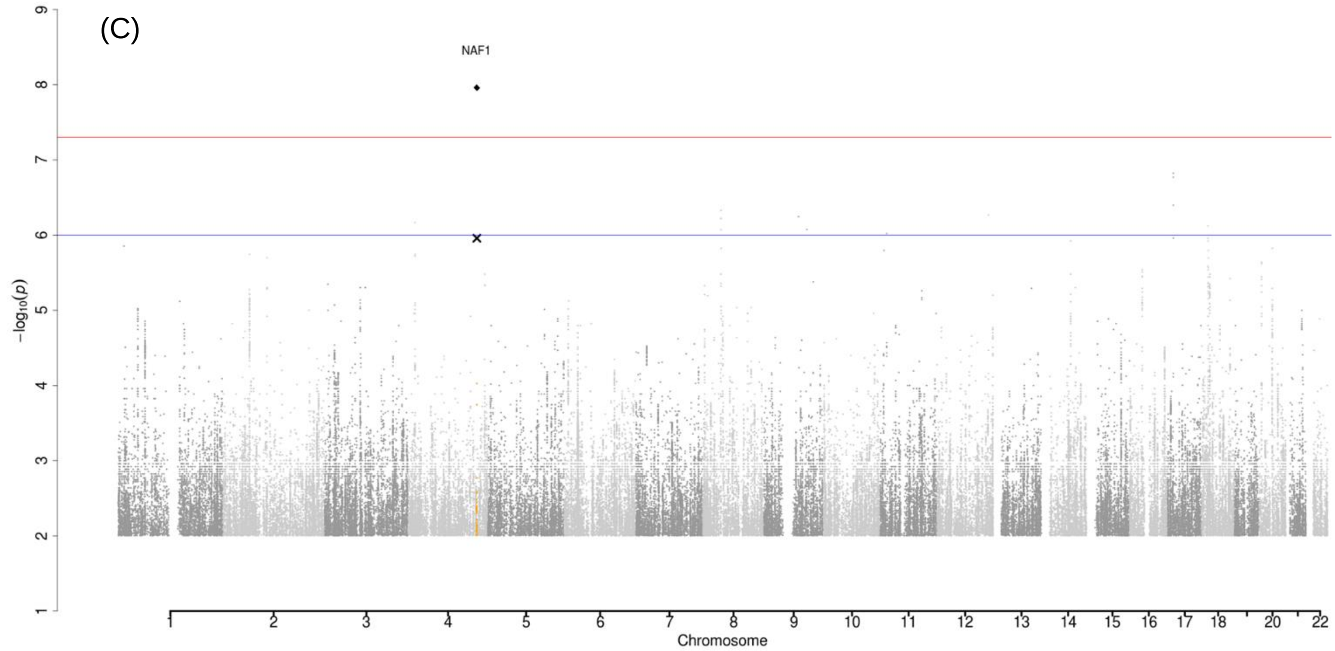

Figure S4: Manhattan plots of Tpe interval (A), Tpe dynamics during exercise (B) and during recovery (C) in the full cohort analysis.

P values, expressed as –log10(*P*), are plotted according to physical genomic locations by chromosome. Lead SNVs are marked by the diamonds. The crosses indicate the *P* values of these SNVs in the discovery data set. Crosses are encircled for SNVs that formally replicated. Locus names of the novel loci correspond to the nearest annotated gene. The blue horizontal line indicates a *P* value threshold of 1×10^{-6}, corresponding to the lookup significance threshold. The red horizontal line indicates a *P*-value threshold of 5×10^{-8}, corresponding to genome-wide significance. Novel loci are highlighted in yellow.

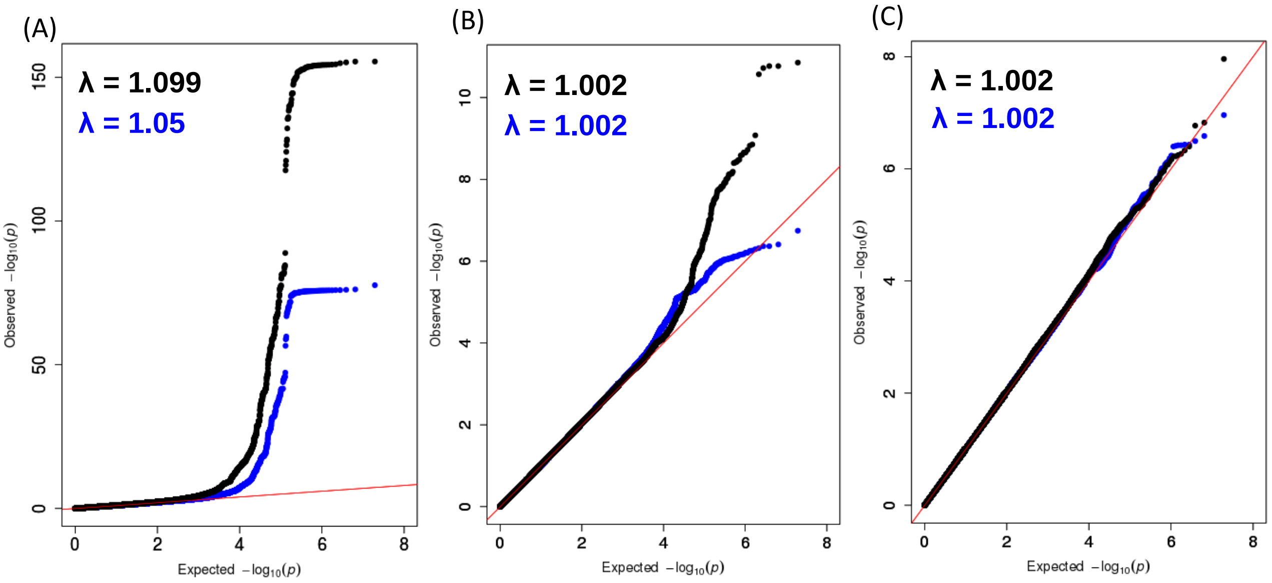

Figure S5: QQ plots for Tpe interval (A), Tpe dynamics during exercise (B) and Tpe dynamics during recovery (C) in the discovery (blue) and full (black) cohorts.

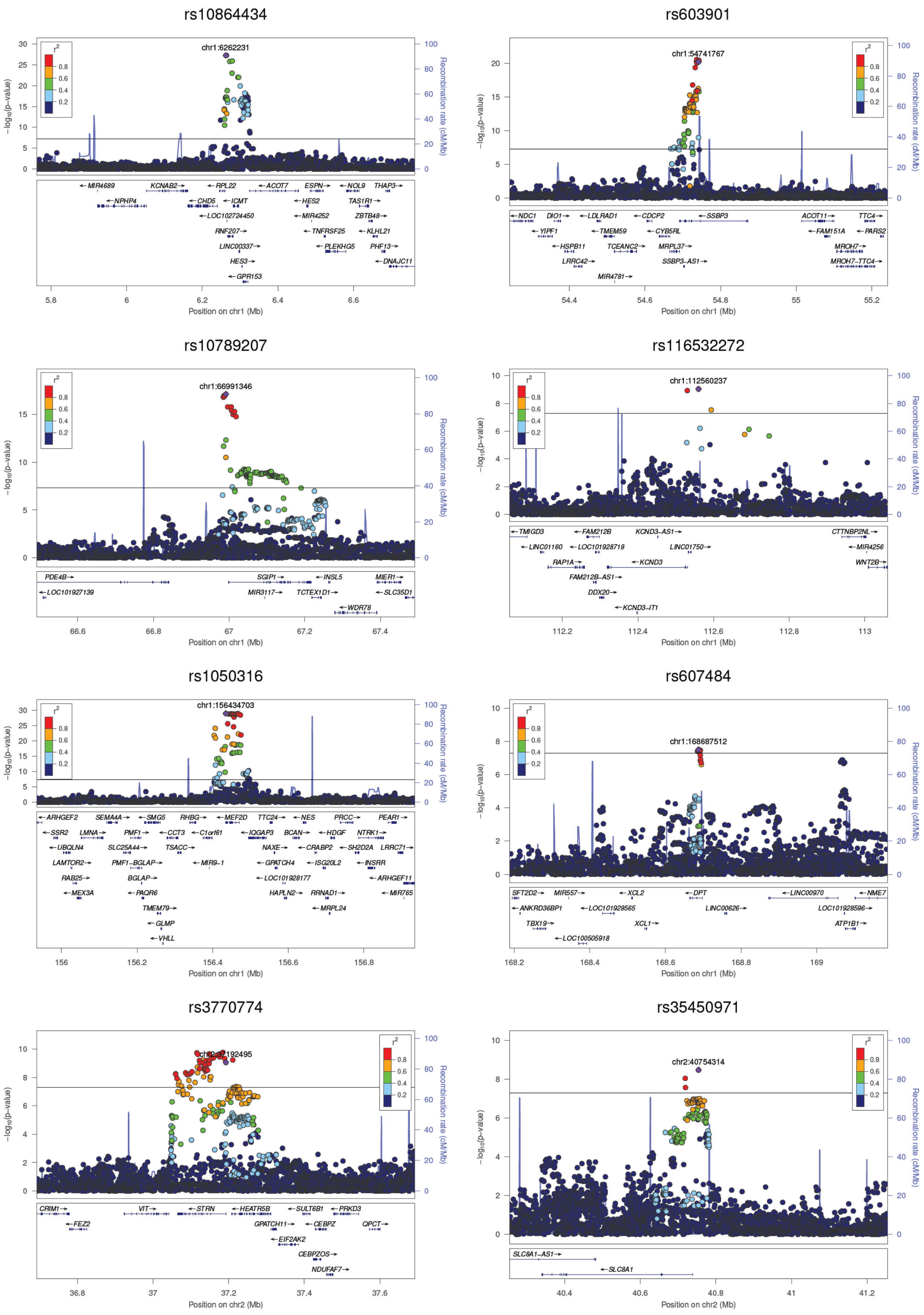
rs10864434
chr1:6262231
−log10(p−value)
Recombination rate (cM/Mb)
Position on chr1 (Mb)
MIR4689
KCNAB2
RPL22
ACOT7
ESPN
NOL9
THAP3
NPHP4
CHD5
ICMT
HES2
TAS1R1
LOC102724450
MIR4252
ZBTB48
RNF207
TNFRSF25
KLHL21
LINC00337
PLEKHG5
PHF13
HES3
DNAJC11
GPR153
rs603901
chr1:54741767
−log10(p−value)
Recombination rate (cM/Mb)
Position on chr1 (Mb)
NDC1
DIO1
LDLRAD1
CDCP2
SSBP3
ACOT11
TTC4
YIPF1
TMEM59
CYB5RL
FAM151A
PARS2
HSPB11
TCEANC2
MRPL37
MROH7
LRRC42
SSBP3−AS1
MROH7−TTC4
MIR4781
rs10789207
chr1:66991346
−log10(p−value)
Recombination rate (cM/Mb)
Position on chr1 (Mb)
PDE4B
SGIP1
INSL5
MIER1
LOC101927139
MIR3117
TCTEX1D1
SLC35D1
WDR78
rs116532272
chr1:112560237
−log10(p−value)
Recombination rate (cM/Mb)
Position on chr1 (Mb)
TMIGD3
FAM212B
KCND3−AS1
CTTNBP2NL
LINC01160
LOC101928718
LINC01750
MIR4256
RAP1A
KCND3
WNT2B
FAM212B−AS1
DDX20
KCND3−IT1
rs1050316
chr1:156434703
−log10(p−value)
Recombination rate (cM/Mb)
Position on chr1 (Mb)
ARHGEF2
SEMA4A
SMG5
RHBG
MEF2D
TTC24
NES
PRCC
PEAR1
SSR2
LMNA
PMF1
CCT3
C1orf61
IQGAP3
BCAN
HDGF
NTRK1
UBQLN4
SLC25A44
TSACC
NAXE
CRABP2
SH2D2A
LRRC71
LAMTOR2
PMF1−BGLAP
MIR9−1
GPATCH4
ISG20L2
INSRR
RAB25
BGLAP
LOC101928177
ARHGEF11
MEX3A
PAQR6
HAPLN2
RRNAD1
MIR765
TMEM79
MRPL24
GLMP
VHLL
rs607484
chr1:168687512
−log10(p−value)
Recombination rate (cM/Mb)
Position on chr1 (Mb)
SFT2D2
MIR557
XCL2
DPT
LINC00970
NME7
ANKRD36BP1
LOC101928565
LINC00626
LOC101928596
TBX19
XCL1
ATP1B1
LOC100505918
rs3770774
chr2:37192495
−log10(p−value)
Recombination rate (cM/Mb)
Position on chr2 (Mb)
CRIM1
VIT
STRN
HEATR5B
SULT6B1
PRKD3
FEZ2
GPATCH11
CEBPZ
QPCT
EIF2AK2
CEBPZOS
NDUFAF7
rs35450971
chr2:40754314
−log10(p−value)
Recombination rate (cM/Mb)
Position on chr2 (Mb)
SLC8A1−AS1
SLC8A1

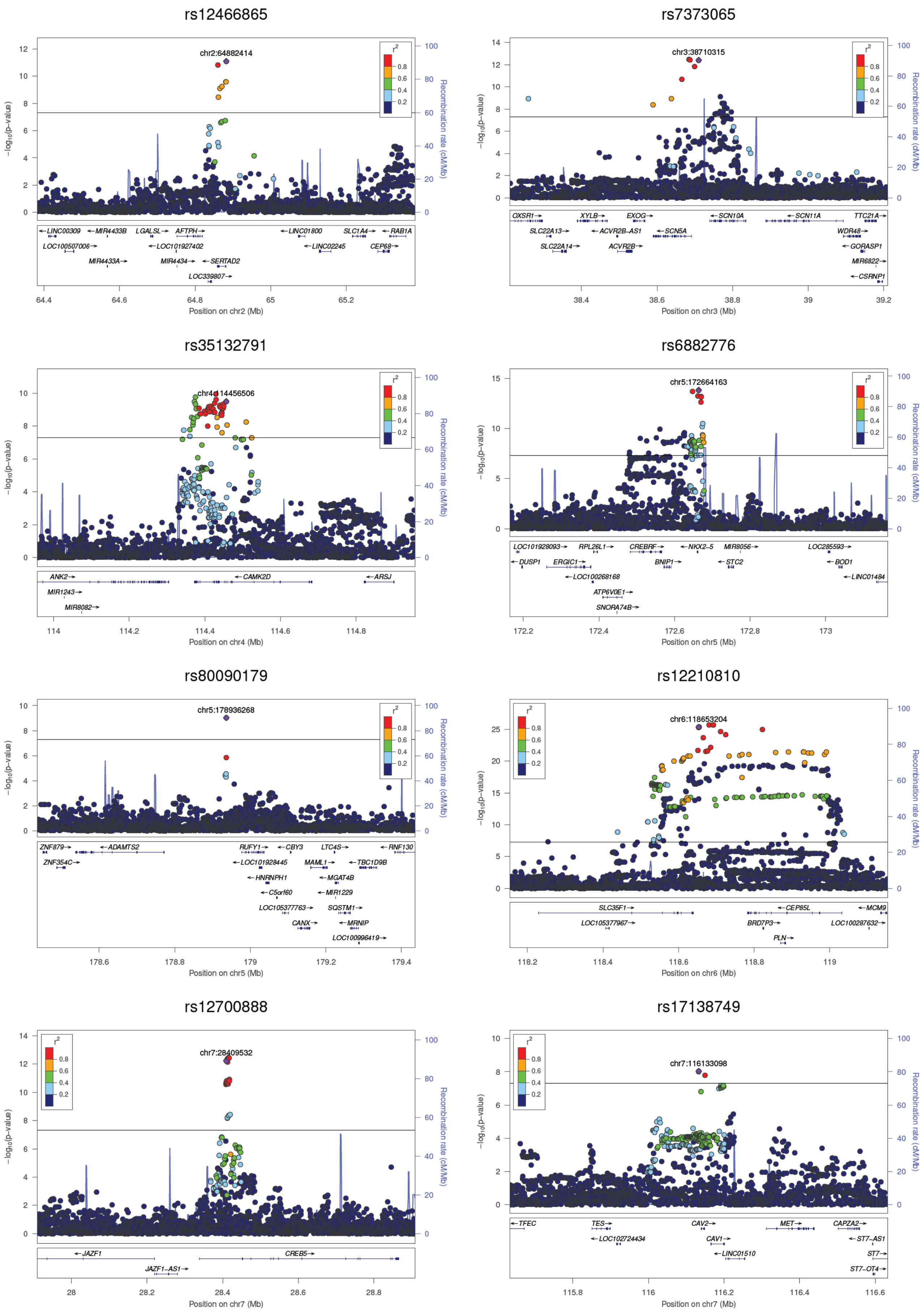
rs12466865
chr2:64882414
Position on chr2 (Mb)
LINC00309
MIR4433B
LGALSL
AFTPH
LINC01800
SLC1A4
RAB1A
LOC100507006
LOC101927402
LINC02245
CEP68
MIR4433A
MIR4434
SERTAD2
LOC339807
rs7373065
chr3:38710315
Position on chr3 (Mb)
OXSR1
XYLB
EXOG
SCN10A
SCN11A
TTC21A
SLC22A13
ACVR2B-AS1
SCN5A
WDR48
SLC22A14
ACVR2B
GORASP1
MIR6822
CSRNP1
rs35132791
chr4:114456506
Position on chr4 (Mb)
ANK2
CAMK2D
ARSJ
MIR1243
MIR8082
rs6882776
chr5:172664163
Position on chr5 (Mb)
LOC101928093
RPL26L1
CREBRF
NKX2-5
MIR8056
LOC285593
DUSP1
ERGIC1
BNIP1
STC2
BOD1
LOC100268168
LINC01484
ATP6V0E1
SNORA74B
rs80090179
chr5:178936268
Position on chr5 (Mb)
ZNF879
ADAMTS2
RUFY1
CBY3
LTC4S
RNF130
ZNF354C
LOC101928445
MAML1
TBC1D9B
HNRNPH1
MGAT4B
C5orf60
MIR1229
LOC105377763
SQSTM1
CANX
MRNIP
LOC100996419
rs12210810
chr6:118653204
Position on chr6 (Mb)
SLC35F1
CEP85L
MCM9
LOC105377967
BRD7P3
LOC100287632
PLN
rs12700888
chr7:28409532
Position on chr7 (Mb)
JAZF1
CREB5
JAZF1-AS1
rs17138749
chr7:116133098
Position on chr7 (Mb)
TFEC
TES
CAV2
MET
CAPZA2
LOC102724434
CAV1
ST7-AS1
LINC01510
ST7
ST7-OT4
−log10(p−value)
Recombination rate (cM/Mb)
r2
0.8
0.6
0.4
0.2

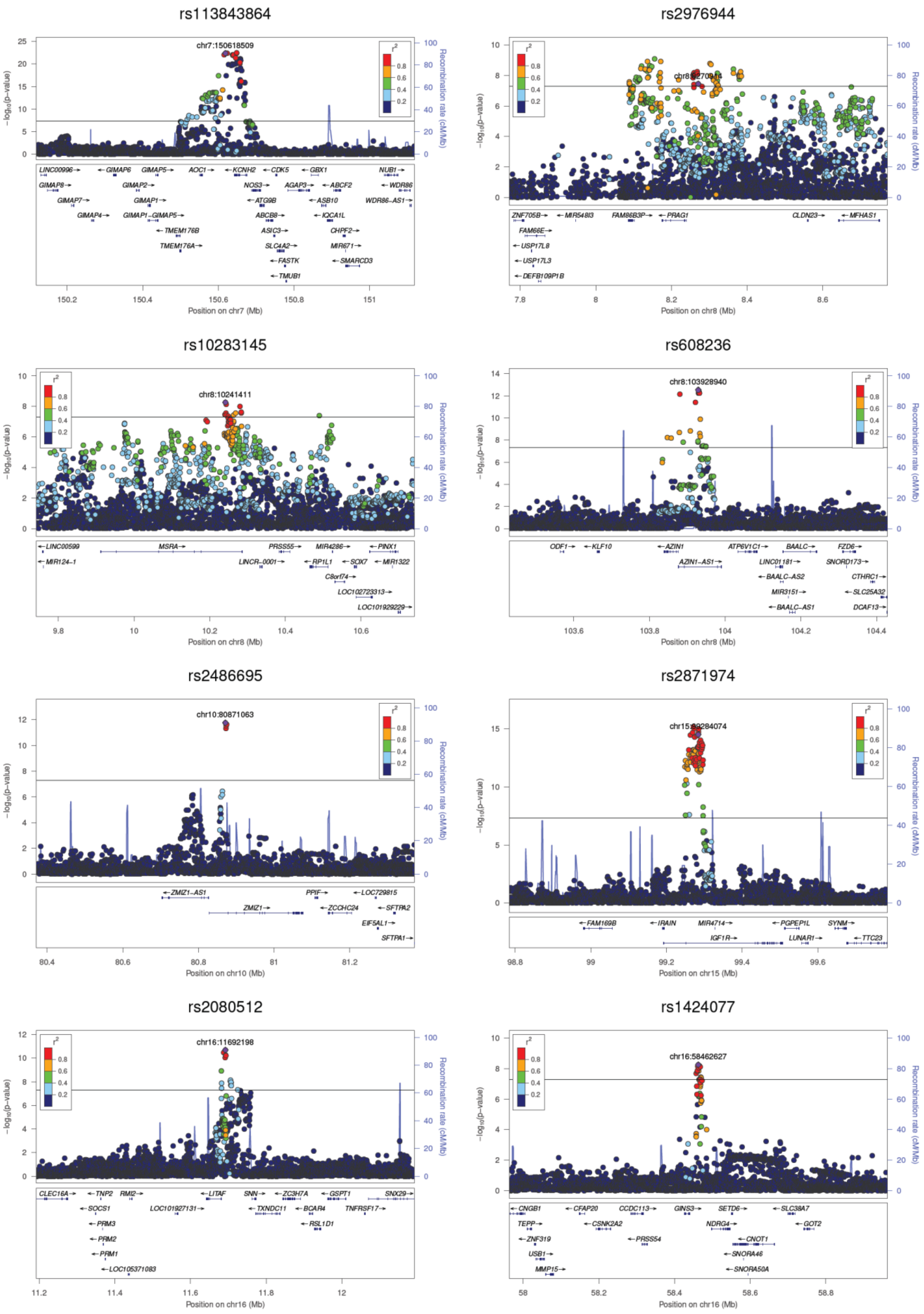
rs113843864
chr7:150618509
−log10(p−value)
Recombination rate (cM/Mb)
Position on chr7 (Mb)
LINC00996 GIMAP6 GIMAP5 AOC1 KCNH2 CDK5 GBX1 NUB1
GIMAP8 GIMAP2 NOS3 AGAP3 ABCF2 WDR86
GIMAP7 GIMAP1 ATG9B ASB10 WDR86−AS1
GIMAP4 GIMAP1−GIMAP5 ABCB8 IQCA1L
TMEM176B ASIC3 CHPF2
TMEM176A SLC4A2 MIR671
FASTK SMARCD3
TMUB1
rs2976944
chr8:8270914
Position on chr8 (Mb)
ZNF705B MIR548I3 FAM86B3P PRAG1 CLDN23 MFHAS1
FAM66E
USP17L8
USP17L3
DEFB109P1B
rs10283145
chr8:10241411
Position on chr8 (Mb)
LINC00599 MSRA PRSS55 MIR4286 PINX1
MIR124−1 LINCR−0001 RP1L1 SOX7 MIR1322
C8orf74
LOC102723313
LOC101929229
rs608236
chr8:103928940
Position on chr8 (Mb)
ODF1 KLF10 AZIN1 ATP6V1C1 BAALC FZD6
AZIN1−AS1 LINC01181 SNORD173
BAALC−AS2 CTHRC1
MIR3151 SLC25A32
BAALC−AS1 DCAF13
rs2486695
chr10:80871063
Position on chr10 (Mb)
ZMIZ1−AS1 PPIF LOC729815
ZMIZ1 ZCCHC24 SFTPA2
EIF5AL1
SFTPA1
rs2871974
chr15:99284074
Position on chr15 (Mb)
FAM169B IRAIN MIR4714 PGPEP1L SYNM
IGF1R LUNAR1 TTC23
rs2080512
chr16:11692198
Position on chr16 (Mb)
CLEC16A TNP2 RMI2 LITAF SNN ZC3H7A GSPT1 SNX29
SOCS1 LOC101927131 TXNDC11 BCAR4 TNFRSF17
PRM3 RSL1D1
PRM2
PRM1
LOC105371083
rs1424077
chr16:58462627
Position on chr16 (Mb)
CNGB1 CFAP20 CCDC113 GINS3 SETD6 SLC38A7
TEPP CSNK2A2 NDRG4 GOT2
ZNF319 PRSS54 CNOT1
USB1 SNORA46
MMP15 SNORA50A

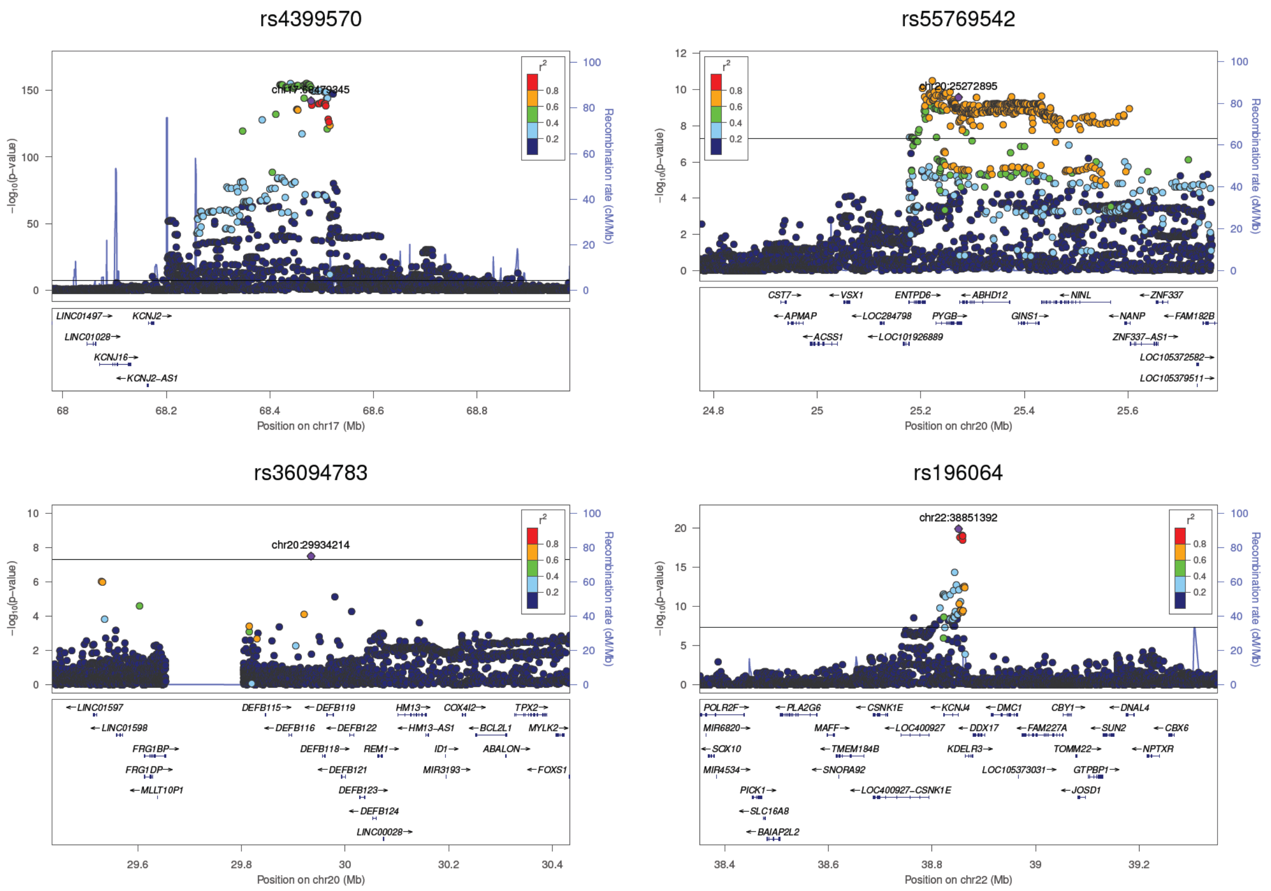

Figure S6: Locus Zoom plots for the twenty-eight (ordered by chromosome and base pair position) identified lead SNVs for resting Tpe.

rs607484

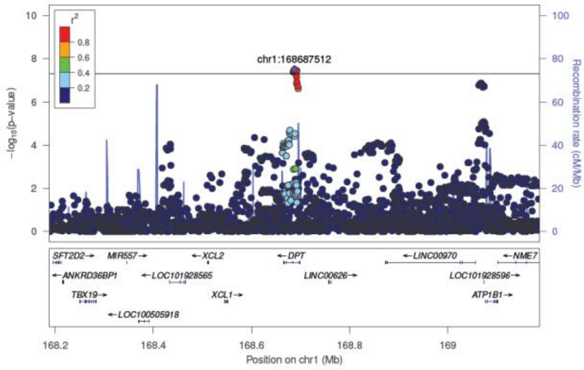

rs761499672

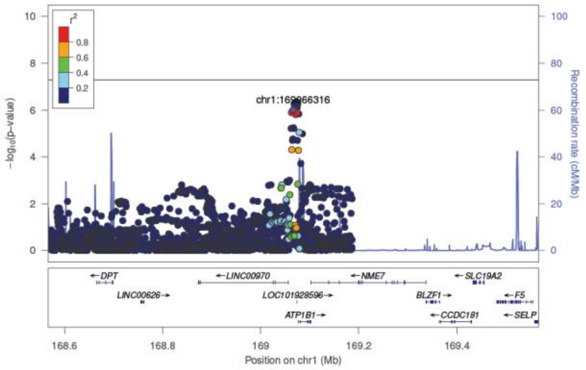

rs7373065

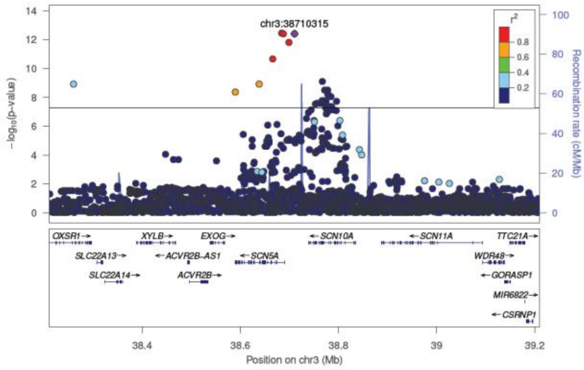

rs6801957

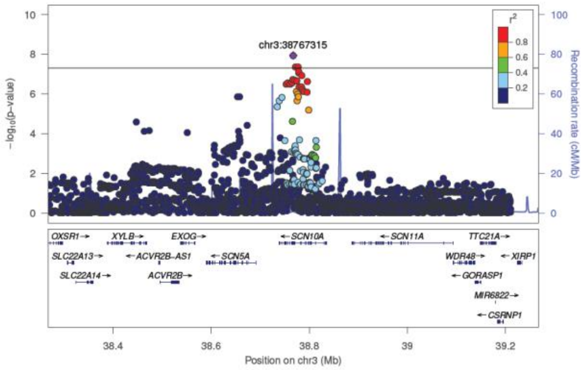

rs2080512

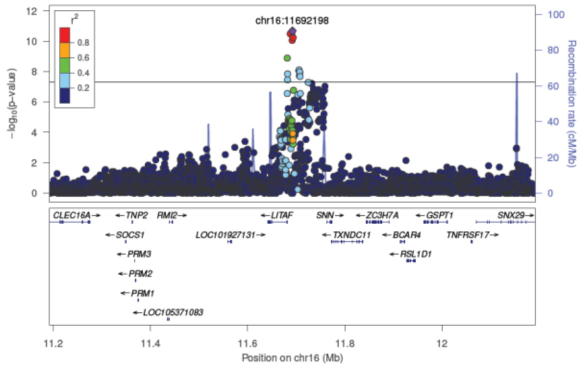

rs570620219

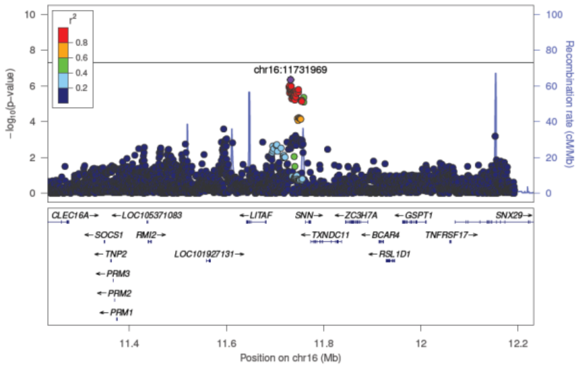

rs7373065

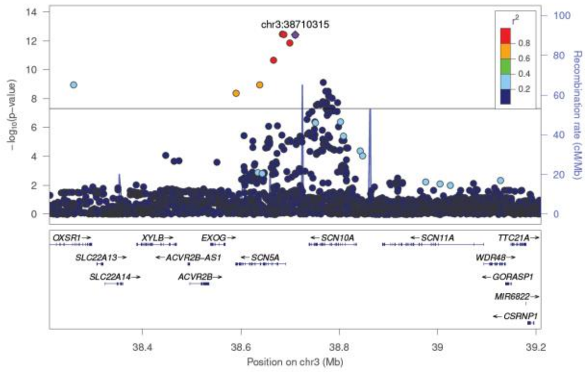

rs6801957

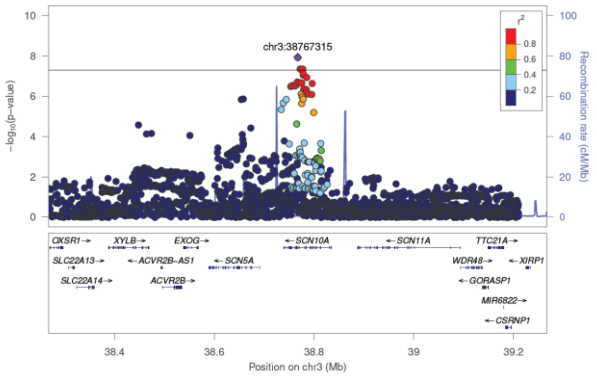

Figure S7: Locus Zoom plots for the four loci with secondary signals for resting Tpe.

Left: lead SNV. Right: LZ plot conditioned on the lead SNV.

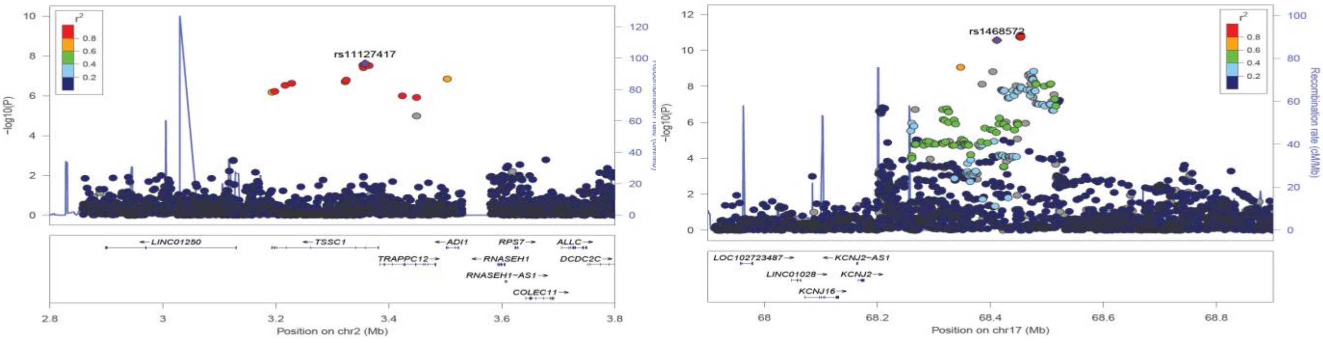

Figure S8: Locus Zoom plots for the identified loci for Tpe dynamics during exercise.

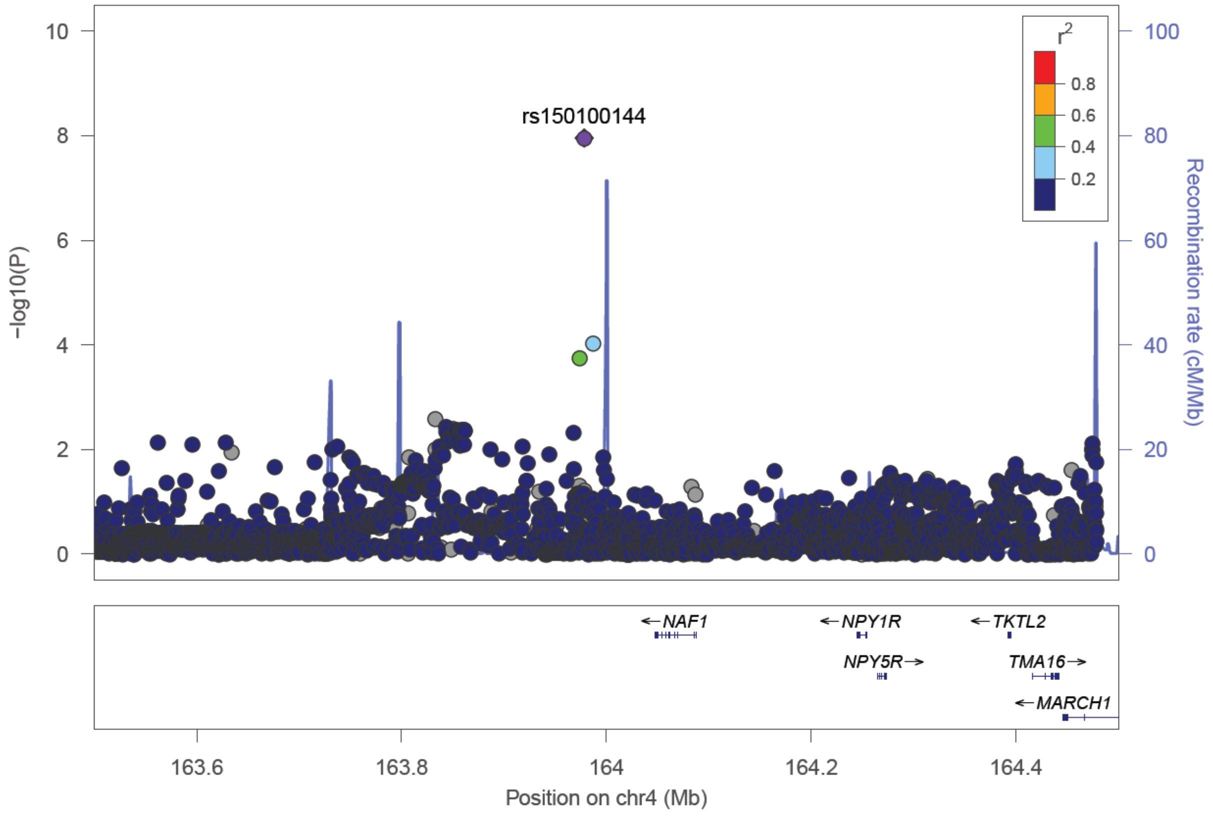

Figure S9: Locus Zoom plots for the identified locus for Tpe dynamics during recovery.

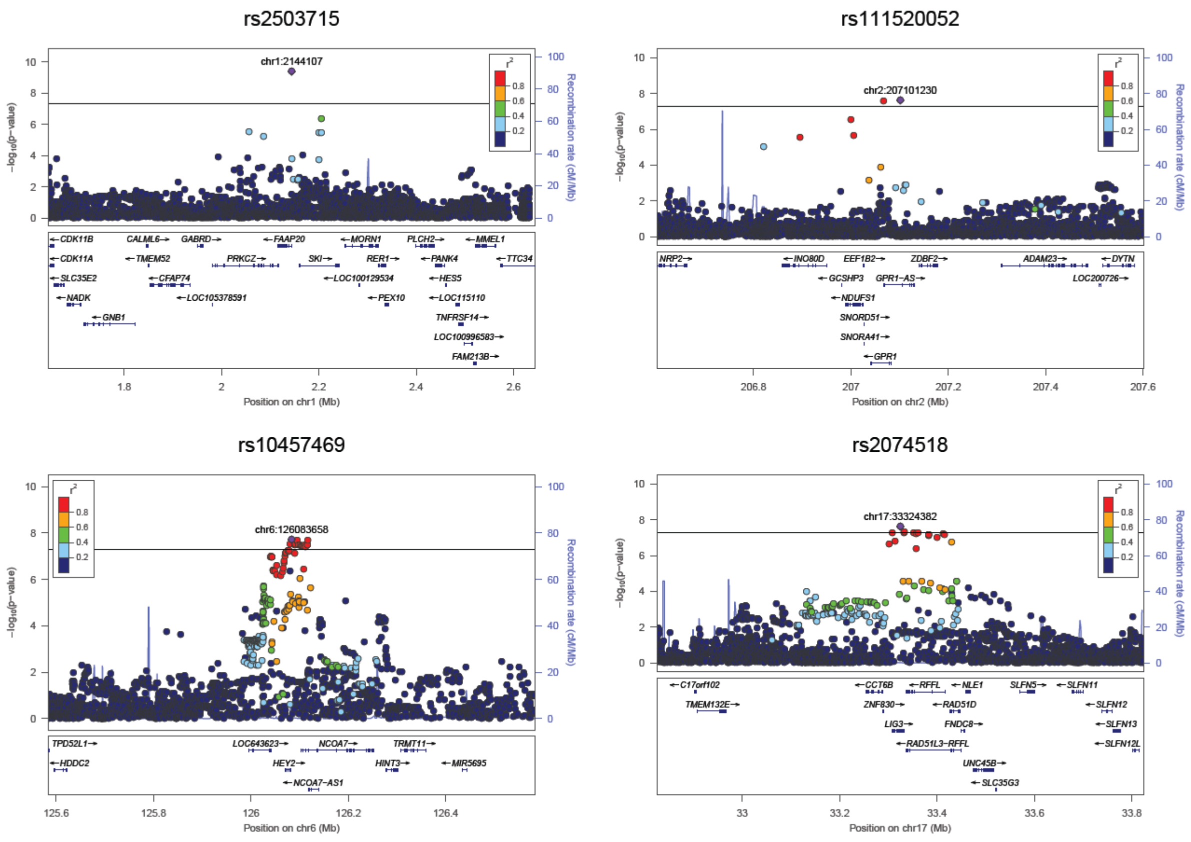

Figure S10: Locus Zoom plots for the male-specific loci for resting Tpe (males only summary statistics).

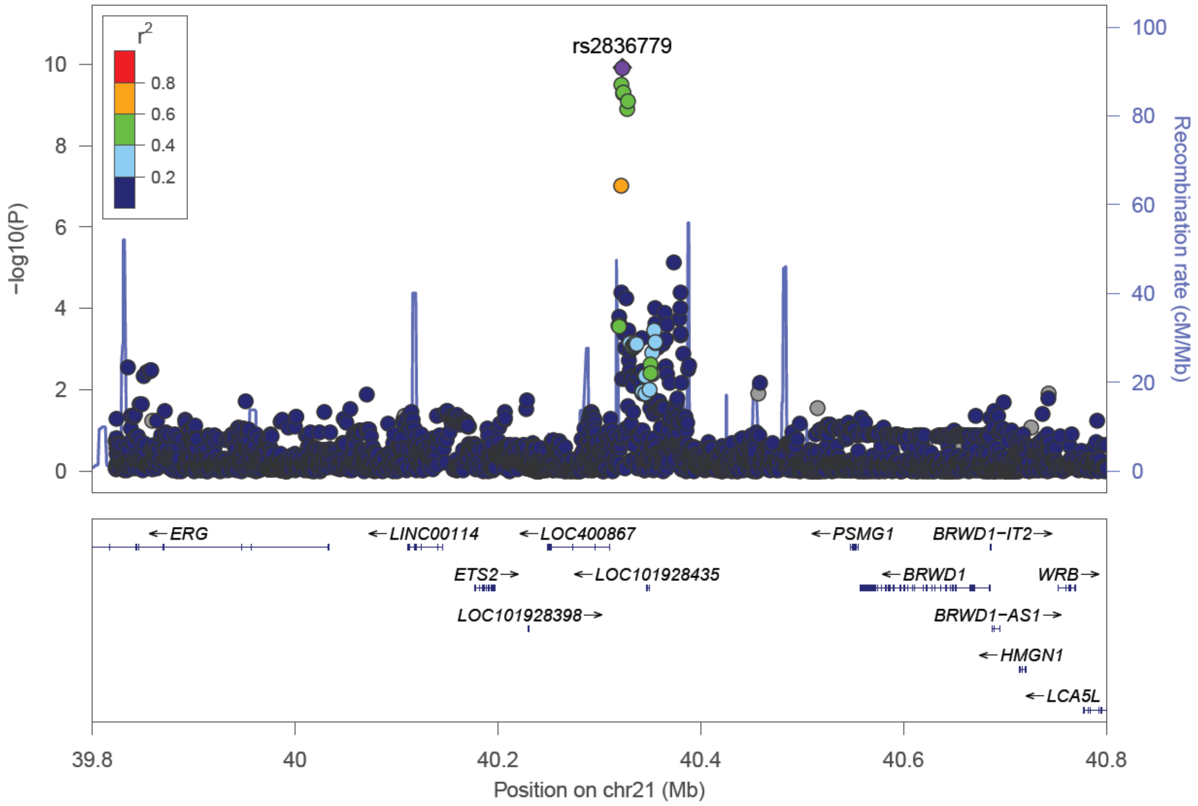

Figure S11: Locus Zoom plots for the sex-specific locus for Tpe dynamics during exercise (males only summary statistics).

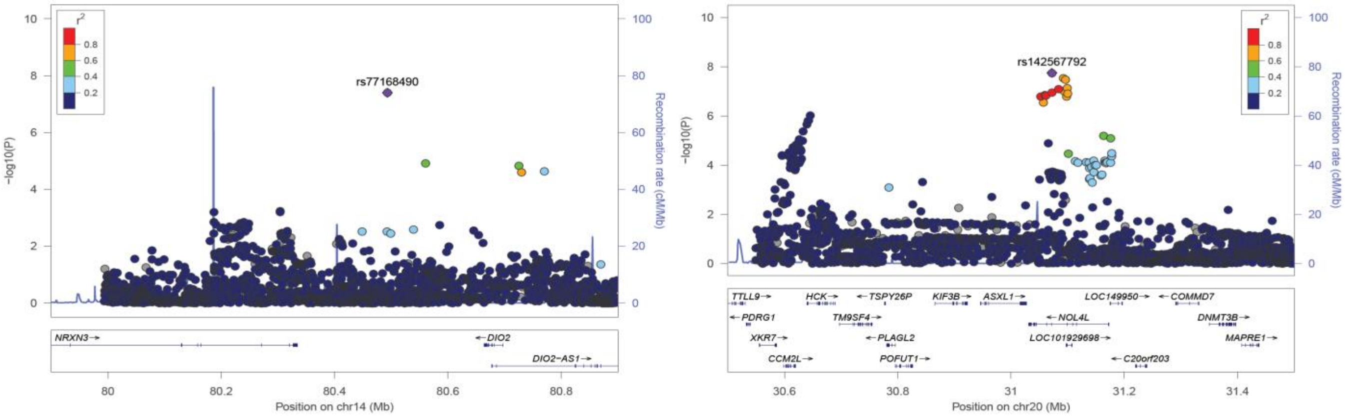

Figure S12: Locus Zoom plots for the sex-specific loci for Tpe dynamics during recovery (females only summary statistics).

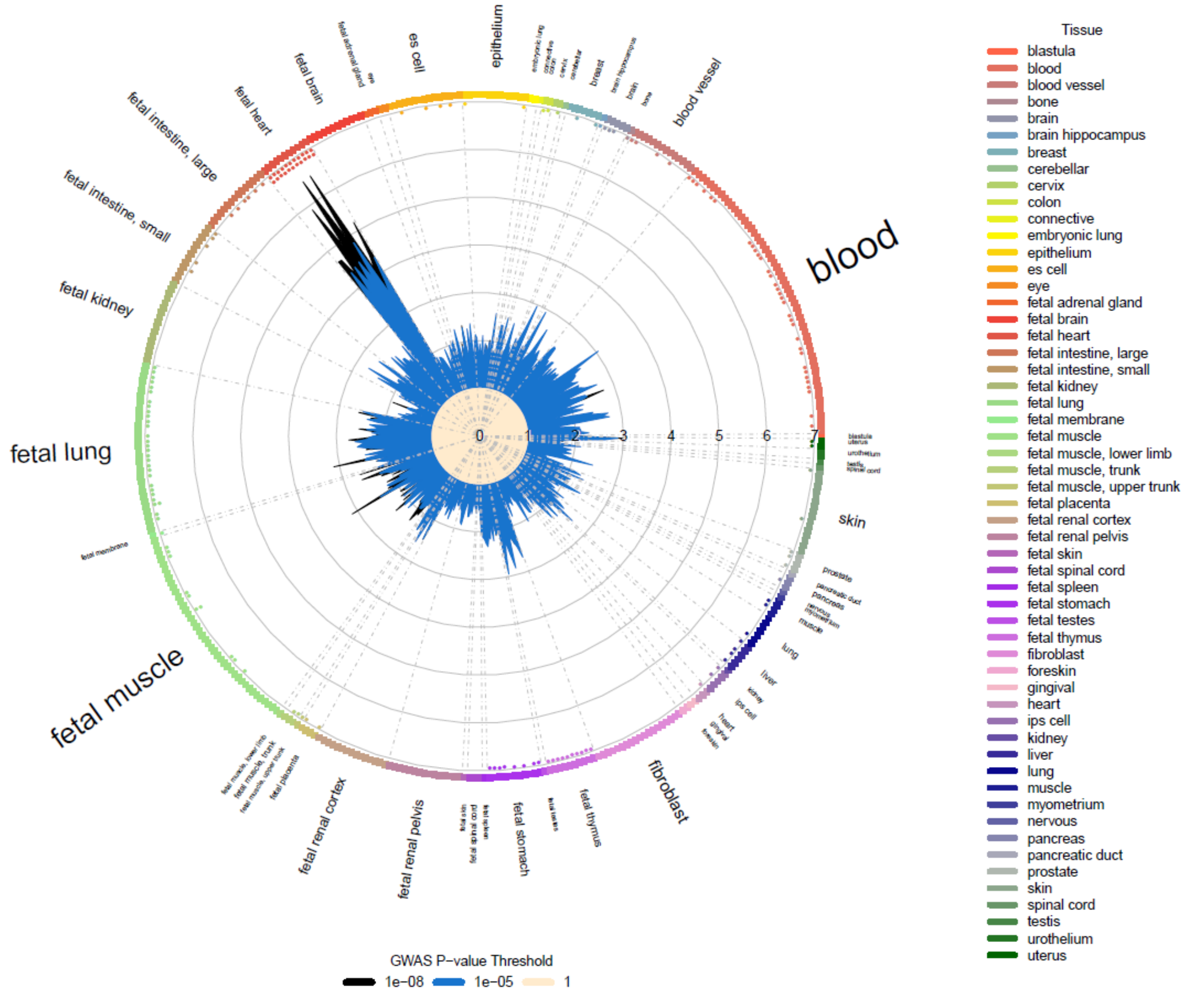

Figure S13: Functional enrichment analysis of resting Tpe loci within DNaseI hypersensitivity spots.

The radial lines show fold enrichment (FE) at eight GWA *P*-value thresholds. The results are shown for each of 424 cell types which are sorted by tissue, represented along the outer circle of the plot. The font size is proportional to the number of cell types from the tissue. FE values are plotted with different colours with respect to different GWA thresholds. Significant enrichment for a given cell type is denoted along the outer circle of the plot from a GWA *P*-value threshold.

source	term name	term ID	n. of term genes	n. of query genes	n. of common genes	corrected p-value	EIPR1	KCNJ2	ETS2
cor	ETS2-ETS1 complex	CORUM:2790	2	1	1	3.11e-03	?	?	co
cor	ETS2-ERG complex	CORUM:2789	2	1	1	3.11e-03	?	?	co
cor	ETS2-SMARCA4-INI1 complex	CORUM:5293	3	1	1	4.66e-03	?	?	co
cor	ETS2-FOS-JUN complex	CORUM:2695	3	1	1	4.66e-03	?	?	co
rea	Classical Kir channels	R-HSA-1296053	4	2	1	1.51e-02	?	r	
mir	hsa-miR-196b-5p	hsa-miR-196b-5p	151	3	2	1.93e-02		mi	mi
mir	hsa-miR-3646	hsa-miR-3646	199	3	2	3.35e-02		mi	mi
BP	regulation of skeletal muscle contraction by action potential	GO:0100001	1	2	1	3.77e-02	?	M	
BP	regulation of skeletal muscle contraction via regulation of action potential	GO:0014861	1	2	1	3.77e-02	?	M	

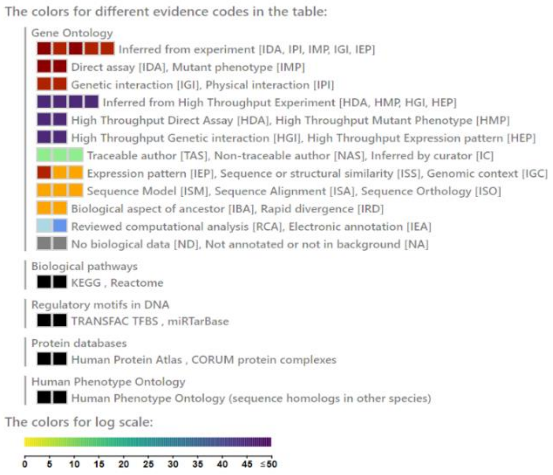

Figure S14: Top biological processes enrichment of candidate genes at Tpe dynamics during exercise loci.

g:profiler GO (gene ontology) term enrichment was performed using the candidate genes for Tpe dynamics during exercise.

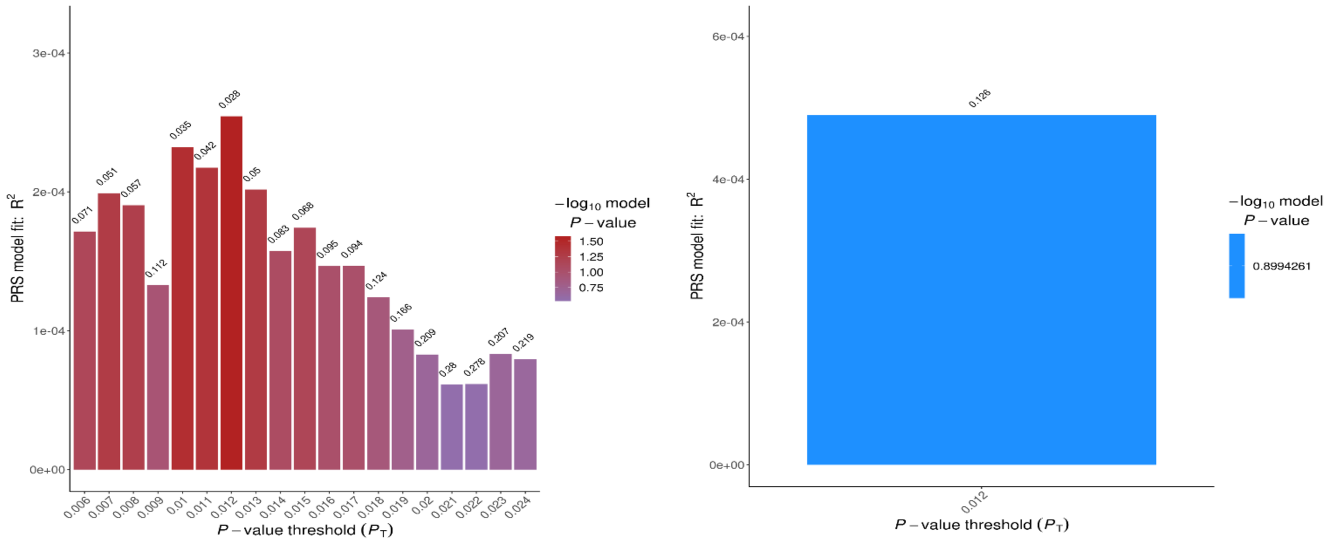

Figure S15: Left, adjusted R-squared versus the *P*-value threshold in the training subset (N = 247,256). The optimal cut-off value is the one for which the adjusted R-squared is highest and the *P*-value is lowest (*P* = 0.012). Right, adjusted R-squared and *P*-value of the logistic regression in the validation set (N = 68,563) using the optimal cut-off value.

Supplemental Tables

Locus	SNV	CHR	BP	EA	MAF	Reported *P*	*P* in our study	N	β (ms)	PMID
NOS1AP	rs2880058	1	162014632	A	-	1.00E-02	2.90E-03	5,890	-0.6	20215044
	rs4657139	1	162029907	T	-	3.00E-02	9.60E-04	5,890	-0.5	20215044
	rs10918594	1	162030688	C	-	2.00E-03	1.40E-03	5,890	-0.8	20215044
	rs10494366	1	162085685	T	-	2.00E-02	1.70E-04	5,890	-0.6	20215044
KCNH2	rs1805123	7	150645534	A	-	5.00E-05	4.30E-22	5,890	-1.2	20215044
	rs3807375	7	150667210	G	-	1.00E-03	2.20E-13	5,890	0.8	20215044
KCNE1	rs1805128	21	35821680	G	-	2.00E-01	1.40E-01	5890	-1.300	20215044
GRIN2A	rs17749681	16	9713930	C	0.380	2.10E-07	5.70E-01	1870	1.450	22342860

KCNJ2	**rs7219669**	**17**	**70525720**	**T**	**0.380**	**1.10E-10**	**4.20E-148**	**1870**	**-1.790**	**22342860**

Table S1: Previously-reported loci associated with resting Tpe interval.

Abbreviations: SNV: single-nucleotide variation, CHR: Chromosome, BP: Position, based on HG build 19, EA: Effect allele, EAF: Effect allele frequency from discovery data, β: Beta, SE: Standard Error, N: number of participants, P: P-value.

The locus name indicates the gene that is in the closest proximity to the most associated SNV.

Bold indicates replicated in an independent study.

Only one genome-wide significant variant has been previously reported.

Code	Definition
I460	Cardiac arrest with successful resuscitation
I461	Sudden cardiac death, so described
I469	Cardiac arrest, unspecified
I472	Ventricular tachycardia
I490	Ventricular fibrillation and flutter
I499	Cardiac arrhythmia, unspecified

Table S2: ICD-10 codes used in follow-up analysis

						MALES				FEMALES				COMBINED			
Locus	SNV	CHR	BP	EA	EAF	β	SE	P	N	β	SE	P	N	β	SE	P	N
FAAP20	rs2503715	1	2144107	A	0.130	-0.072	0.012	4.20E-10	30532	-0.017	0.011	1.20E-01	34618	-0.043	0.008	6.20E-08	65150
RPL22	rs10864434	1	6262231	A	0.599	-0.052	0.008	1.70E-11	32176	-0.064	0.007	4.60E-18	36482	-0.058	0.005	5.80E-28	68658
SSBP3	rs562408	1	54742618	A	0.433	-0.052	0.008	4.60E-12	32610	-0.046	0.007	1.40E-10	36974	-0.049	0.005	3.20E-21	69584
SGIP1	rs10789207	1	66991346	T	0.785	-0.083	0.009	3.30E-20	33160	-0.026	0.009	2.50E-03	37597	-0.054	0.006	7.50E-18	70757
MEF2D	rs1050316	1	156434703	G	0.346	-0.074	0.008	2.90E-21	33270	-0.052	0.007	3.40E-12	37722	-0.061	0.005	9.40E-30	70993
STRN	rs66993681	2	37118665	C	0.456	0.026	0.008	6.50E-04	33012	0.040	0.007	3.10E-08	37430	0.033	0.005	2.20E-10	70441
SLC8A1	rs35450971	2	40754314	T	0.935	0.034	0.015	2.30E-02	32988	0.085	0.014	5.00E-09	37403	0.062	0.010	3.30E-09	70392
SERTAD2	2:64860029_CTTCAAA_C	2	64860029	CTTCAAA	0.669	-0.032	0.008	4.70E-05	32360	-0.043	0.008	1.60E-08	36690	-0.037	0.006	1.40E-11	69050
GPR1	rs111520052	2	207101230	G	0.988	0.190	0.034	2.40E-08	33011	0.056	0.033	9.10E-02	37429	0.117	0.024	7.30E-07	70440
SCN5A	rs7373065	3	38710315	T	0.019	-0.126	0.028	5.40E-06	31088	-0.155	0.027	7.90E-09	35248	-0.140	0.019	3.80E-13	66336
NKX2-5	rs6884881	5	172673319	T	0.433	-0.042	0.008	2.60E-08	32465	-0.026	0.007	2.60E-04	36810	-0.034	0.005	6.70E-11	69275
SLC35F1	rs12210810	6	118653204	G	0.945	0.124	0.016	2.60E-14	33432	0.114	0.016	2.00E-13	37906	0.118	0.011	4.60E-26	71338
HEY2	rs10457469	6	126083658	G	0.476	-0.042	0.007	1.90E-08	33350	-0.009	0.007	2.20E-01	37813	-0.024	0.005	1.80E-06	71162
CREB5	7:28413064_CT_C	7	28413064	CT	0.252	0.051	0.009	4.00E-09	32058	0.037	0.008	8.50E-06	36348	0.043	0.006	7.20E-13	68406
KCNH2	rs148064265	7	150626596	G	0.792	-0.068	0.009	1.10E-13	32897	-0.050	0.009	1.70E-08	37299	-0.059	0.006	1.20E-20	70196
AZIN1	rs608236	8	103928940	A	0.433	0.033	0.008	8.50E-06	33025	0.041	0.007	1.10E-08	37445	0.038	0.005	2.80E-13	70471
ZMIZ1	rs1658323	10	80874523	C	0.613	-0.030	0.008	8.30E-05	32152	-0.044	0.007	3.70E-09	36455	-0.038	0.005	2.20E-12	68607
IGF1R	rs4965430	15	99268850	C	0.375	-0.045	0.008	3.70E-09	33110	-0.040	0.007	9.00E-08	37541	-0.042	0.005	2.00E-15	70650
LITAF	rs2080512	16	11692198	G	0.538	-0.026	0.007	4.40E-04	33238	-0.041	0.007	5.80E-09	37686	-0.034	0.005	2.00E-11	70924
LIG3	rs2074518	17	33324382	C	0.545	0.042	0.007	2.40E-08	33432	0.009	0.007	1.90E-01	37906	0.025	0.005	1.60E-06	71338
KCNJ2	rs4399570	17	68479345	G	0.699	0.148	0.008	2.20E-74	33301	0.137	0.008	7.40E-71	37757	0.142	0.006	5.30E-143	71058
PYGB	rs3787080	20	25232604	C	0.741	-0.018	0.009	3.20E-02	33407	-0.051	0.008	1.50E-10	37878	-0.036	0.006	6.50E-10	71285
KCNJ4	rs196064	22	38851392	C	0.631	0.052	0.008	1.40E-11	33203	0.045	0.007	6.50E-10	37646	0.049	0.005	1.30E-20	70848

Table S4A: Sex-stratified analyses for resting Tpe interval.

Lead SNVs at *FAAP20*, *GPR1*, *HEY2* and *LIG3* (indicated in yellow) are male specific loci. These variants are non-significant (*P* > 0.05) in females only. They were not selected for replication in the discovery analysis, and were non-significant in the combined analyses.

						MALES				FEMALES				COMBINED			
Locus	SNV	CHR	BP	EA	EAF	β	SE	P	N	β	SE	P	N	β	SE	P	N
ETS2	**rs2836779**	**21**	**40322678**	**C**	**0.353**	**0.061**	**0.009**	**1.20E-10**	**24241**	**0.007**	**0.009**	**4.10E-01**	**27269**	**0.032**	**0.006**	**5.70E-07**	**51510**

Table S4B: Sex-stratified analyses for Tpe dynamics during exercise.

Lead SNV at *ETS2* (indicated in bold type) is a male specific locus. This variant is non-significant (*P* > 0.05) in females only. It was not selected for replication in the discovery analysis, and was non-significant in the combined analyses.

Abbreviations: SNV: single-nucleotide variant CHR: chromosome, BP: Base pair position, based on HG built 18, EA: effect allele, EAF: effect allele frequency, β: Beta in beats per minute, SE: Standard Error, N: effective number of participants, P: P-value.

						MALES				FEMALES				COMBINED			
Locus	SNV	CHR	BP	EA	EAF	β	SE	P	N	β	SE	P	N	β	SE	P	N
NRXN3	rs77168490	14	80493255	A	0.98	-0.032	0.033	3.26E-01	24172	-0.170	0.031	4.00E-08	27331	-0.102	0.022	5.00E-06	51503
NOL4L	rs565497590	20	31049031	G	0.987	0.025	0.040	5.22E-01	23377	0.214	0.037	1.10E-08	26432	0.119	0.027	8.90E-06	49808

Table S4C: Sex-stratified analyses for Tpe dynamics during recovery.

Lead SNVs at *NRXN3* and *NOL4L* (indicated in yellow) are female specific loci. The variants were not selected for replication from the discovery analyses and were non-significant in the combined analyses.

Abbreviations: SNV: single-nucleotide variant CHR: chromosome, BP: Base pair position, based on HG built 18, EA: effect allele, EAF: effect allele frequency, β: Beta in beats per minute, SE: Standard Error, N: effective number of participants, P: P-value.

									$p_{12} = 1 \times 10^{-6}$	
**Locus**	**Lead SNV**	**CHR**	**BP**	**eQTL SNV**	**r2 (Lead SNV-eQTL SNV)**	**eQTL P-value**	**Tissue**	**Transcript**	**PP Different signal%**	**PP common signal%**
SSBP3	rs603901	1	54741767	rs590041	0.990	1.48E-10	Heart_Left_Ventricle	*SSBP3*	6	94
				rs562408	0.990	6.84E-23	Heart_Atrial_Appendage	*SSBP3*	5	95
SGIP1	rs10789207	1	66991346	rs72677052	0.995	9.43E-26	Heart_Left_Ventricle	*SGIP1*	7	93
NKX2-5	rs6882776	5	172664163	rs6891790	0.934	2.29E-09	Brain_Cerebellum	*NKX2-5*	9	91
HEY2	rs10457469	6	126083658	rs3757217	0.990	2.41E-13	Brain_Anterior_cingulate_cortex_BA24	*RP11-624M8.1*	35	60
				rs1811852	0.990	7.87E-09	Heart_Atrial_Appendage	*RP11-624M8.1*	37	57
				rs980014	0.979	4.04E-12	Brain_Amygdala	*RP11-624M8.1*	32	64
MSRA	rs10283145	8	10241411	rs6601450	0.890	4.03E-08	Brain_Hippocampus	*RP11-981G7.6*	40	59
IGF1R	rs2871974	15	99284074	rs6598541	0.972	5.30E-09	Heart_Left_Ventricle	*IGF1R*	6	94
LITAF	rs2080512	16	11692198	rs735951	0.992	9.85E-10	Heart_Left_Ventricle	*LITAF*	4	96
GINS3	rs1424077	16	58462627	rs2042401	0.999	9.62E-17	Brain_Caudate_basal_ganglia	*RP11-481J2.2*	8	92
				rs4784934	0.993	1.63E-17	Brain_Putamen_basal_ganglia	*RP11-481J2.2*	12	88
				rs9928581	0.996	3.15E-13	Heart_Atrial_Appendage	*NDRG4*	11	89
LIG3	rs2074518	17	33324382	rs12945428	0.948	7.04E-24	Heart_Left_Ventricle	*LIG3*	14	83
						7.30E-18	Brain_Cortex	*LIG3*	13	85
				rs2339123	0.934	4.07E-10	Brain_Anterior_cingulate_cortex_BA24	*LIG3*	15	82
				rs1003918	0.992	2.92E-12	Brain_Caudate_basal_ganglia	*LIG3*	17	80
						5.61E-17	Brain_Cerebellar_Hemisphere	*LIG3*	12	85
						5.61E-20	Brain_Cerebellum	*LIG3*	14	84
						3.27E-09	Brain_Hippocampus	*LIG3*	14	84
				rs978202	0.874	2.93E-11	Brain_Frontal_Cortex_BA9	*LIG3*	14	84
				rs1088450	0.933	4.01E-11	Brain_Hypothalamus	*LIG3*	13	85
				rs2074518	1.000	8.12E-12	Brain_Nucleus_accumbens_basal_ganglia	*LIG3*	12	85
						2.09E-09	Brain_Putamen_basal_ganglia	*LIG3*	13	85
						8.91E-23	Heart_Atrial_Appendage	*LIG3*	12	85

Table S5: Expression quantitative trait locus (eQTL) analysis for resting Tpe interval.

Resting Tpe variants with significant eQTLs and its corresponding genes are indicated. The results from proxy variants, with high LD ($r^2 \geq 0.8$) with the lead variant in the UK Biobank study were included if there was tissue expression data in addition to the lead variant. Results were filtered to those reaching a P value ≤ 5×10^{-8}. The source was Genotype-Tissue Expression (GTEx) Consortium v7, PubMed ID is 25954001. r^2: A measure for the linkage disequilibrium between the proxy and lead SNVs; P: P value for the association between the variant and RNA tissue expression.

Columns J and K show the posterior probability of different signal and common signal after applying colocalisation test at a prior probability of 1 x 10-6 that a variant is associated with both traits.

Yellow rows indicate loci with tissue specific eQTLs with a strong colocalisation support.

MeSH term	Name	MeSH first level term	MeSH second level term	Nominal P value	False discovery rate
A07.541	Heart	Cardiovascular System	Heart	1.87E-04	<0.01
A07.541 .560	Heart Ventricles	Cardiovascular System	Heart	2.38E-04	<0.01
A07.541 .358	Heart Atria	Cardiovascular System	Heart	4.45E-04	<0.05

Table S8: DEPICT tissue enrichment across all resting Tpe loci.

Supplemental Methods

Phenotypic and genetic QC

In the EST-UKB cohort, the exercise protocol was adapted according to participants' risk factors. Participants were only included in the study if they were allowed to cycle at 50% or 30% of their maximum workload (no risk to minimum risk). If the heart rate reached the pre-set maximum heart rate level (75% of age-predicted maximum heart rate), the test was stopped. Also, if the participant reported chest pain, felt faint, dizzy or unwell, the test was also stopped (https://biobank.ctsu.ox.ac.uk/crystal/docs/Cardio.pdf). We only included participants who terminated the exercise stress test with any discomfort and with a heart rate lower than the pre-set maximum heart rate level.

Individuals were excluded based on existing medical conditions known to affect heart rate (atrial fibrillation, history of myocardial infarction or heart failure, (supra)-ventricular tachycardia, atrioventricular nodal re-entrant tachycardia, second or third degree atrioventricular block, bundle branch block and use of a pacemaker), individuals with a previous cardiovascular event (matching the codes from Supplemental Table 1) and/or individuals on heart rate altering medications (non-dihydropyridine calcium antagonists (Anatomic Therapeutic Chemical (ATC) code C08D, digoxin (ATC code C01AA5), and amiodarone (ATC code C01BD01)). Individuals with an RR interval (inverse of heart rate) change between resting and peak exercise, or between peak exercise and recovery, less than 10 ms or poor quality ECG recording were also excluded.

Individuals with bad genotype quality, provided by UKB, i.e. high missingness or heterozygosity and discordance between the self-reported sex and the sex inferred from the genotypes were excluded[1]. We used the k-means function in R as a clustering algorithm, to objectively and statistically select the clusters according to information from PC1 and PC2. The k-means algorithm 'partitions the points into k groups such that the sum of squares from points to the assigned cluster centres in minimised'. Then, we applied k-means separately to

cluster according to each PC1 and PC2, and initially only with k=4, for a 4-way clustering, to correspond to the 4 main ethnic clusters within UKB: White, African, Asian and Chinese. We then created an overall clustering, according to the intersections of the PC1-kmeans clustering and the PC2-4means clustering, so that participants were only categorised as ‘White’ overall, if they were contained in the ‘White’ cluster for both PCA1 and PC2. Next, we created an overall ‘Mixed/Other’ cluster, for any participants, whose clustering differed between PC1 and PC2. Finally, we combined the PCA ancestry clusters with the self-reported ethnicity. Individuals were only included if the PCA-clustering results matched the self-reported ancestry. However, we count ‘mixed’, ‘other’ and ‘missing’ as being broad/uncertain self-reported ethnicity, which have now been validated more objectively from the genetic PCA data. We restricted our genetic analyses to individuals with European ancestry.

Genetic analyses

For each trait, we carefully selected covariates based on “a priori” physiological knowledge of the marker, and additional confounding factors were identified using linear regression. Selection of covariates was performed for each trait specifically. The main rationale is that the three traits are physiologically different. For example, resting Tpe quantifies the later stage of ventricular repolarization at rest, which is closely modulated by resting RR interval. In contrast, Tpe response to exercise and recovery quantify the changes in Tpe with exercise and recovery which highly dependent on the corresponding changes in heart rate. We also checked the influence of additional confounding factors, such as smoking, alcohol or diabetes and only included those that were significantly associated.

ECG lead placement during the exercise test

The cardio assessment involved a 3 leads (lead I, II, and III) ECG recording (AM-USB 6.5, Cardiosoft v6.51) at a frequency of 500 Hz. The ECG was recorded using four electrodes placed on the right and left antecubital fossa and wrist (Figure R1) and stored in an xml-file of Cardiosoft (https://biobank.ctsu.ox.ac.uk/crystal/docs/Cardio.pdf).

Genetic risk score analyses

Ventricular arrhythmic risk was defined as arrhythmic mortality or admission to hospital with a ventricular arrhythmic diagnosis. The exact International Classification of Diseases, Tenth Revision codes used to define ventricular arrhythmic events are presented in Table S2. Date of death was obtained from death certificates held by the National Health Service (NHS) Information Centre and the NHS Central Register Scotland for participants from England and Wales and participants from Scotland, respectively. Diagnoses were captured using the "Spell and Episode" category from the Hospital Episode Statistics records. This category contains main and secondary diagnoses, coded according to *ICD-10*, made during the hospital inpatient stay. The main diagnoses are those taken to be the main reason for hospital admission, while secondary diagnoses are more often contributory or underlying conditions. We used both the main and secondary diagnoses for recording prevalent and incident risk factors, conditions and events. Date of the event was defined as the date of the first diagnosis.

Variants with minor allele frequency < 0.05 and imputation quality ≤ 0.3 were removed from the calculation. PRSice clumped variants to obtain SNVs in linkage equilibrium ($r^2 < 0.1$) within a 250 kb window. Multiple GRSs were computed at a large number of GWAS *P*-value thresholds ranging from 1×10^{-4} to 0.5 with 5×10^{-5} increments. PRSice then performed a logistic regression analysis between each GRS and ventricular arrhythmic risk, adjusting for age, sex, diabetes, cholesterol, BMI, systolic blood pressure (SBP), the genotyping array and the 10 first genetic principal components. The optimal GRS was then chosen as the one with the smallest *P*-value.

Supplemental References

1. Bycroft, C., Freeman, C., Petkova, D., Band, G., Elliott, L.T., Sharp, K., Motyer, A., Vukcevic, D., Delaneau, O., O'Connell, J., et al. (2017). Genome-wide genetic data on ~500,000 UK Biobank participants. bioRxiv.
